# Leveraging Influencers to Reach and Engage Vulnerable Individuals With a Digital Health Intervention: Quasi-Experimental Field Study

**DOI:** 10.2196/67174

**Published:** 2025-09-05

**Authors:** Marcia Nißen, Samira Harperink, Priyam Joshi, Anja Yvonne Bischof, Theresa Schachner, Yanick Xavier Lukic, Fabian Schneider, Prabhakaran Santhanam, Florian v Wangenheim, Joseph Ollier, Tobias Kowatsch

**Affiliations:** 1 Digital Health Interventions School of Medicine University of St. Gallen St.Gallen Switzerland; 2 Centre for Digital Health Interventions University of Zurich, ETH Zurich, University of St.Gallen Zurich Switzerland; 3 Institute for Implementation Science in Health Care University of Zurich Zurich Switzerland; 4 Chair of Technology Marketing Department of Management, Technology, and Economics ETH Zurich Zurich Switzerland; 5 PwC Switzerland Zurich Switzerland; 6 Department of Psychology Georgia State University Atlanta United States; 7 Institute of Computer Science School of Engineering ZHAW Zurich University of Applied Sciences Winterthur Switzerland; 8 Codeklang GmbH Bülach Switzerland; 9 Byldr GmbH Schlieren Switzerland; 10 Datahouse AG Zurich Switzerland; 11 Mobiliar Lab for Analytics Department of Management, Technology, and Economics ETH Zurich Zurich Switzerland

**Keywords:** noncommunicable diseases, prevention, low socioeconomic status, social milieux, vulnerable individuals, digital health interventions, influencer, thematic framing, gamification, storytelling, engagement, reach

## Abstract

**Background:**

Noncommunicable diseases are the leading cause of death, present economic challenges to health care systems worldwide, and disproportionally affect vulnerable individuals with low socioeconomic status (SES). While digital health interventions (DHIs) offer scalable and cost-effective solutions to promote health literacy and encourage behavior change, key challenges concern how to effectively reach and engage vulnerable individuals. To this end, social media influencers provide a unique opportunity to reach millions, and lasting engagement can be ensured through the design of DHIs in a manner that specifically appeals to low-SES individuals through alignment with their social background.

**Objective:**

The objectives of this study were 2-fold: to assess the effectiveness of leveraging influencers to reach vulnerable individuals (as measured via app downloads per stream viewers) and evaluate how the design of a DHI can improve engagement among this group (as measured via completion of the intervention).

**Methods:**

This study used a cross-sectional, quasi-experimental field design to assess both (1) the effectiveness of influencers in reaching vulnerable individuals and (2) the impact of specific design elements—such as gamification and storytelling—on user engagement using a stress management DHI featuring a slow-paced breathing exercise. In total, 3 differently designed versions of this DHI were developed following a fractional factorial design (StressLess, Breeze, and TragicKingdom). Reach was calculated as the number of downloads per viewers per stream and influencer. Engagement with the DHI was measured via number of conversational turns and milestone and intervention completion rates. Participants’ SES and technology acceptance were evaluated through a postintervention survey. Descriptive statistics, chi-square tests, and ANOVAs were used to examine the effects of the DHI design on reach and engagement metrics.

**Results:**

The recruitment via 8 influencers (total streams=25; total viewers=12,667) generated 220 downloads. The average reach ratio across streams amounted to 16.2% (SD 15.5%), with significant differences between conditions (*ꭓ*^2^_2_=8.0, *P*=.02; StressLess: 8.1%, SD 9.3%; Breeze: 14%, SD 10.5%; TragicKingdom: 28.4%, SD 17.6%). The intervention completion rate across all DHI versions amounted to 7.7% (17/220), with no significant differences between conditions (*P*=.48).

**Conclusions:**

This work provides the first evidence that recruitment via influencers yields high reach ratios, moving far beyond the reach of traditional social media platforms. Nonetheless, based on the data collected, the ability to leverage such platforms to recruit vulnerable individuals remains unclear. In addition, while engagement with the promoted interventions was initially high, the completion rate of the full breathing exercise was comparably low, indicating that the influencer promotion strategy cannot fully overcome the well-documented adherence barriers in digital health.

## Introduction

### Socioeconomic Disparities in Noncommunicable Diseases and the Digital Health Divide

Noncommunicable diseases (NCDs) are a major global health concern, responsible for more than 74% of deaths worldwide and significantly burdening health care systems [1]. In Europe, for example, cardiovascular diseases, cancer, respiratory diseases, and diabetes account for 75% of the disease burden within the European Union alone [2]. Beyond behavioral and environmental risk factors, growing evidence highlights the role of chronic psychological stress as a major contributor to the onset and progression of various NCDs [3-6] through mechanisms such as systemic inflammation, immune dysregulation, and metabolic dysfunction [6].

This burden is not confined to older populations but increasingly also affects younger vulnerable individuals, especially those with low socioeconomic status (SES)—a latent construct encompassing educational level, income, and occupational status [7]. Low-SES individuals are often disproportionately exposed to chronic stressors related to financial insecurity, precarious employment, and limited access to preventive health services [8-10]. Therefore, given this background, this work focuses on a digital stress reduction intervention as an upstream preventive measure for NCDs.

However, disparities in health care access also manifest in the “digital health divide” [8,11], where low-SES groups often lack access to preventive digital measures. While digital health interventions (DHIs) have been shown to positively influence proximal health outcomes such as patient engagement, self-awareness, and self-efficacy [12-15], they also contribute to improvements in distal health outcomes such as reduced stress [16,17], symptom reduction in depression or anxiety [18], or improved quality of life [19]. Moreover, DHIs offer several practical advantages such as overcoming geographical and temporal barriers [20], delivering engaging multimedia content [21-23], and enabling real-time data collection and biofeedback [24,25]. However, despite these benefits, socioeconomic disparities in their use [26-28] and evaluation persist [29]. Most notably, individuals with higher educational levels are more likely to use health apps than those with a lower educational level [11], meaning that underserved populations who stand to benefit the most from the scalable, personalized care that DHIs offer [29,30] are those least likely to use them.

Today, the delivery of DHIs is often skewed toward channels predominantly used by higher-SES individuals, such as workplace wellness initiatives and exclusive medical facilities, further hindering access [31]. Social media platforms, and influencers in particular, offer a promising means to reach vulnerable individuals either directly, for instance, via Facebook [32,33], or indirectly by upskilling (“influencing”) the communication of mental health content creators (“influencers”), such as on TikTok [34]. A growing body of observational studies in marketing, communication, and public health are further demonstrating that influencers can shape health-related attitudes and behaviors across a variety of contexts and populations [35-37]. Hence, working with influencers might constitute a promising strategy to promote “digital stealth health interventions” [38,39] and reach vulnerable individuals from lower social classes where they already are “healthifying their comfort zones” [40].

Beyond challenges in reaching vulnerable individuals, the design of a DHI itself plays a critical role in sustaining engagement, especially among vulnerable individuals. Low-SES individuals often face barriers such as limited digital literacy and health literacy or competing priorities, making it essential for the DHI to be engaging, relatable, and accessible, as well as relevant to their distinct needs, values, and resources [41].

Taken together, while DHIs hold immense potential, their successful implementation and impact depend on addressing the persistent challenges of reaching and engaging individuals of a low SES. Accordingly, our primary research questions (RQs) are as follows:

1. Can we leverage influencers to reach vulnerable individuals to engage with a DHI, and how?
2. Can we improve vulnerable individuals’ engagement with milieu-specific DHI design, and how?

### Understanding Social Milieux to Reach and Engage Vulnerable Individuals

To effectively engage low-SES individuals with DHIs, it is important to recognize that vulnerable populations are not a homogeneous group but, rather, encompass a range of value orientations and social contexts each representing specific subpopulations. Indeed, while general economic aspects of SES remain a critical factor in understanding health disparities, recent sociological theories have shifted toward more nuanced frameworks that also account for individuals’ value orientations and lifestyle preferences. The SINUS Institute’s social milieu model, a widely used segmentation framework [42], captures these differences by categorizing populations based on both their social status (educational level, income, and occupational status) and basic values (cultural norms, aspirations, and worldviews) [43].

**Figure 1:**
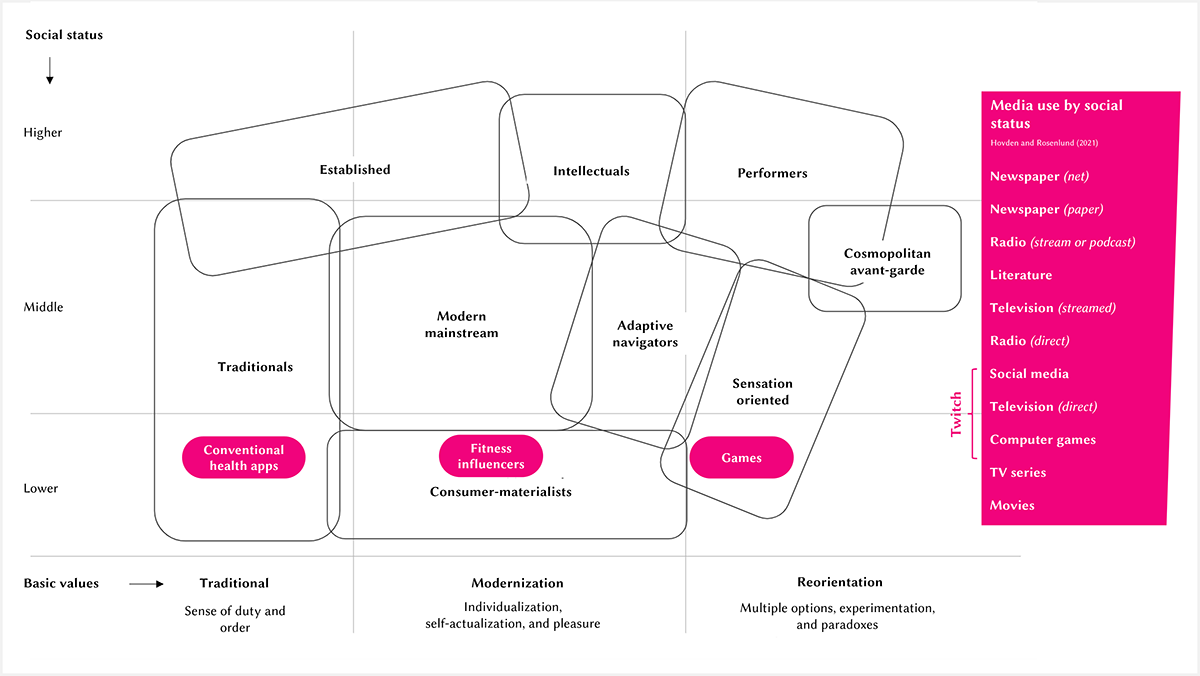
Using social milieux and media use preferences to frame digital health interventions.

Figure 1 [43,44] illustrates how these social milieux can be mapped along two axes: (1) the vertical axis representing social status, ranging from low to high; and (2) the horizontal axis reflecting basic values, classified as traditional or conservative, modern or individualistic, and experimental or sensation oriented. These values are often shaped by the historical contexts in which individuals have lived and the experiences of different generations [42]. This model reveals that, even within lower-SES populations, there are diverse subgroups with varying lifestyle preferences and media consumption habits [44]. In this work, we focus on the 3 distinct social milieux characterizing populations of a lower social status: “traditionalists,” “consumer-materialists,” and “sensation-oriented individuals.”

Traditionalists are typically older, conservative, and oriented toward security and established social norms [43]. They are more inclined to engage with familiar and conventional media outlets such as television and classic social media [44].

Consumer-materialists find themselves in precarious living conditions, too, but aspire to the consumption standards of the middle class, seeking status and gratification through modern, individualistic experiences. They value modernization, individualism, self-actualization, and pleasure [43]. They are more likely to engage with social media, gaming, and video content as a means of personal fulfillment [44].

Sensation-oriented individuals are younger and driven by a desire for entertainment, novelty, and action. They gravitate toward immersive and fast-paced media such as computer games [44] and thrive on experiences that offer novelty and engagement, preferring highly visual and story-driven content [43].

### Reaching Vulnerable Individuals via Influencers

Low-SES individuals tend to engage more with social media platforms [45], television (direct and streaming), and computer games than with traditional media outlets such as newspapers [44] (Figure 1 [43,44]). Accordingly, social media platforms offer a promising avenue for promoting DHIs and reaching vulnerable individuals. Content creators on social media in particular have the potential to serve as influencers, leveraging their relationships with their audience to encourage engagement with DHIs [34]. In 2023, major social media platforms such as YouTube, Instagram, and TikTok reported billions of daily active users [46] consuming vast amounts of content, making them ideal channels for health communication. Research further indicates that users on these platforms are strongly influenced by content creators when making purchasing or behavioral decisions [47,48], further supporting the potential of leveraging influencers to promote DHIs effectively.

However, leveraging influencers for health promotion does not come without challenges. Studies have highlighted that the impact of influencers can vary considerably depending on the credibility of the influencer, the topic, and the audience’s media habits and literacy, with some research showing limited or even counterproductive effects among certain groups [36,49,50]. Moreover, social media platforms have been criticized as fertile grounds for misinformation and health-related disinformation, with some influencers deliberately or inadvertently spreading inaccurate or misleading information to boost engagement or viewership (“clickbaiting”) [51,52].

Despite these limitations, influencer-based strategies remain underexplored in the context of embedding DHIs for vulnerable groups into their “comfort zones.” To our knowledge, there is no prior work that has assessed the effectiveness of influencer marketing when their content genre and audience values are systematically and conceptually aligned with the design of the DHI itself, particularly in the context of socioeconomically diverse social milieux.

We hypothesize that, by further matching the focal genre of an influencer’s content to the values of the targeted social milieux, DHIs can be positioned within the comfort zones of distinct social milieux. For example, influencers who produce real-life, lifestyle-oriented content may be more effective in promoting DHIs that are framed like conventional health apps to traditionalists, whereas influencers in the gaming community may be better suited for reaching sensation-oriented individuals to engage with DHIs packaged as a smartphone game. Taken together, we hypothesize that promoting a DHI through influencers whose content genre aligns with the basic values of a target social milieu will increase reach among vulnerable individuals (hypothesis 1).

### Engaging Vulnerable Individuals Using Milieu-Specific DHIs

**Figure 2:**
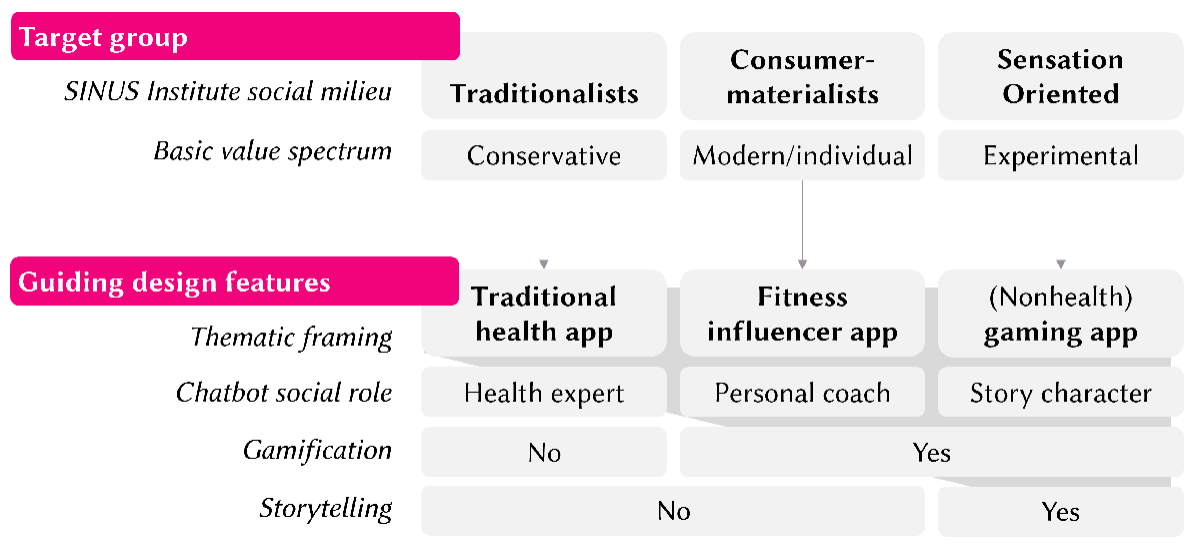
Overview of the guiding design features to target digital health interventions to the SINUS Institute social milieux.

Given our focus on DHIs in prevention health care settings and given the specific basic values (ie, conservative, modern or individual, and experimental) of the 3 target social milieux (ie, traditionalists, consumer-materialists, and sensation oriented individuals), we were eager to develop design guidelines (Figure 2) that would allow us to manipulate a DHI’s design along the spectrum of the social milieux’s basic values with regard to (1) the thematic framing of the app, (2) the social role embodied by the chatbot delivering the DHI, (3) the integration of gamification, and (4) storytelling and narrative elements—classic features in human-computer interaction and technology adoption research to enhance user engagement [53-55].

First, thematic framing involves embedding the intervention within a context that is similar to the target milieux’s values. The thematic coherence of a DHI can serve as a cognitive *anchor*, helping reduce distance and making it easier for users to relate to the intervention [56,57]. Such a thematic framing strategy might help a DHI feel less like a disruptive experience [58] and more like a natural extension of users’ daily media consumption habits [59]. We reasoned that, for instance, a DHI resembling a conventional health app might appeal to more conservative, security-oriented traditionalist individuals by evoking themes of familiar, predictable contents. In contrast, framing a DHI as a fitness influencer app focusing on self-expression may attract users who prioritize self-fulfillment. Similarly, a DHI designed like a game without any salient or obvious links to health might appeal to sensation-oriented individuals.

Second, social role theory suggests that the role played by a chatbot can influence user engagement [60]. When chatbots are designed to embody particular social roles (eg, health expert, coach, or relatable peer), they can activate social scripts and expectations aligned with the users’ value orientations [60]. In line with the thematic framing, traditional users may prefer a paternalistic expert that conveys authority and provides clear instructions (ie, a chatbot that offers step-by-step health advice, such as “Please follow the prescribed actions carefully to achieve the desired outcomes”), whereas individualistic users might respond better to a competitive coaching figure that motivates self-improvement (eg, “You’re on the right track! How about beating your last record?”). Sensation-seeking users, on the other hand, might engage more deeply with a story-based companion that integrates gamified elements (ie, “You’ve just unlocked a new level! Keep going to help the character complete the adventure!”).

Third, in line with the thematic framing, including gamification elements in DHIs for the consumer-materialists and sensation-oriented individuals has the potential to increase the experiential value for participants [61,62], potentially leading to higher commitment and adherence [53,63-65]. Gamification elements encompass quests and challenges or points, badges, and leaderboards in applications and for purposes beyond mere entertainment, such as education and health promotion [66].

Similarly, storytelling and narrative elements function as a mechanism for creating immersive and emotionally engaging experiences, potentially especially interesting for sensation-oriented low-SES individuals. Narratives have the power to draw users into an intervention by providing a framework for understanding complex concepts, building emotional connections, and enhancing the relatability of the content. Storytelling also allows for the creation of identifiable characters and scenarios, which can serve as points of reference for users to project their own experiences, thereby fostering a deeper personal connection to the intervention [54,55]. Each of these mechanisms—gamification, storytelling, and social roles—can be strategically used to optimize the thematic anchoring of a DHI and engagement by aligning the intervention’s design with the needs of specific low-SES social milieux. Taken together, we hypothesize that tailoring DHI design elements—specifically, thematic framing, social role, gamification, and storytelling—to reflect the value orientation of specific social milieux will enhance user engagement (hypothesis 2).

## Methods

### Study Design

To answer our RQs, our study used a cross-sectional, quasi-experimental field study design to investigate the impact of differently designed versions of a health-promoting app that were promoted via influencers on the video streaming platform Twitch on audience reach and user engagement with the app.

### Procedure

To describe the study procedure, we need to, first, differentiate between three different parties involved in the study—(1) the study team, (2) the influencers, and (3) the end users targeted by our DHI (ie, our study participants=low-SES individuals)—and, second, divide the study into four parts—(1) development of the different app versions and study setup (refer to the Development of Study App Versions and Technical Implementation section), (2) recruitment and selection of the Twitch influencers (refer to the Recruitment and Selection of Twitch Streamers section), (3) reaching study participants through the streamers’ viewers (refer to the Recruitment of Study Participants via Influencers section), and (4) engagement with the study app by the target study participants (refer to the User Interactions With the Study App section).

#### Development of Study App Versions and Technical Implementation

This study used a fractional factorial design to investigate the effects of a differently designed smartphone-based and chatbot-delivered DHI featuring an evidence-based slow-paced breathing training [64,67] on user reach and engagement (refer to hypothesis 2).

#### Development of the Milieu-Specific DHIs as Experimental Stimuli

### Overview of App Versions

Overall, we modeled 3 distinct versions of the DHI along the 3 low–social status social milieux’s guiding design principles outlined in Figure 1 [43,44] with regard to the following app elements: (1) graphical design, (2) the featured chatbot and its social role and conversational style, and (3) the graphical design of the guided slow-paced breathing training.

StressLess aimed to mimic state-of-the-art health-related apps such as Calm [68] or Headspace [69] and address the traditionalist milieu, who have conservative values and appreciate status quo–oriented apps; the focus of the app was primarily on the health and prevention aspect, and the design was moderate and plain. The breathing training was traditional without gamified components, which leads to rather low experiential or hedonic value.

Breeze aimed to address the value of pleasure and individualization of the consumer-materialist milieux. Breeze builds on the base of StressLess by maintaining a focus on the health and prevention aspect but imitates modern fitness and lifestyle influencers. The breathing training has gamified elements that increase the experiential value.

TragicKingdom was intended to address the hedonistic values of the sensation-oriented social milieu by implementing a storytelling approach with characters from the action role-playing game genre that leads the user through an adventure with the app character. The focus shifts from health and prevention to the storytelling aspect and the hedonistic and entertaining features of the app. The breathing training is also gamified and is integrated as a direct part of the story, which offers a high experiential value.

**Figure 3:**
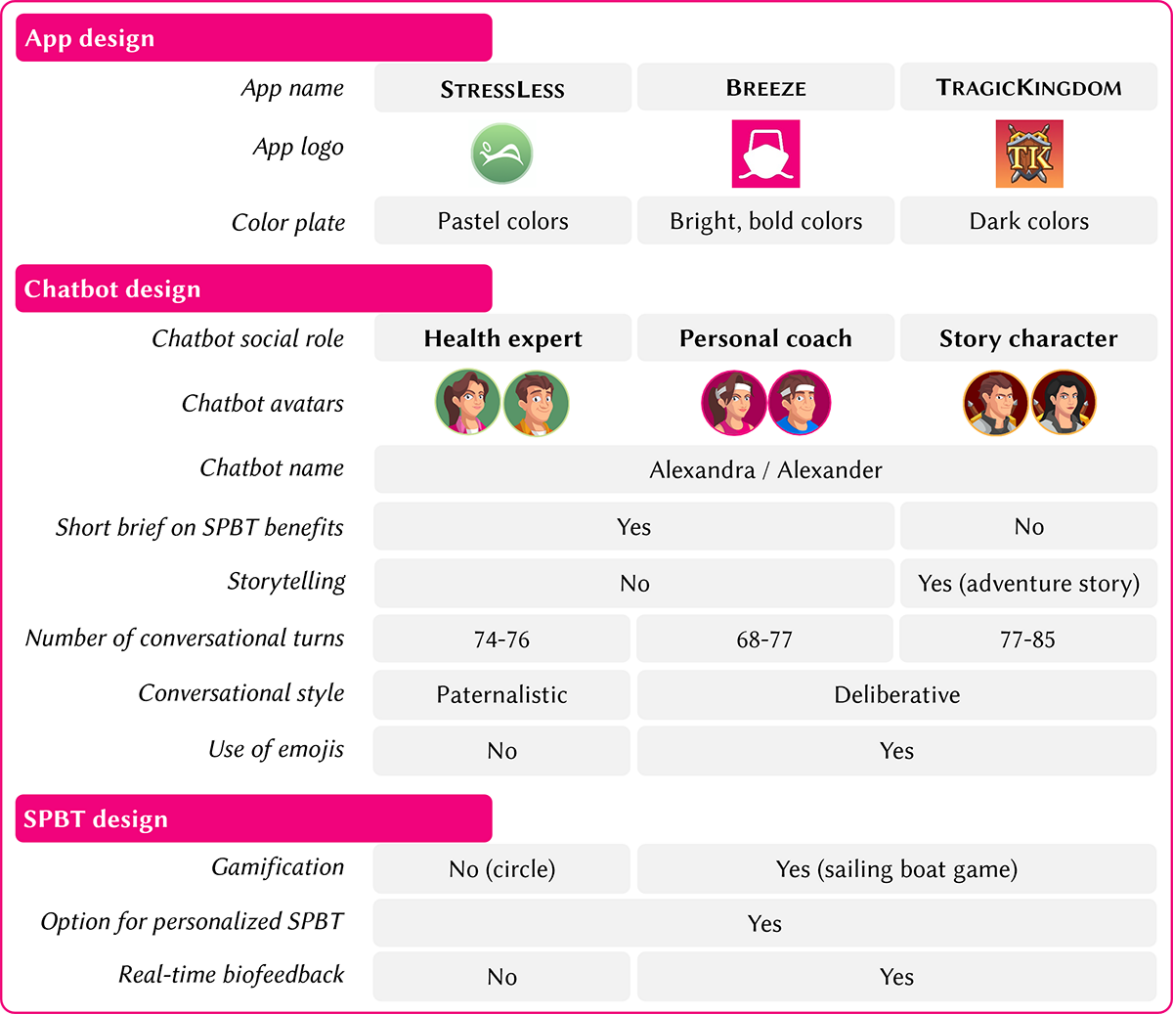
Overview of the specific design differences among the 3 digital health intervention versions. SPBT: slow-paced breathing training.

Figure 3 provides an overview of the differences among the 3 app versions. Details are outlined in the following sections.

### App Design

**Figure 4:**
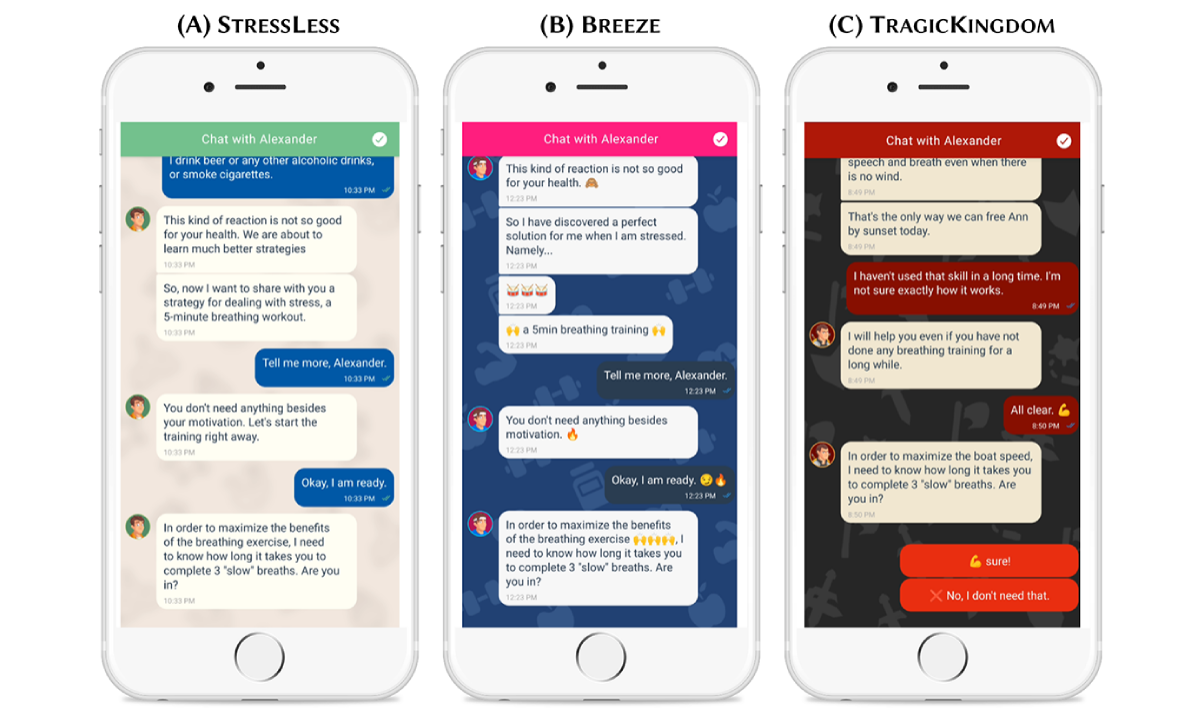
Screenshots of the different digital health intervention versions depicting the different app designs, conversational styles of the chatbots, and storytelling elements.

The 3 DHI versions purposefully differed in terms of the overall look and feel of the app with regard to the name, logo, and color palette. While StressLess was toned down with calming and relaxing pastel colors; a rounded logo; and a conventional, reassuring name, Breeze was designed with bright, refreshing colors; a squared logo featuring the outline of a sailing boat as a reference to the storytelling features; and a modern, fun name. To highlight the storytelling and gamification features, TragicKingdom was designed using dark and mystical colors, a logo featuring a fantasy game–inspired emblem, and an immersive name. These themes were implemented thoroughly throughout all app and design components, including, for instance, the welcome screen, background screens, and chat message, but also in the design of the chatbot avatars and the breathing exercises. Figures 4A-4C illustratively show the similarities and differences in terms of design, the chatbots’ social roles and conversational style, and storyline based on a user interaction with the chatbots in the different app versions shortly before the breathing training starts. More screenshots of each app version are provided in Multimedia Appendix 1 (section A3).

### Chatbot Design

The chatbots in the different app versions differed with regard to their avatar; the overarching storyline; the social role they represented; and their use of distinct interaction styles, wordings, and emojis. First, each version relied on a unique storyline—StressLess and Breeze featured a short introduction on the benefits of stress management (“patient education”), whereas TragicKingdom avoided any explicit educational contents and featured an adventure story instead. Second and third, the chatbots in the distinct app versions represented distinct social roles and used different interaction and linguistic styles accordingly. The chatbot character in StressLess was represented by a more distant “health expert” social role [60] that incorporated a paternalistic interaction style guiding the user through the intervention with a “top-down” approach, whereas the character in Breeze was framed as personal coach using a more deliberative interaction style characterized by joint decision-making approaches and the chatbot in TragicKingdom resembled a story character role. In all app versions, during onboarding, participants were allowed to choose between a feminine avatar called Alexandra or a male avatar called Alexander as previous chatbot studies have shown positive effects of letting individuals customize their chatbots [60]. Fourth, emojis were used by the chatbots in Breeze and TragicKingdom to emphasize text and express feelings. To further amplify the story in TragicKingdom, some photographs were also shared via the chat (eg, a picture of the character who needed to be rescued by the user and the map of the island).

### Slow-Paced Breathing Training Exercise Design

Preventing NCDs involves primary, secondary, and tertiary measures, with this study focusing on primary prevention and educating vulnerable individuals on maintaining a healthy lifestyle [70,71]. Psychological stress is a significant factor in the development of many NCDs such as hypertension, diabetes, and cardiovascular disease [3,4,72], triggering harmful mechanisms such as the fight-or-flight response when prolonged [5,73,74]. Research shows that stress can alter biological processes relevant to diseases, impacting health status [3].

To mitigate stress, slow-paced breathing training has proven effective in improving psychological and physiological well-being [75]; reducing the risks of mental and chronic physical health conditions, including depression [76], anxiety [77], stress [78], chronic obstructive pulmonary disease [79], type 2 diabetes mellitus [80], chronic pain [81], and hypertension [82]; and managing acute and chronic stress [75,83]. Digital, biofeedback-enhanced slow-paced breathing training using biosignals to guide users through the exercise has shown particular promise in stress reduction and user engagement, especially for less motivated or vulnerable individuals [67]. However, designing effective biofeedback breathing training with gamified components remains challenging [64].

**Figure 5:**
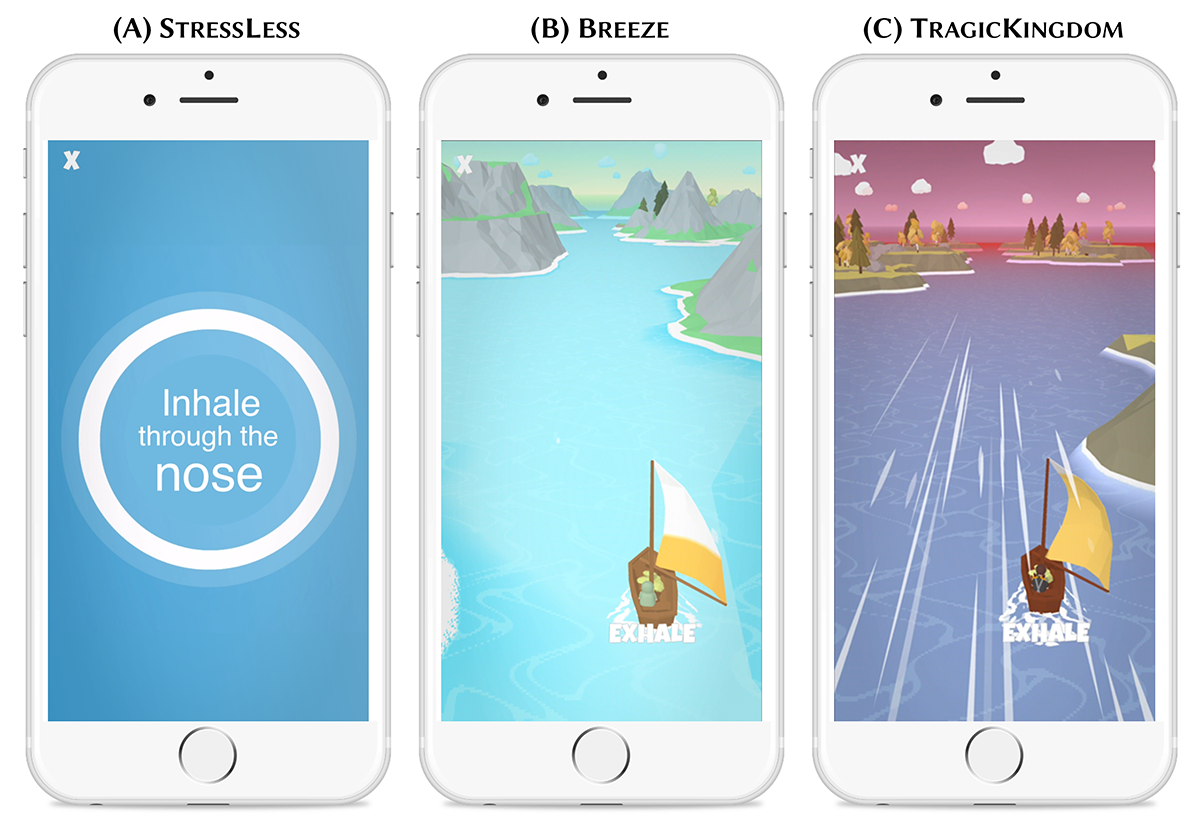
Slow-paced breathing training design in (A) StressLess, (B) Breeze, and (C) TragicKingdom.

The slow-paced breathing training was adapted in terms of color, functionalities, and design in line with the overarching storylines and design themes of the app versions. The training in StressLess mimicked state-of-the art meditation and breathing exercise apps through a circle that expanded and shrank with the inhalation and exhalation with no further gamified components or biofeedback (Figure 5A). Moreover, the design was kept simple in a calming blue color scheme.

Alternatively, Breeze and TragicKingdom included a gamified and lavishly designed slow-paced breathing training with real-time biofeedback (Figures 5B and 5C). The focal design element of the training was a sailing boat that accelerated through the breathing pattern of the user and reached new landscapes; this element provided a gamified component and increased motivation to continue. On the basis of previous research, the landscapes included gardens, trees, and green spaces to set a natural environment and foster a calming and meditative effect on the user. The game used the sailing boat and the wind as dynamic objects within the static environment. During the inhalation phase, the wind moved against the direction of the boat, and during the exhalation phase, the wind moved toward the boat to inflate the sails. These movements illustrated the breathing patterns and guided the user through the breathing cycles. The wind strength symbolizes biofeedback, and therefore, the quality of the breaths was illustrated by the movement of the sail, the boat’s speed, the size of the waves, and the increasing number and speed of particles [64,67,84]. Previous studies have proven the functionality and applicability of the aforementioned breathing training. For example, gamified training has a higher perceived enjoyment from participants than a standard breathing exercise, such as using a circle (StressLess). Studies have also reported that gamified biofeedback increases the experimental values and fosters engagement [64,67]. These design distinctions led to significant differences in terms of experiential value, design, and biofeedback.

#### Technical Implementation

The study prototype apps were developed using the open-source (Apache 2.0 license) software platform MobileCoach (Centre for Digital Health Interventions at University of Zurich, ETH Zurich, and University of St. Gallen) [85-87]. This platform has been used for various other digital interventions, for example, to prevent depression and type 2 diabetes through a holistic lifestyle intervention [88]; reduce distress in patients with cancer [89] or students [90]; follow a balanced diet [91]; change a specific personality trait [92]; reduce headaches [93]; and improve hypertension management [94], sleep [23], or type 1 diabetes management [95].

Interventions based on MobileCoach are delivered via SMS text message and rule-based chatbots. Accordingly, the chatbots in our study app versions offered predefined answer options that encompassed multiple-choice alternatives but also allowed for free-text input (eg, when asking for the nickname of the participant). Within the predefined script, answer choices lead to different conversation pathways, imitating human interactions and giving the participants the feeling of personalized and adaptive conversational turns. Figures 4A-4C illustrate example dialogues with the chatbot.

The apps could be operated on Android as well as iOS operating systems. The system ensures data privacy and security through password-protected access to the MobileCoach designer and server; in-app encryption; and SSL encryption for data transfers between the mobile apps, MobileCoach designer, and MobileCoach server [85-87].

The breathing training was adapted and developed based on and implemented using the Unity game engine (version 2019.1.8; Unity Technologies) [67]. The 3D models were designed using the modeling software Blender (Blender Foundation) [64,67].

The 3 apps were developed over a period of approximately 8 months with several internal testing rounds by the coauthors.

#### Recruitment and Selection of Twitch Streamers

##### Overview

Twitch [96] is a social video streaming platform for computer games and, therefore, a promising way to promote DHIs and reach vulnerable individuals from lower-income classes. In the United States, roughly half (53%) of teenagers living in households with an annual income of <US $30,000 play video games at least daily compared to 42% living in households with an annual income of US $30,000 to US $74,999 and 39% living in households with an annual income of ≥US $75,000 [97].

Research further indicates that Twitch users are strongly influenced by streamers when making purchasing decisions [48], also making content creators on Twitch (also referred to as “influencers” or “streamers”) influential marketers with great potential to prompt and convince their audience to engage with DHIs. In 2023, Twitch reported having >35 million daily active users [98] and 21.4 billion hours of Twitch consumed [99].

##### Milieu-Informed Influencer Genre Promotion

**Figure 6:**
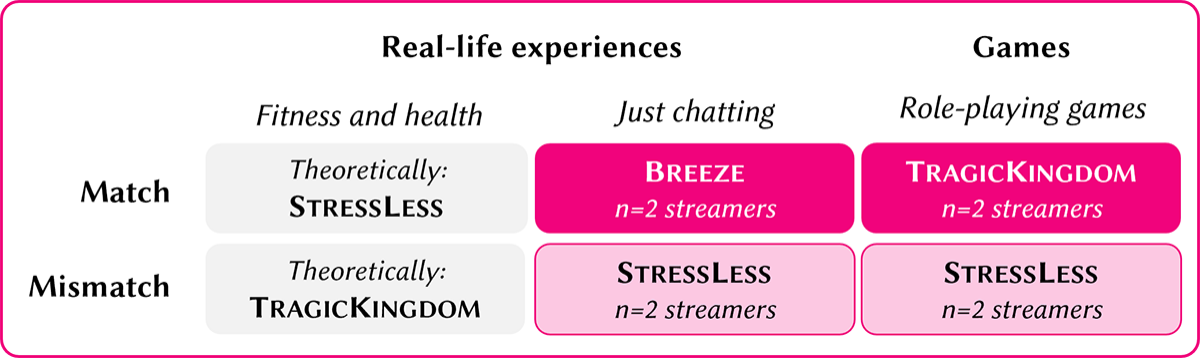
Overview of influencer genre matching with the digital health interventions.

To better understand how the design of a DHI could be tailored to the values of different social milieux, this study leveraged the concept of milieu-informed influencer promotion introduced previously (refer to the Introduction section). By aligning the genre of a Twitch streamer (influencer) with the design of a DHI, we hypothesized that the genre that most closely matched the target milieu’s values would enhance reach and user engagement (refer to hypothesis 1). This strategy was operationalized as either a match or a mismatch between the streamer’s most frequently streamed category (genre) and the thematic framing of the DHI (Figures 2 and 6). The match or mismatch approach assumed that DHIs framed and promoted in a genre familiar to the target audience would reduce psychological barriers and enhance user resonance with the intervention (refer to hypothesis 1).

Twitch provides a variety of streaming categories, such as *games*, *real-life experiences* (known as *IRL*), *e-sports*, *music*, and *creative content*. Each of these categories tends to attract users with specific preferences and interests, which align with different social milieux. Thus, we expected (1) real-life experiences (IRL) genres, which include *just chatting* and *fitness and health* (among others), to resonate with individuals from milieux that value personal experiences, lifestyle content, and real-world interactions; and (2) game genres, which encompass role-playing games (eg, The Witcher) or simulation games (eg, The Sims), to appeal to users who enjoy immersive, fast-paced, and entertainment-driven content.

Given these distinctions, this study matched or mismatched the influencer’s primary genre with the thematic framing of the DHIs to target specific social milieux: the influencer’s genre aligned with the thematic design of the DHI (eg, a gaming influencer promoting a DHI framed as a game for sensation-oriented individuals; match) or the influencer’s genre did not align with the thematic framing of the DHI (eg, a gaming influencer promoting a traditional health app for conservative individuals; mismatch)

In the TragicKingdom match condition, this DHI, framed as a gaming app with storytelling and gamification elements, was promoted by influencers from the *games* genre. The match between the sensation-oriented milieu’s values (thrill seeking, entertainment focused, and anti–conventional attitudes) and the gaming genre was intended to increase reach and engagement through a narrative-driven, immersive experience.

In the Breeze match condition, the Breeze DHI, framed as a personal influencer app, was promoted by influencers who primarily streamed in the *just chatting* subcategory of the *real-life experiences* genre. This alignment was designed to resonate with the consumer-materialists who value self-expression, status, and personal fulfillment, assuming that the influencer app framing aligns with this social milieu’s preference for modern, individualistic values.

In the StressLess mismatch condition, StressLess was framed as a traditional health app, which should have been promoted by *fitness and health* streamers according to the milieu-informed influencer genre promotion strategy. However, *fitness and health* is a less frequented genre on Twitch, and it proved unfeasible to recruit influencers from this category. Therefore, we deemed StressLess to serve as a mismatch and control condition promoted by influencers from both the *real-life experiences* and *games* genres, thus allowing us to test whether promoting a conservative, prevention-focused app to audiences accustomed to entertainment-heavy, experience-driven content would affect reach and user engagement.

Participants and streamers were not informed about the match and mismatch conditions.

##### Recruiting the Influencers via an Influencer Marketing Agency

For the actual identification, selection, and recruitment of potential Twitch streamers, a professional agency was involved. The agency had experience with advertisements on Twitch as well as access to a large pool of potential streamers. In the first step, the following requirements for the recruitment of the Twitch streamers were agreed upon with the agency: (1) streamers were selected based on their most frequently streamed category (or genre) to induce either a match or a mismatch between the promoted DHI version and the targeted social milieu and the streamers’ most frequently streamed genre (Figure 6); and streamers needed to (2) have between 500 and 30,000 followers (due to financial constraints) and (2) be German speakers and advertise the study and app in German in their stream. Furthermore, we aimed to recruit 2 streamers per app and genre to reduce variation in downloads attributable to the individual performance of each streamer. As it was not possible to recruit influencers from the health and fitness *IRL* subcategory, we eventually only included 8 streamers.

In a second step, the agency developed a proposal with 27 preselected eligible Twitch streamers, 10 (37%) of whom were highlighted as particularly well fit by the agency. This short list from the agency included the name and approximate age of the streamer, streaming category, number of followers, and basic research on conspicuous behavior on the internet.

In a third step, the research team, in collaboration with the agency, selected the 8 most suitable streamers. On the basis of this decision, the agency approached the Twitch streamers. If a chosen streamer did not confirm their interest in executing the advertisements, the agency approached another streamer from the proposal in consultation with the research team. The streamers were bound by contracts established by the agency, which included a requirement of a minimum number of downloads and a request for poststream data.

As the focus of this study was on the comparison of the 3 app versions, we recruited 2 streamers per study condition to reduce biases of the individual recruiting of each streamer and their followers (Figure 6). The communication with the streamers was closely coordinated with the research team. More insights regarding the recruitment process of the streamers are disclosed in Multimedia Appendix 1 (section B1).

#### Recruitment of Study Participants via Influencers

Between January 2022 and April 2022, the streamers performed the advertisement live on their streams. All MobileCoach and LimeSurvey (LimeSurvey GmbH) data were downloaded and saved in May 2022. Streamers were free to choose the timing and frequency of the advertisements as long as the agreed-upon number of downloads was reached within the predefined period. To ensure consistency, each streamer received guidelines from the agency that were developed by the research team; these guidelines included the leading principles, important information, and a proposed script for the advertisement (Multimedia Appendix 1, sections B1.1 and B1.2). While each streamer retained the freedom to perform the advertisement according to their personal preferences and (conversational) styles, the following guidelines required that specific aspects be included in each advertisement stream: (1) banners with a QR code directly linked to the apps were preferred to be displayed during the entire stream. The banner was designed and provided by the agency. Examples of banners are shown in Multimedia Appendix 1 (section B1.3). (2) Promotion videos were required to be shown at the beginning of, during, or at the end of the stream. They provided a short overview of the different parts of the app to manage the expectations of the potential participants. Each video was approximately 50 seconds long and was labeled as “advertisement.” The links to the videos can be found in Multimedia Appendix 1 (section B1.4). The promotional videos again repeated the design themes of the corresponding app version. We even adapted the background music on the video to fit the corresponding design theme. (3) Directly spoken advertisements were used, in which streamers were asked to speak directly to the audience and motivate them to install the app during the stream. (4) The app was demonstrated (if possible); streamers should show their phones with the app installed into the camera to signal the credibility of the app and increase trustworthiness. (5) Additional information was shared (if possible); the streamers were instructed to share further information to motivate the user to download the app, such as mentioning that it is a new beta app, that it is the first chance to take part in such a study, that there are no costs to participate, that the users’ feedback is important for the next development steps, or that there is an association with ETH Zürich. (6) Lottery and prize amounts were deliberately *not* to be mentioned in the advertisement to avoid monetary incentive effects.

Furthermore, the overarching differences among the 3 apps were also respected in the guidelines. Streamers advertising StressLess were advised to advertise the breathing training only. Streamers advertising Breeze and TragicKingdom were asked to explicitly advertise the sailing ship theme in the breathing training (Breeze and TragicKingdom) or the storyline (only TragicKingdom). The differences among the scripts are highlighted in Multimedia Appendix 1 (section A1).

**Figure 7:**
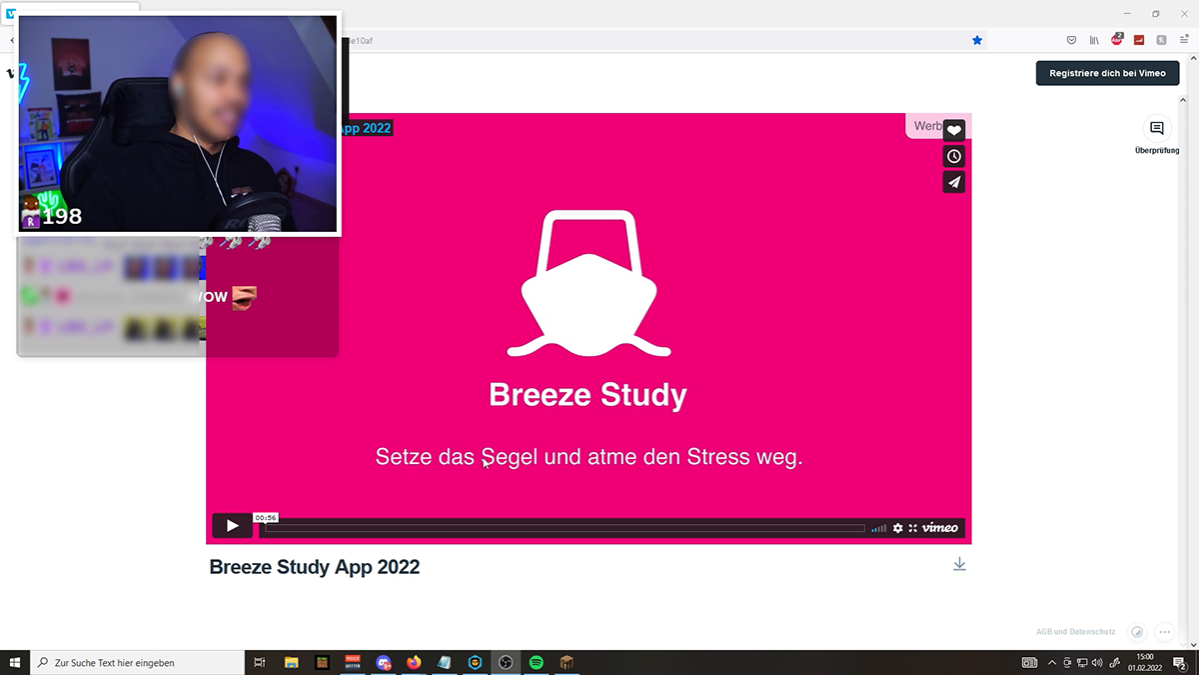
Example screenshot of an advertisement during a stream. The influencer shows the promotion video of the Breeze study app by sharing his screen with the live viewers.

To measure downloads per streamer individually, each streamer received and shared a personalized deep link shared as a link in the chat and via a QR code on a banner during the stream. Figure 7 illustrates an example of a stream (more examples can be found in Multimedia Appendix 1, section C2).

#### User Interactions With the Study App

From an end user perspective, study participants only learned about the study app while watching the stream of a Twitch streamer. The study procedure and app flow from an end user’s perspective as well as end user–specific inclusion and exclusion criteria will be described in the following sections.

#### Study Procedure From an End User Perspective

**Figure 8:**
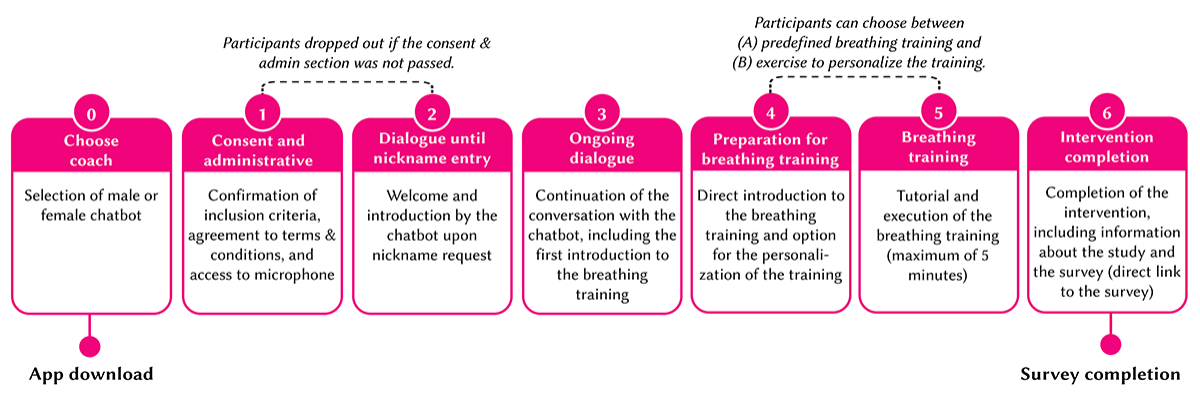
Process and milestones of the interventions.

Overall, the study procedure from an end user perspective was similar for participants exposed to StressLess, Breeze, or TragicKingdom (Figure 8). Each step also represents a predefined milestone and time stamp of the intervention later used for analysis.

Participants downloaded the app via an advertisement link in the stream chat or a QR code and then installed and used the app on their personal mobile devices. Upon first opening the app, participants selected their preferred chatbot (milestone 0) and completed a consent dialogue in which they provided permissions for app functionality. Participants who did not pass this section dropped out of the study (milestone 1). Participants were then welcomed and introduced to the study until a point in which they needed to provide a nickname (milestone 2). In the TragicKingdom app, participants were introduced to the storyline, whereas StressLess and Breeze provided information and questions about stress (milestone 3). The chatbot guided participants through preparing for a slow-paced breathing exercise (milestone 4) where they could choose between predefined (fixed number of 5 breaths per minute) or personalized (calculated based on individual performance) pacing. A tutorial provided instructions on posture, breathing techniques, and the exercise duration (milestone 5). The breathing exercise itself lasted a maximum of 5 minutes, after which the chatbot introduced a final survey and offered the chance to participate in a lottery as motivation (milestone 6). After completing the survey, participants were encouraged to delete the app. Detailed information on each step and milestone is provided in Multimedia Appendix 1 (section A2).

#### Inclusion Criteria

People of any sex (male, female, or other) were eligible to participate in the study if they were aged >18 years and fluent in German. People who were in a location where wearing a face mask (eg, on the train or in public inside locations) was mandatory were not eligible to participate in this study as the breathing exercise could not be conducted while wearing a face mask. People who had cardiovascular diseases, severe respiratory diseases, or mental illnesses were also not eligible to participate in this study. The inclusion criteria were queried at the beginning of the intervention in the chatbot conversation and were double-checked in the survey at the end of the intervention. Participant data were excluded and deleted retrospectively if one or more criteria were not met.

#### Measurements

Different measures were implemented for recruitment success (the reach), to evaluate the engagement with and acceptance of the interventions, and to assess the distribution of individuals in terms of SES.

#### Reach Metrics

To assess the recruitment success (RQ 1), the reach of each streamer and allocated intervention was measured. The reach was calculated as the ratio of approached participants (viewers) on Twitch (ie, average viewers during each advertisement stream) to the number of downloads per advertisement stream and streamer.

Reach = Number of downloads / Average viewers

The data on average viewers during the stream were retrieved from stream summaries generated by Twitch that were shared with us by the streamers themselves and cross-checked with archival data from TwitchTracker (in July 2024)*,* which uses Twitch’s public application programming interface to collect up-to-date and historical viewing data per streamer (Multimedia Appendix 1, section C3 depicts a screenshot of the information provided by TwitchTracker for one stream)*.*

Only downloads that could be clearly allocated (due to deep link, manual request, or timing) to a streamer and a specific advertisement stream were considered in the calculations. Average reach ratios were calculated both per streamer (n=8) and per app version (n=3), and the overall average reach ratio was calculated as the average of all 8 streamers’ or app versions’ average reach ratios.

#### Engagement Metrics

The engagement with the intervention was assessed via the number of conversational turns and milestones completed in the interventions (the predefined milestones are described in Figure 8 and further detailed in Multimedia Appendix 1, section A2). The maximum number of conversational turns varied among the different conditions, and this factor was considered when calculating the engagement. Furthermore, the intervention completion rate was assessed by dividing the number of participants who finished the interventions by the number of participants who downloaded the apps. The calculations were all based on the app use data collected by MobileCoach. The milestones were implemented as time stamps on MobileCoach.

The engagement with the breathing training was measured via the amount of time spent on the breathing training. The maximum time was 5 minutes (300 seconds). The engagement rate was calculated by dividing the number of minutes spent on the breathing training by the maximum number of minutes. The time spent on the breathing training was measured through time stamps implemented in MobileCoach.

#### Success of the Breathing Training

Before and after the breathing training, the current emotional state of the participants was evaluated using 1 question from the Multidimensional Mood State Questionnaire [100]. The following question—“How do you feel right now?”—was translated from the German version of the questionnaire. The answers were labeled as 6 categories: 1=definitely not good, 2=not good, 3=rather not good, 4=rather good, 5=good, and 6=extremely good [100,101]. In addition, MobileCoach registered the time that the participant spent on the breathing training (maximum of 5 minutes). Furthermore, the distance traveled with the sailing boat (Breeze and TragicKingdom) was measured as an indication for the quality of the execution of the breathing pattern (ie, correct and clean breaths led to a greater distance).

#### SES Measurement

To evaluate the demographics and SES of the participants of the intervention, information about sex, age, country of residence, general health status, employment status, educational level, occupation status, and monthly income were gathered in the survey at the end of the intervention. All survey measures can be found in Multimedia Appendix 1 (section D1).

#### User Perceptions of the App

To better understand users’ engagement with the app as well as with the breathing training, users’ perceptions and use intentions of the app and the breathing exercise were assessed in the survey at the end of the interventions. Perceived ease of use, enjoyment, and use intention were queried using single-item measures on a 5-point Likert scale ranging from strongly disagree (1) to strongly agree (5) [102,103]. Finally, the survey collected qualitative feedback on positive and negative aspects of the interventions.

#### Statistical Analysis

##### Overview

The descriptive statistics, including means and SDs for baseline characteristics as well as recruitment and retention, acceptability, and engagement measures, were calculated using Microsoft Excel (Microsoft Corp) and the statistical software R (version 3.5.2; R Foundation for Statistical Computing).

To assess significant differences in reach ratios across different streamers and app versions, ANOVAs (or Kruskal-Wallis tests when assumptions for parametric tests were violated) were conducted.

To examine differences between conditions (eg, intervention groups) in terms of all milestones and intervention completion, chi-square tests were used. Further chi-square tests were also used to assess the impact of the participants’ operating systems (iOS vs Android) on completing the breathing training, as well as the relationship between different streamer IDs and the completion of the breathing training. ANOVAs (or Kruskal-Wallis tests when assumptions for parametric tests were violated) were conducted to assess significant differences in the number of conversational turns and total time elapsed (in minutes) per intervention.

Finally, to classify the participants’ SES**,** a multidimensional SES index was used. This index was calculated based on point scores assigned to the 3 equally weighted dimensions of education, occupational status, and income. The points were manually assigned by 2 coauthors (SH and MN) according to the most recent scheme provided by Lampert et al [104]. The full scoring process is detailed in Multimedia Appendix 1 (section E1).

##### Power

This study was exploratory by nature as a new recruitment strategy was implemented. To the authors’ knowledge, this study is the first to recruit participants via the Twitch platform for a health-related intervention. This study is also one of the first to promote prevention content (1) through lay individuals rather than health care–related professionals and (2) without explicit health-related prompts. Thus, a power analysis to determine the sample size a priori was not conducted. However, according to heuristics in usability engineering, we aimed to recruit at least 20 participants per condition [105]. To safeguard that number and better assess trends regarding the SES distribution of individuals who reacted to the study advertisements, the streamers were instructed to recruit at least 30 participants each. In summary, this study aimed to recruit 60 participants per condition and the associated comparison condition (240 participants in total; Figure 6).

#### Ethical Considerations

Ethics approval for this study was granted by the ETH Zürich (Swiss Federal Institute of Technology) Ethics Commission (EK-2021-N-66). The streamers were financially compensated at the market rate (between €400 and €500 [US $471.44-$589.31] per streamer), and participants were entered into a draw for a CHF 50 (US $63.13) or €50 (US $58.93) prize.

## Results

### Twitch Streamers: Characteristics and Notes

The agency reported that they contacted approximately 80 to 90 streamers before recruiting the final 8 employed in the study. The streamers’ follower numbers ranged from 360 to 44,225 (based on archival TwitchTracker data from February 20, 2022). However, the number of followers does not necessarily directly reflect a streamer’s average live viewer numbers and the engagement of the community [106].

The included Twitch streamers advertised the study between February 2022 and April 2022. The period was increased from the original plan due to delays and cancellations from the streamers.

The average viewers during an advertisement stream ranged from 16 to 3700 (mean 506.7, SD 1134.3). In total, the 8 streamers performed 25 advertisement streams. Overall, we recorded 273 downloads; 172 (63%) downloads could be allocated to a streamer ID based on information recorded via the deep link data, another 30 (11%) downloads could be allocated to a streamer based on manual requests, and 18 (6.6%) could be allocated to a streamer due to timing, resulting in a final sample of 220 total downloads. A total of 9.9% (27/273) of the downloads could not be considered as they did not include the streamer ID or could not be attributed to a streamer based on timing. Another 9.5% (26/273) of the downloads were from the participating streamer themselves or were made before the streamer’s first advertisement stream and, therefore, excluded.

Overall, the streamers performed the advertisement well and in accordance with the requirements discussed in the Recruitment of Study Participants via Influencers section. All streamers directly advertised the apps at least once during a stream; showed the promotion video; displayed the banner; and showed their smartphone with the app installed, including occasionally screen sharing the app live during the stream to enable the viewers to directly experience the intervention. All streamers streamed in the categories *IRL* or *games*. In general, the interest from the streamers and the audience seemed high.

However, it should also be noted that the precise reach of each streamer could not be fully determined due to idiosyncrasies in how individual streamers further shared their content outside of the stream (eg, uploading the stream to be rewatched on YouTube and publishing static advertisements on other social media platforms). No data were available on the reach of these marketing actions. Furthermore, streamer ID 4, for instance, initially advertised the intervention only for iOS devices (but later clarified that the intervention worked for all operating systems), streamer ID 5 executed most of the advertisements early in the morning (eg, 5 AM), and streamers ID 1 and 5 demonstrated the interventions live by screen sharing them during the stream. As expected, the advertisements by each streamer furthermore differed slightly in timing, length, style, and wording. The collaboration with the agency as well as with the streamers went well. However, coordinating 8 different streamers entailed a high communication load, short-notice changes, and adaptations. Impressions of and notes on the advertisement streams of each streamer are included in Multimedia Appendix 1 (section C3).

### End Users: Total Downloads and per App Condition

Table 1 shows an overview of the number of streams; average viewers per stream; number of generated downloads; and average reach per stream, streamer, genre, and app version. In total, 220 downloads were counted, of which 92 (41.8%) were for StressLess, 66 (30%) were for Breeze, and 62 (28.2%) were for TragicKingdom.

**Table 1:** Descriptive results of the participant recruitment per streamer and condition.

App version, genre, streamer ID and gender, and stream ID					Date of stream		Viewers, mean (SD)^a^		Downloads (n=220), n (%)		Reach per stream, n (%)^b^		Reach per streamer (%), mean (SD)			Reach per genre (%), mean (SD)				Reach per app version (%), mean (SD)			
**StressLess**																							8.1 (9.3)
	**IRL^c^**																	3.6 (6.0)					
		**Streamer 3; female**												16.7 (0)									
			3-1	February 17, 2022		203		34 (15.5)		16.7													
		**Streamer 4; female**												1 (1)									
			4-1	February 3, 2022		3461		11 (5)		0.3													
			4-2	February 4, 2022		450		8 (3.6)		1.8													
			4-3	February 13, 2022		3526		2 (0.9)		0.1													
			4-4	February 15, 2022		3700		5 (2.3)		0.1													
			4-5	March 28, 2022		439		11 (5)		2.5													
	**Games**																	14.9 (9.3)					
		**Streamer 7; male**												21.9 (7.1)									
			7-1	February 5, 2022		27		4 (1.8)		14.8													
			7-2	February 6, 2022		31		9 (4.1)		29													
		**Streamer 8; female**												7.8 (4.9)									
			8-1	February 20, 2022		55		7 (3.2)		12.7													
			8-2	February 27, 2022		68		2 (0.9)		2.9													
**Breeze**																							14 (10.5)
	**IRL**																	14 (10.5)					
		**Streamer 1; female**												10.8 (7.4)									
			1-1	February 3, 2022		37		7 (3.2)		18.9													
			1-2	February 6, 2022		57		3 (1.4)		5.3													
			1-3	February 13, 2022		62		5 (2.3)		8.1													
			1-4	February 19, 2022		66		3 (1.4)		4.5													
			1-5	February 20, 2022		62		3 (1.4)		4.8													
			1-6	March 27, 2022		56		13 (5.9)		23.2													
		**Streamer 2; male**												33.3 (0)									
			2-1	February 1, 2022		96		32 (14.5)		33.3													
**TragicKingdom**																							28.4 (17.6)
	**Games**																	28.4 (17.6)					
		**Streamer 5; male**												16.3 (7.7)									
			5-1	February 2, 022		45		6 (2.7)		13.3													
			5-2	February 4, 2022		46		4 (1.8)		8.7													
			5-4	February 15, 2022		40		10 (4.5)		25													
			5-5	February 22, 2022		48		4 (1.8)		8.3													
			5-6	March 22, 2022		27		7 (3.2)		25.9													
		**Streamer 6; male**												48.5 (8.5)									
			6-1	February 5, 2022		30		11 (5)		36.7													
			6-2	February 13, 2022		16		9 (4.1)		56.3													
			6-3	April 5, 2022		19		10 (4.5)		52.6													

^a^Average viewers per stream provided by TwitchTracker.

^b^Average reach per stream: 16.2% (SD 15.5%).

^c^IRL: *real-life experiences* genre.

Average reach per stream amounted to 16.2%, ranging from 0.1% (2/3526; ie, stream ID 4-3 and 5/3700, for stream ID 4-4) to 56% (9/16; stream ID 6-3). A Kruskal-Wallis test found significant differences between app conditions (*ꭓ*^2^_2_=8.0; *P*=.02), with average reach ratios of 8.1% for streams promoting StressLess, 14% for Breeze, and 28.4% for TragicKingdom (Table 1). Pairwise comparisons revealed significant differences between StressLess and TragicKingdom (*P*=.005) but not between Breeze and TragicKingdom (*P*=.15) or between StressLess and Breeze (*P*=.23). These findings indicate that TragicKingdom outperformed StressLess and Breeze in terms of reach per stream, whereas there was no significant difference in performance between StressLess and Breeze.

The results further highlight a match or mismatch pattern between the dominant genres of the influencers’ content and the app version they promoted. The “match” condition referred to when the content genre of the influencer aligned with the thematic framing of the DHI. The match between the *games* genre and the TragicKingdom intervention resulted in the highest reach ratio of 28.5% (SD 17.6%). In contrast, StressLess, which represented a mismatch in this genre, achieved a significantly lower reach ratio of 14.9% (SD 9.3%). On the other hand, in the *IRL* genre, the reach ratio for Breeze, which was a match for this genre, amounted to 14% (SD 10.5%), whereas StressLess, a mismatch, resulted in only 8.1% (SD 9.3%) reach. This pattern suggests that aligning the genre of the influencer with the thematic design and framing of the intervention can enhance user engagement, confirming hypothesis 1.

### Users: Engagement With the Study App and the Breathing Training

#### Interaction Data

Table 2 shows interaction with and engagement metrics regarding the app. In total, 72.7% (160/220) of all users had an iPhone (iOS operating system), with significant differences between TragicKingdom (50/62, 81%), StressLess (71/92, 77%), and Breeze (39/66, 59%; *ꭓ*^2^_4_=13.0, *P*=.002).

**Table 2:** Engagement results (N=220).

			Total		StressLess (n=92)		Breeze (n=66)		TragicKingdom (n=62)		Chi-square (*df*)			*F* test (*df*)			*P* value	
**Operating system, n (%)**												12.9 (4)			—^a^			.002
	Android	54 (24.5)		18 (19.6)		27 (40.9)		9 (14.5)										
	iOS	160 (72.7)		71 (77.2)		39 (59.1)		50 (80.6)										
	Unknown	6 (2.7)		3 (3.3)		0 (0)		3 (4.8)										
**Chatbot sex, n (%)**												0.3 (4)			—			.86
	Female	100 (45.5)		43 (46.7)		30 (45.5)		27 (43.5)										
	Male	112 (50.9)		44 (47.8)		36 (54.5)		32 (51.6)										
	Not set	8 (3.6)		5 (5.4)		0 (0)		3 (4.8)										
**Milestone, n (%)**																		
	0: choose coach	212 (96.4)		87 (94.6)		66 (100)		59 (95.2)		3.6 (2)			—			.17		
	1: administrative and consent phase	163 (74.1)		66 (71.7)		48 (72.7)		49 (79)		1.1 (2)			—			.57		
	2: nickname	142 (64.5)		57 (62)		41 (62.1)		44 (71)		1.6 (2)			—			.46		
	3: ongoing dialogue	134 (60.9)		52 (56.5)		39 (59.1)		43 (69.4)		2.7 (2)			—			.26		
	4: prepare for SPBT^b^	128 (58.2)		48 (52.2)		37 (56.1)		43 (69.4)		4.7 (2)			—			.10		
	5: end SPBT	21 (9.5)		7 (7.6)		8 (12.1)		6 (9.7)		0.9 (2)			—			.64		
	6: complete intervention	17 (7.7)		5 (5.4)		7 (10.6)		5 (8.1)		1.5 (2)			—			.48		
	7: survey completion	13 (5.9)		4 (4.3)		4 (6.1)		5 (8.1)		0.9 (2)			—			.63		
**Breakdown of milestone 1** **:** **administrative and consent phase, n (%)**																		
	Screening	182 (82.7)		76 (82.6)		55 (83.3)		51 (82.3)		0.03 (2)			—			.99		
	Agreement to terms and conditions	176 (80)		72 (78.3)		54 (81.8)		50 (80.6)		0.3 (2)			—			.85		
	Microphone access	163 (74.1)		66 (71.7)		48 (72.7)		49 (79)		1.1 (2)			—			.57		
Conversational turns (if milestone 0=yes), mean (SD; range)			48.3 (22.64; 9-85)		45.5 (21.35; 10-77)		46.3 (23.4; 12-77)		54.7 (22.7; 9-85)		—			3.33 (2, 209)			.04	
**Time elapsed since download (min), mean (SD)**																		
	Milestone 1: administrative and consent phase	0.6 (0.56)		0.6 (0.54)		0.7 (0.57)		0.5 (0.57)		—			0.977 (2, 151)			.37		
	Milestone 2: nickname	1.4 (0.91)		1.3 (0.99)		1.2 (0.78)		1.6 (0.89)		—			1.993 (2, 128)			.14		
	Milestone 3: ongoing dialogue	2.4 (13.8)		2.4 (1.49)		2.6 (1.55)		2.2 (1.05)		—			0.940 (2, 117)			.39		
	Milestone 4: prepare for SPBT	4.1 (5.81)		3.4 (2.13)		6.5 (10.12)		2.7 (1.45)		—			4.683 (2, 115)			.01		
	Milestone 5: end SPBT	7.4 (4.15)		8.6 (4.11)		8.0 (4.24)		4.0 (3.29)		—			1.344 (2, 12)			.30		
	Milestone 6: complete intervention	8.6 (4.37)		9.8 (4.53)		8.9 (4.57)		5.3 (4.09)		—			0.676 (2, 9)			.53		

^a^Not applicable.

^b^SPBT: slow-paced breathing training.

In total, 50.9% (112/220) of all participants chose the male version of the chatbot, with no significant differences between the app versions (Breeze: 36/66, 55%; TragicKingdom: 32/62, 52%; StressLess 44/92, 48%; *ꭓ*^2^_2, 212_=0.3, *P*=.86). A total of 3.6% (8/220) of all participants did not choose a character, indicating that they did not start the conversation with the chatbot, either.

**Figure 9:**
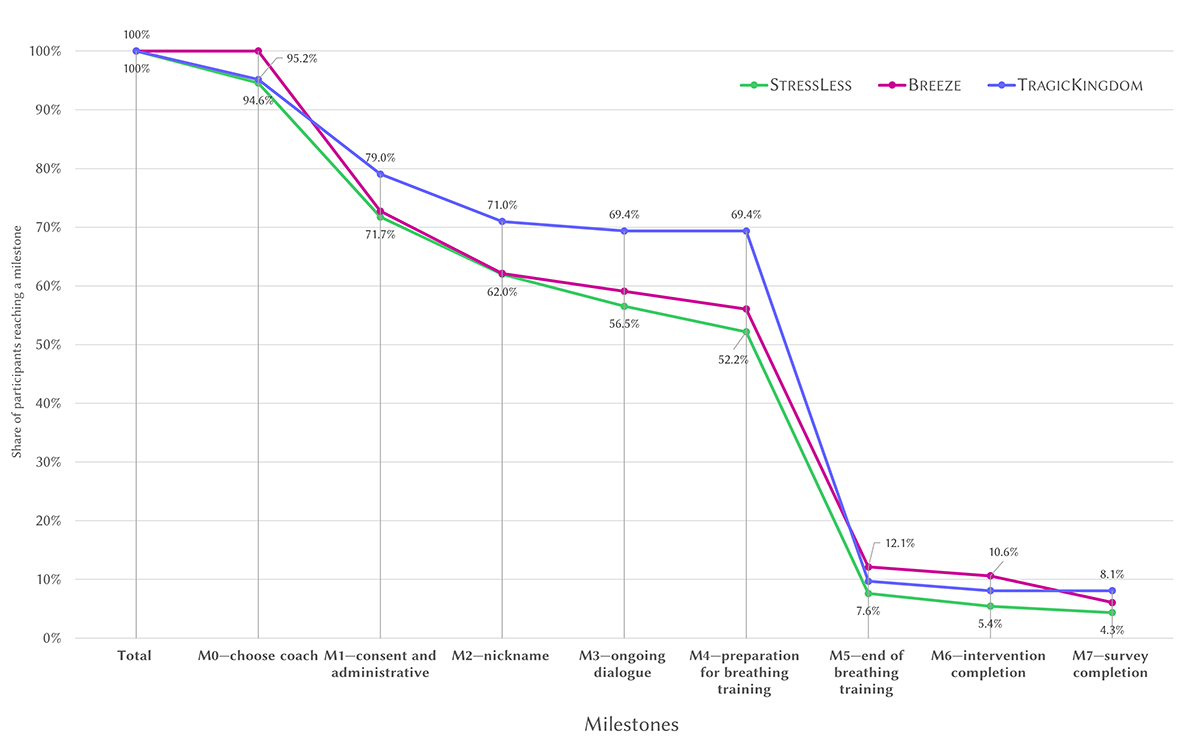
Share of participants per milestone and condition.

Figure 9 further illustrates the number of users who completed the predefined milestones. Across all interventions, 96.4% (212/220) of the participants successfully chose a coach at the beginning of the study (milestone 0), with no significant differences between groups. Progressively fewer participants completed each subsequent milestone: 74.1% (163/220) completed the administrative and consent phase (milestone 1), 64.5% (142/220) created a nickname (milestone 2), and 60.9% (134/220) engaged in ongoing dialogue (milestone 3), with none of these showing statistically significant differences between interventions. In addition, 58.2% (128/220) of the participants prepared for the breathing training (milestone 4), with a marginally significant difference across interventions (*P*=.10). The highest number of dropouts (83.5% on average) then occurred between the milestones preparation for breathing training (milestone 4) and end of breathing training (milestone 5). Only 9.5% (21/220) completed the breathing training (milestone 5). However, differences in dropouts between these milestones across conditions were not significant (StressLess: 41/48, 85%; Breeze: 29/37, 78%; TragicKingdom: 37/43, 86%; *χ*^2^_2_=1.0, *P*=.60).

Of the 220 users who downloaded the app after (or while) watching their respective Twitch streams, 17 (7.7%) completed the intervention (milestone 6). Breeze had the highest intervention completion rate, with 11% (7/66) completing the intervention, followed by TragicKingdom (5/62, 8%) and StressLess (5/92, 5%), with no significant differences between conditions. Similarly, 5.9% (13/220) completed the postsurvey at the end of the intervention (milestone 7).

The number of conversational turns (for those who selected a coach [milestone 0] and started interacting with the chatbot) ranged from 9 to 85, with an average of 48.3 (SD 22.64) exchanged messages across all conditions, with significant differences across the interventions (*F*_2,209_=3.330; *P*=.04). Participants in the TragicKingdom condition had the highest average number of conversational turns (mean 54.7, SD 22.7), followed by Breeze (mean 46.3, SD 23.4) and StressLess (mean 45.5, SD 21.35).

Regarding the total time elapsed in minutes from the start of the intervention per milestone, there only was a significant difference across interventions for milestone 4. . For example, the mean time to complete the administrative and consent phase (milestone 1) ranged from 0.5 to 0.7 minutes across conditions. The mean time spent in the ongoing dialogue (milestone 3) ranged from approximately 2.0 to 2.4 minutes across conditions, with no significant differences. The preparation for the slow-paced breathing training (milestone 4) showed a significantly longer average time in Breeze (mean 6.5, SD 10.2 minutes) compared to the other conditions (*F*_2,118_=4.683; *P=*.01). Finally, participants spent an average of 7.4 to 8.9 minutes completing the entire intervention (milestone 6), without significant differences between groups (*F*_2,9_=0.676; *P*=.53).

#### Breathing Training

Table 3 summarizes participants’ engagement with the breathing training across the 3 interventions.

**Table 3:** Descriptive results of the engagement with the breathing training.

		Total	StressLess	Breeze	TragicKingdom
**Milestone 4: preparation for breathing training**					
	Total completes, n	128	48	37	43
	Personalized breathing training, n/N (%)	86/128 (67.2)	31/48 (64.6)	27/37 (73)	28/43 (65.1)
**Milestone 5: end of breathing training**					
	Total completes, n (%)	21	7	8	6
	Personalized breathing training, n/N (%)	13/21 (61.9)	5/7 (71.4)	5/8 (62)	3/6 (50)
	Interaction duration (min), mean (SD)	3.9 (2.61)	3.8 (2.67)	3.9 (3.15)	3.8 (1.95)
	Adjusted interaction duration (min)^a^, mean (SD)	3.2 (1.65)	3.1 (1.71)	2.9 (1.75)	3.6 (1.72)
	Distance of breathing training (miles), mean (SD)	2204 (1640)	1775 (1479)	2358 (1791)	2471 (1912)
Mood change, mean (SD; range)		0.4 (1.39; –2 to 4)	1.2 (1.60; 0 to 4)	−0.3 (1.28; –2 to 1)	0.6 (0.89; 0 to 2)

^a^The time spent on the breathing training included the time spent on the tutorial at the beginning of the training. The training lasted a maximum of 5 minutes. Therefore, larger numbers were adjusted to 5 minutes. However, the other values were not adjusted to the time spent on the tutorial.

In total, 128 participants completed the dialogue in preparation for the breathing training (milestone 4). A total of 67.2% (86/128) of the participants opted to personalize their breathing training, with the highest percentage observed in the Breeze group (27/37, 73%) and the lowest observed in TragicKingdom (28/43, 65%). However, only 21 participants completed the breathing training (milestone 5; n=7, 33% in StressLess; n=8, 38% in Breeze; n=6, 29% in TragicKingdom), of whom 13 (62%) opted to personalize the breathing training.

The average adjusted interaction time across participants who completed the training amounted to 3.2 (SD 1.65) minutes (mean* *interaction duration 3.9, SD 2.61 min), with Breeze showing the highest adjusted mean time at 3.6 (SD 1.72) minutes and StressLess showing the lowest at 3.1 (SD 1.71) minutes.

In addition, the distance covered during the breathing training averaged 2204 (SD 1640) miles across all participants, with TragicKingdom having the highest mean distance (2471, SD 1912 miles), followed by Breeze (2358, SD 1791) and StressLess (1775, SD 1479 miles). Moreover, 71% (5/7) of the users who conducted the breathing training chose the personalized option.

In terms of emotional mood changes after the training, participants experienced a small average positive change (mean 0.4, SD 1.39), with StressLess showing the highest average change (mean 1.2, SD 1.60).

#### Survey Overview

The descriptive statistics of the survey participants are shown in Table 4. Of the 13 participants who completed the survey, 8 (62%) were female, and on average, they were aged 26.15 (SD 11.97) years. Furthermore, 54% (7/13) of the participants were from Switzerland, 31% (4/13) were from Germany, and 15% (2/13) were from Austria. The subjectively rated general health status was high (mean 4.23, SD 0.58). As the sample size of the survey was considerably small (StressLess: 4/13, 31%; Breeze: 4/13, 31%; TragicKingdom: 5/13, 38%), a differentiated analysis in terms of each intervention was not conducted.

**Table 4:** Descriptive statistics of the participants who completed the survey (N=13).

				Values
**Responses per condition, n (%)**				
	StressLess		4 (31)	
	Breeze		4 (31)	
	TragicKingdom		5 (38)	
**Sociodemographics**				
	Age (y), mean (SD)		26.15 (11.97)	
	Sex (female), n (%)		8 (62)	
	**Country, n (%)**			
		Switzerland	7 (54)	
		Germany	4 (31)	
		Austria	2 (15)	
General health status: self-assessed (score of 1-5), mean (SD)				4.23 (0.58)
Participation in lottery (yes), n (%)				6 (46)

#### SES of Survey Participants

The survey questions were designed to assess the SES of participants who completed the intervention. Table 5 provides an overview of the SES classifications and the distribution of SES scores per condition and for the total sample. Detailed calculations are provided in Multimedia Appendix 1 (section E1).

**Table 5:** Socioeconomic status (SES) scores per condition and for the total sample (N=13).

	Total, mean (SD)	StressLess (n=4), mean (SD)	Breeze (n=4), mean (SD)	TragicKingdom (n=5), mean (SD)
Educational level (range 1-7)	4.86 (1.28)	5.08 (1.38)	5.60 (1.69)	4.10 (0.64)
Occupation (range 1-7)	4.49 (1.11)	5.05 (1.30)	4.25 (0.30)	4.24 (1.50)
Income (range 1-7)	5.08 (1.72)	5.50 (1.47)	6.13 (1.03)	3.90 (2.04)
SES score (range 3-21)	14.43 (2.92)	15.63 (3.68)	15.98 (2.48)	12.24 (1.74)

The average SES score across all participants was 14.43 (SD 2.92), with scores ranging from 9.6 to 21.

None of the participants could be classified as being of a low SES (SES score range 3.2-8.7 [107]); 77% (10/13) of the participants were classified as being of a middle SES (SES score range 8.8-16.9), whereas 23% (3/13) were in the high-SES category (SES score range 17-21). Among the interventions, TragicKingdom had the lowest mean SES score (mean 12.24, SD 1.74), followed by StressLess (mean 15.63, SD 3.63) and Breeze (mean 15.98, SD 2.48).

#### Technology Acceptance

Participants’ technology acceptance perceptions regarding the DHI versions are shown in Table 6. All mean scores exceeded the neutral point of 2.5, indicating generally positive feedback. Specifically, participants found the app versions easy to use (mean 4.62, SD 0.49) and enjoyable (mean 4.15, SD 0.53). However, the intention to use the app was slightly lower (mean 3.62, SD 1.00). Detailed qualitative feedback, both positive and negative, can be found in Multimedia Appendix 1 (section E2).

**Table 6:** Technology acceptance measures per condition and for the total sample (N=13).

	Total, mean (SD)	StressLess (n=4), mean (SD)	Breeze (n=4), mean (SD)	TragicKingdom (n=5), mean (SD)
Ease of use (score of 1-5)	4.62 (0.49)	4.50 (0.58)	4.75 (0.50)	4.60 (0.55)
Enjoyment (score of 1-5)	4.15 (0.53)	4.25 (0.50)	4.00 (0.82)	4.20 (0.45)
Use intention (score of 1-5)	3.62 (1.00)	3.75 (0.96)	3.50 (1.29)	3.60 (1.14)

## Discussion

### Principal Findings

Overall, the results presented in this paper show that using Twitch for digital health recruitment is highly promising, moving far beyond some previously published reach ratios for DHIs using traditional social media websites (eg, 0.02% [108] and 0.1% [109]). However, balanced against this potential are occasionally low reach ratios by certain streamers (eg, 2/3526, 0.1%; stream 4-3 of streamer ID 4), which, by comparison, are no better than direct email campaigns to known contacts (eg, 2.5% using email with health insurance customers [110]), which are far simpler and cheaper to use. Therefore, the results presented in this paper indicate a mixed picture—while influencer-based recruitment strategies on Twitch can reach large audiences and may be a highly effective digital health recruitment tool, simply adopting an influencer strategy is not enough to guarantee success.

However, an important caveat in interpreting these findings were varying levels of engagement with the actual intervention content following app download. While initially high levels of engagement were found (ie, downloading the app and beginning the interaction with the chatbot), a substantial drop-off was evidenced, particularly during the gamified breathing training, where only 7.7% (17/220) of the participants completed the training in full. Adherence is a known challenge in digital health [111], and our findings show that, despite the potential of streaming platforms such as Twitch to recruit participants, it does not provide a panacea to resolve the well-documented dropout rates established across digital health domains [112]. While incentives—such as giveaways or monetary compensation—have been shown to increase adherence in digital health studies [95], their scalability and ethical applicability remain limited in public health domains, particularly in the context of prevention, in which no established reimbursement structures exist. For instance, Germany’s digital health application framework only covers therapeutic interventions for individuals with a formal diagnosis [113-115], underscoring the absence of a structured market for preventive DHIs. Nonetheless, incentives embedded within influencer-driven formats could represent a promising direction for future research.

Reasoning as to why these dropouts occurred, we offer several explanations. First, our rationale for using the Twitch platform was the likely presence of 2 risk factors among its audience, namely, habitual video game [116] and social media use [117]. However, the apps presented in our study may have been less appealing than the professionally developed video games or social media activities present on the platform, subsequently leading participants to return to Twitch rather than complete the breathing exercise in full. Second, relatedly, there is a strong tendency toward immediate gratification when using digital media [118], particularly among the younger demographics [119]. Thus, while the health benefits of the apps were presented by the streamers, these benefits may have been too intangible, contributing to dropouts. Third, the timing of DHIs is key [120]. However, participants were using Twitch, an entertainment platform used for recreation and relaxation purposes [121]. Thus, while participants may have indeed seen the value in the stress-based biofeedback game (leading to their download), they might not have been acutely stressed in the first place and, thus, had no reason to complete the breathing exercise at that time. We cannot rule out the influence of technical issues during the breathing training as no crash diagnostics or in-app analytics were available (which should be included in future studies) to distinguish disengagement from technical dropouts.

As a final point, while the recruitment strategy effectively reached a large audience (N=220) with an overall average reach ratio of 16.2%, we were unable to confirm the hypothesis that Twitch could engage low-SES individuals within their comfort zones due to a lack of survey responses. In contrast to other social media platforms, Twitch does not allow for the capturing of detailed audience metrics, which is perhaps why most extant research has focused on influencer-level analyses [122-124] rather than on the audience. In any case, because of this, we cannot assess the extent to which vulnerable individuals were reached despite our successes in overall recruitment. In hindsight, and for future research, we recommend integrating key SES-related screening questions early in the intervention flow (eg, during onboarding) or, alternatively, implementing a separate pre- or postintervention survey dedicated to SES assessment. Such an approach would allow for more robust verification of whether the intended vulnerable populations are being reached while minimizing disruption to the intervention experience itself.

### Factors Governing the Effectiveness of Influencer Recruitment

On the basis of the findings presented in this paper, alongside those presented in extant research, we propose 3 factors governing the effectiveness of adopting an influencer-based strategy using streamers on Twitch for recruitment and health promotion purposes.

First, influencers vary greatly in terms of their communicative, interpersonal style [125], and this individual-level heterogeneity can lead to both differences in the ability to promote health content effectively and likely differences in the makeup of the audience. While we were unable to capture the audience background in this domain (due to constraints of Twitch and low survey response rates; also refer to the Limitations section), communication style has been found to be an important determinant in the recruitment for novel digital health tools [126], and matching health promotion content with the expectations of vulnerable individuals has a long history in health care research [127]. Indeed, in nonhealth domains, the communicative style of influencers has been shown to affect both advertising relevance and audience expectations [128]. Thus, we reason that carefully selecting influencers whose public personas and communication styles align well with the intervention’s goals and the values of the targeted social milieu is likely to lead to higher effectiveness. In addition, upskilling, educating, or “influencing influencers,” as described in the study by Motta et al [34], may further improve their ability to convey health-related content in a way that is both authentic and motivating.

Second, the effectiveness of Twitch influencers was found to be more pronounced among smaller-scale streamers or microinfluencers. These influencers, who typically have fewer followers, appear to foster a more authentic connection via the greater time they can afford in personalized interactions with followers [106]. Indeed, greater interactivity is a common factor in the success of various novel technological platforms deployed in digital health [129], such as self-service technologies [130] or chatbots [131], and a strength of social media–based marketing more generally [132]. Thus, we reason that this rationale similarly extends to Twitch or influencer marketing, where greater attention spent by an influencer on each participant facilitated “parasocial relationship” (ie, an imagined intimate relationship with the streamer) building, a phenomenon documented on Twitch previously [121]. Accordingly, future interventions could explore the integration of real-time interaction between influencers and participants (eg, live questions and answers or feedback loops during the intervention) to further strengthen social presence and motivation.

Third, the streamers mostly streamed content in the categories *games* and *IRL* and for varying durations. Therefore, it is reasonable to assume that both the category selected and the duration for which the gamified breathing exercise was presented influenced downloads. Indeed, data gathered showed that longer streams were actually counterproductive in generating downloads, likely due to the “try before you buy” phenomenon on Twitch (ie, users had vicariously played the entire app content and, thus, had no further need to download the app) [121]. Similarly, we reason that the choice of categories (eg, *game*) or specific subcategories (eg, comparing Minecraft to Grand Theft Auto) that differ in terms of congruency to a relaxing breathing exercise may have influenced the downloads.

### Limitations

This study featured real streamers promoting real health apps, capturing highly relevant behavioral data (ie, app downloads and app engagement) and, thus, possesses a high degree of external validity for digital health recruitment. That being said, there are also a number of limitations in study design and setup that should be acknowledged when interpreting the results.

First, as noted, due to limited survey responses after the intervention, we were unable to assess the audience of those downloading the app and, thus, could not assess demographics or the vulnerability status of the users, a core aim of this research project. Moreover, while the DHI designs were tailored to align with the value orientations of the SINUS Institute social milieux, they were not directly cocreated with the influencers’ audiences or informed by specific audience needs assessments. However, this choice reflects our study’s focus on testing theoretical reach and engagement mechanisms in a controlled field setting using 2 previously evaluated versions (StressLess and Breeze [64,67,133]) and 1 newly developed, dedicated version (TragicKingdom) designed with the Twitch platform’s audience profile in mind (also refer to the Future Research section).

Second, due to the inherent nature of live streaming, health messages were not delivered in a fully standardized manner by streamers, which likely introduced additional variability in download frequencies. While we compensated this by providing standardized scripts and content to streamers, streamers were also encouraged to retain their personal communicative style so that we could organically assess the potential of streamers to promote health-related content effectively. In addition, while we used 2 streamers per genre to balance out strong individual effects unique to a single streamer, it is possible that the audiences varied between each of these streamers in terms of their health vulnerability [124], in turn influencing downloads. Together, these issues critically highlight the inherent challenges of aligning digital channels and DHI designs with the value orientations of specific social milieux and the need to consider distinct platforms for distinct milieux (also refer to the Future Research section).

Third, the 3 experimental conditions varied several elements simultaneously (eg, related to the chatbot, video style, and influencer) between conditions. Although this was necessary to match DHI design with the social milieux established by the SINUS Institute [43], this research design meant that it was not possible to fully disentangle the relative effects of each experimental condition individually. Relatedly, our design of the “sensation-oriented” TragicKingdom version raises important considerations about what constitutes a DHI in the first place. While this version did not explicitly frame the activity as health related, it was designed to promote health-relevant behavior through gamified engagement with stress-reducing breathing exercises. While this broadly aligns with emerging research on so-called stealth health interventions [38,39], which emphasizes the potential of implicit, play-based strategies, future research should further explore the effectiveness and acceptance of such approaches in comparison to more explicitly health-framed interventions.

Finally, while the interventions were designed as low burden, intuitive, and accessible with simplified language and scripted chatbot dialogues tailored for laypersons, we acknowledge that digital health literacy remains a key factor influencing access, understanding, and sustained engagement, particularly among socioeconomically vulnerable groups. Given the well-documented disparities in digital health literacy [27], future research might explore combining milieu-specific DHI tailoring and influencer-based outreach with explicit DHI support measures such as digital onboarding or user education strategies to further reduce barriers and ensure equitable uptake.

### Future Research

Understanding the recruitment for DHIs is an emerging research area that has faced increasing scrutiny in recent years. Nevertheless, the topic remains relatively immature when compared to the wealth of data on DHIs and treatment outcomes. Therefore, we offer several research directions that should prove useful in expanding this domain.

First, investigating congruency between the promoted app or intervention and the personality of the influencer would likely prove fruitful. Research has shown that congruency between social media advertisement content and audience values or demographics has a strong influence in their effectiveness as a recruitment tool [134-136]. Thus, understanding how certain streamers may more effectively promote health-related content among certain audiences (particularly those exhibiting higher vulnerability) would enable more effective targeting. In this vein, future research could build on our findings by incorporating further formative research, audience profiling, and participatory design methods to ensure that the interventions even more closely reflect the lived experiences, preferences, and perceived needs of the target groups. In a similar vein, qualitative postintervention assessments or follow-up interviews could provide valuable insights into user experiences and engagement trajectories, especially to better understand dropout reasons.

Second, a comparison of different social media platforms for recruiting vulnerable participants would be highly welcome. While reviews are available on recruitment and health promotion efforts across various social media platforms [137], these are hindered by being made up of papers that vary widely in scope (eg, health domain, audiences, funding, duration, and other factors). Therefore, it would be useful to have a single study that controls for these various factors and uses multiple social media platforms simultaneously to recruit participants. This would enable a relative ranking of the strengths or weaknesses of such platforms among different audiences.

Third, the use of virtual influencers (ie, artificially created characters combining the use of computer-generated images and artificial intelligence [138]) for health promotion purposes would be an interesting new research direction. Virtual influencers have found success in commercial settings for advertising products on social media [139] and were even experimentally used by the World Health Organization during the COVID-19 pandemic [140]. Therefore, we reason that further exploration of their capabilities for health promotion among vulnerable groups may open up a new method of recruitment. This would be particularly interesting considering the rise of increasingly powerful large language models that, once integrated with virtual influencers, will inevitably improve their realism, authenticity, and ability to promote health content [141].

Finally, to the authors’ knowledge, there is not yet a large-scale study that integrates social media recruitment, intervention adherence, and longitudinal health outcomes. In the context of this study, this could have been achieved by extending the study design and capturing relevant biomarkers gathered during the biofeedback breathing training. Doing so would greatly improve our understanding of how social media can be leveraged to actually improve health outcomes over the long term and would be particularly impactful if targeted toward vulnerable individuals.

### Conclusions

In this study, we successfully demonstrated the potential of Twitch for digital health recruitment, with high reach ratios that, at times, vastly outperformed those of other digital health recruitment tools. Nonetheless, we were unable to verify the assumption that Twitch could target and engage vulnerable individuals within their comfort zones due to a high number of dropouts. Moving forward, we encourage future research to gather both more audience-level and more streamer-level data so that innovative recruitment strategies can be developed to meet vulnerable individuals within their comfort zones. Doing so will ensure the equitable rollout of DHIs across the population; this study takes an important first step toward this goal.

## Appendix

Multimedia Appendix 1 Supplementary material.

## Abbreviations

DHI: digital health intervention
NCD: noncommunicable disease
RQ: research question
SES: socioeconomic status

## Data Availability

The datasets generated or analyzed during this study are available from the corresponding author on reasonable request.
