# Supplementary material for: Leveraging Influencers to Reach and Engage Vulnerable Individuals With a Digital Health Intervention: Quasi-Experimental Field Study
Source: J Med Internet Res. 2025 Sep 5;27:e67174. doi: 10.2196/67174 (PMC12449675; doi:10.2196/67174)
Supplement: Multimedia Appendix 1 [file jmir_v27i1e67174_app1.pdf]

Leveraging Influencers to Reach and Engage Vulnerable Individuals With a Digital Health Intervention: Quasi-Experimental Field Study

Multimedia Appendix

This is the Multimedia Appendix to the manuscript "Leveraging Influencers to Reach and Engage Vulnerable Individuals With a Digital Health Intervention: Quasi-Experimental Field Study" published in the J Med Internet Res by Nißen et al. in 2025

For full copyright and citation information see http://dx.doi.org/10.2196/67174

Table of Contents

Appendix A DEVELOP the interventions ... 3
A1 Conversational scripts of the interventions ... 3
A2 Detailed information on milestones ... 8
A3 Screenshots of the design features in the interventions ... 10
A3.1 Selection conversational agent (Alexander vs. Alexandra) ... 10
A3.2 Welcome screen ... 11
A3.3 Admin and consent dialogue ... 11
A3.4 Exemplary chatbot interactions ... 12
A3.5 Additional features (exemplary for STRESSLESS) ... 14
A3.6 Breathing exercise - introductory slides (exemplary for BREEZE) ... 15
Appendix B RECRUIT influencers ... 16
B1 Material for influencers ... 16
B1.1 Leading principles and information for the streamers ... 16
B1.2 Scripts for the streamers ... 17
B1.3 Banner with QR-Code ... 20
B1.4 Promotional videos ... 21
Appendix C REACH participants ... 22
C1 Impressions of the advertisement streams ... 22
C2 Screenshot of Exemplary Twitchtracker Archival Data ... 26
C3 Notes on influencer strategy ... 27
Appendix D ENGAGE participants ... 28
D1 Terms and conditions ... 28
D2 Measurements ... 30
D2.1 Chatbot-based: mood state questionnaire ... 30
D2.2 Survey-based: sex, age, country of residence, general health status ... 30
D2.3 Survey-based: socioeconomic status questions (education, occupation, income) ... 30
D2.4 Survey-based: user experience ... 32
D2.5 Survey-based: qualitative feedback ... 32
Appendix E EVALUATE results ... 33
E1 Notes on socioeconomic status (SES) index calculation ... 33
E2 Qualitative feedback from participants ... 36
References ... 37

Appendix A DEVELOP the interventions

A1 Conversational scripts of the interventions

Appendix A - Conversational Scripts

StressLess		
**Phrase**	**DE**	**EN**
**Start &<br>User agreement**	Bevor wir loslegen ist es wichtig, dass du die folgenden Kriterien erfüllst:<br>1. Ich spreche Deutsch.<br>2. Ich bin volljährig.<br>3. Ich leide NICHT an Herz-Kreislauf-, Atemwegs- oder psychischen Erkrankungen.	Before we start, it is important that you fulfill the following criteria:<br>1. I speak English.<br>2. I am an adult.<br>3. I do not have any cardiovascular, respiratory, or psychological illnesses.
	☑ Ich erfülle alle Kriterien: yes<br>✖ Ich erfülle nicht alle Kriterien: no	☑ I meet all criteria: yes<br>✖ I do not meet all criteria: no
	DE	Please let me know if you agree to the terms of use of the app.
	Nutzungsbedingungen	*User terms and conditions*
	☑ Ich stimme zu<br>✖ Ich stimme nicht zu	☑ I agree<br>✖ I do not agree
Condition "no"	Bist du dir sicher, dass du den Nutzungsbedingungen nicht zustimmen möchtest?	Are you sure that you do not want to consent to the user terms and conditions?
	Ja, ich bin mir sicher (beenden)<br>Nein, gib mir nochmals eine Chance (bleiben)	Yes, I am sure (quit)<br>No, give me one more chance (stay)
	Noch eine letzte Bitte. Damit die App funktioniert ist der Zugriff auf das Mikrofon deines Smartphones notwendig. Kannst du dies bitte kurz erlauben?	One final favor to ask: For this app to function properly, it needs permission to access your smartphone's microphone. Would you mind granting access to the microphone, if you haven't already done so?
	☑ ja klar: yes<br>✖ Nein, dann muss ich leider passen: no	☑ Yes, sure!: yes<br>✖ No, unfortunately I have to pass: no
Condition "no"	Ups, hast du aus Versehen oder mit Absicht den Zugriff zum Mikrofon abgelehnt?	Oops, did you accidentially or intentionally disable your microphone access?
	Ups, das war ein Versehen, kann ich nochmal?:yes<br>Ich habe mir es anders überlegt und nehme doch nicht teil.:no	Oops, that was a mistake, can I try again?:yes<br>I have changed my mind and will not participate after all.:no
Screenout<br>Condition "no"	Leider erfüllst du die Kriterien zur Teilnahme an der Studie nicht. Du kannst die App jetzt löschen.	Unfortunately, you do not meet the criteria for participating in the study. You can now delete the app.
	Besten Dank für deine Rückmeldung.<br>Du kannst die App jetzt löschen.	Thank you for the feedback. You can delete the app now.
**Introduction**	Danke dir und herzlich willkommen.	Thank you and welcome!
	-	-
	Ich bin $coachName. Ich bin ein digitaler Experte für Stress-Situationen.	I am $coachName. I am an digital expert for stress situations.
	-	-
	-	-
	-	-
	-	-
	-	-
	-	-

Breeze		
**Phrase**	**DE**	**EN**
**Start &<br>User agreement**	Bevor wir loslegen ist es wichtig, dass du die folgenden Kriterien erfüllst:<br>1. Ich spreche Deutsch.<br>2. Ich bin volljährig.<br>3. Ich leide NICHT an Herz-Kreislauf-, Atemwegs- oder psychischen Erkrankungen.	Before we start, it is important that you fulfill the following criteria:<br>1. I speak English.<br>2. I am an adult.<br>3. I do not have any cardiovascular, respiratory, or psychological illnesses.
	☑ Ich erfülle alle Kriterien: yes<br>✖ Ich erfülle nicht alle Kriterien: no	☑ I meet all criteria: yes<br>✖ I do not meet all criteria: no
	Sage mir noch, ob du mit der Nutzung dieser App einverstanden bist.	Please let me know if you agree to the terms of use of the app.
	Nutzungsbedingungen	*User terms and conditions*
	☑ Ich stimme zu: yes<br>✖ Ich stimme nicht zu: no	☑ I agree<br>✖ I do not agree
Condition "no"	Bist du dir sicher, dass du den Nutzungsbedingungen nicht zustimmen möchtest?	Are you sure that you do not want to consent to the user terms and conditions?
	Ja, ich bin mir sicher (beenden)<br>Nein, gib mir nochmals eine Chance (bleiben)	Yes, I am sure (quit)<br>No, give me one more chance (stay)
	Noch eine letzte Bitte. Damit die App funktioniert ist der Zugriff auf das Mikrofon deines Smartphones notwendig. Kannst du dies bitte kurz erlauben?	One final favor to ask: For this app to function properly, it needs permission to access your smartphone's microphone. Would you mind granting access to the microphone, if you haven't already done so?
	☑ ja klar<br>✖ Nein, dann muss ich leider passen	☑ Yes, sure!<br>✖ No, unfortunately I cannot do it
Condition "no"	Ups, hast du aus Versehen oder mit Absicht den Zugriff zum Mikrofon abgelehnt?	Oops, did you accidentially or intentionally disable your microphone access?
	Ups, das war ein Versehen, kann ich nochmal?:yes<br>Ich habe mir es anders überlegt und nehme doch nicht teil.:no	Oops, that was a mistake, can I try again?:yes<br>I have changed my mind and will not participate after all.:no
Screenout	Leider erfüllst du die Kriterien zur Teilnahme an der Studie nicht. Du kannst die App jetzt löschen.	Unfortunately, you do not meet the criteria for participating in the study. You can now delete the app.
	Besten Dank für deine Rückmeldung.<br>Du kannst die App jetzt löschen.	Thank you for the feedback. You can delete the app now.
**Introduction**	Hi, wie geht es dir?	Hi, how are you?
	Hi, alles bestens 😀<br>Hey, heute geht es mir nicht besonders gut<br>Hallo 👋	Hi, everything fine 😀<br>Hey, today I am not doing so good.<br>Hello 👋
	Ich bin $coachName, dein digitaler Coach für Stresssituationen. Gut für zwischendurch. 💪	I am $coachName, your digital coach for stressful situations. Good for moments in between. 💪
	-	-
	Es freut mich dich kennenzulernen. 😊<br>Toll, so jemanden wollte ich schon immer. 😄	I am happy to get to know you. 😊<br>Great, I have always wanted someone like this. 😄
	-	-
	-	-
	-	-
	-	-

Tragic Kingdom		
**Phrase**	**DE**	**EN**
**Start &<br>User agreement**	Bevor wir loslegen ist es wichtig, dass du die folgenden Kriterien erfüllst:<br>1. Ich spreche Deutsch.<br>2. Ich bin volljährig.<br>3. Ich leide NICHT an Herz-Kreislauf-, Atemwegs- oder psychischen Erkrankungen.	Before we start, it is important that you fulfill the following criteria:<br>1. I speak English.<br>2. I am an adult.<br>3. I do not have any cardiovascular, respiratory, or psychological illnesses.
	☑ Ich erfülle alle Kriterien: yes<br>✖ Ich erfülle nicht alle Kriterien: no	☑ I meet all criteria: yes<br>✖ I do not meet all criteria: no
	Sage mir noch, ob du mit der Nutzung dieser App einverstanden bist.	Please let me know if you agree to the terms of use of the app.
	Nutzungsbedingungen	*User terms and conditions*
	☑ Ich stimme zu<br>✖ Ich stimme nicht zu	☑ I agree<br>✖ I do not agree
Condition "no"	Bist du dir sicher, dass du den Nutzungsbedingungen nicht zustimmen möchtest?	Are you sure that you do not want to consent to the user terms and conditions?
	Ja, ich bin mir sicher (beenden)<br>Nein, gib mir nochmals eine Chance (bleiben)	Yes, I am sure (quit)<br>No, give me one more chance (stay)
	Noch eine letzte Bitte. Damit die App funktioniert ist der Zugriff auf das Mikrofon deines Smartphones notwendig. Kannst du dies bitte kurz erlauben?	One final favor to ask: For this app to function properly, it needs permission to access your smartphone's microphone. Would you mind granting access to the microphone, if you haven't already done so?
	☑ ja klar: yes<br>✖ Nein, dann muss ich leider passen: no	☑ Yes, sure!: yes<br>✖ No, unfortunately I have to pass: no
Condition "no"	Ups, hast du aus Versehen oder mit Absicht den Zugriff zum Mikrofon abgelehnt?	Oops, did you accidentially or intentionally disable your microphone access?
	Ups, das war ein Versehen, kann ich nochmal?:yes<br>Ich habe mir es anders überlegt und nehme doch nicht teil.:no	Oops, that was a mistake, can I try again?:yes<br>I have changed my mind and will not participate after all.:no
Screenout<br>Condition "no"	Leider erfüllst du die Kriterien zur Teilnahme an der Studie nicht. Du kannst die App jetzt löschen.	Unfortunately, you do not meet the criteria for participating in the study. You can now delete the app.
	Besten Dank für deine Rückmeldung.<br>Du kannst die App jetzt löschen.	Thank you for the feedback. You can delete the app now.
**Introduction and Storytelling**	-	-
	-	-
	**"$coachname = Alexander" -** Willkommen, ich bin $coachName und hersche alleine über Dooma seitdem meine liebste Frau und Königin Alexandra kaltblütig ermordet wurde.<br><br>**If $coachname = Alexandra:** Willkommen, ich bin $coachName und hersche alleine über Dooma seitdem mein liebster Mann und König Alexander kaltblütig ermordet wurde.<br>---	"$coachname = Alexander Welcome, I am $coachName, reigning alone over the Dooma kingdom ever since the heart-wrenching day my beloved wife and Queen Alexandra was tragically taken from this world, her life snuffed out in a brutal act of murder.<br><br>"$coachname = Alexandra" - Welcome, I am $coachName, reigning alone over the Dooma kingdom ever since the heart-wrenching day my beloved husband and King Alexander was tragically taken from this world, his life snuffed out in a brutal act of murder.
	Ich habe von deinen Skills gehört und möchte dir ein Angebot machen.	I have heard of your skills, and I would like to make you an offer.
	Um was geht es?	What is it?
**Storytelling**	Ich benötige dich für einen enorm wichtigen Einsatz. ⚔ 🛡<br>---<br>Es ist etwas Schreckliches passiert	I need you for an ernomously important mission ⚔ 🛡<br>---<br>Something terrible has happened
	Was denn?<br>Ach wirklich?	What?<br>Oh really?
	Es geht um meine Tochter Ann. Sie ist alles, was ich habe. Bald soll sie die Geschicke unseres Volkes in die Hand nehmen.	It concerns my daughter, Ann.<br>She is everything I have. Soon, she is to take the reins of our people's destiny.
	Verstehe, sie ist also die einzige Thronfolgerin?	I understand. So, she is the sole heir to the throne?

Appendix A - Conversational Scripts

	-	-		-	-		Genau. Schau her, das ist sie.<br>---<br>$systemLinkedMediaObject	Exactly. Look here, this is her.<br>---<br>$systemLinkedMediaObject
	-	-		-	-		Und was ist mit Ann genau passiert?:no<br>Deine Tochter? Die Haarfarbe sagt mir was anderes.:yes	And what happpened to Ann exactly?: no<br>Your daughter? The hair color tells me something different.: yes
	-	-		-	-	*Condition "yes"*	Ach, meine Haare sind gefärbt. Kommt besser. 😉	Oh, my hair is dyed. Looks better. 😉
	-	-		-	-		OK. Und was ist mit Ann nun genau passiert?	OK. And what exactly happened to Ann?
	-	-		-	-		Ann wurde vor drei Tagen brutal von unserem Erzfeind Ash entführt.<br>---<br>Ash wird Ann erhängen und danach in vier Stücke teilen, sollten wir nicht bis zum Sonnenuntergang kapitulieren.	Ann was brutally kidnapped three days ago by our archenermy Ash.<br>---<br>Ash will hang Ann and then cut her into four pieces if we don't surrender by sunset.
	-	-		-	-		Das hört sich nicht gut an. Aber was hat das mit mir zu tun?	That doesn't sound good. But what does that have to do with me?"
	-	-		-	-		Ich benötige deine Hilfe um Ann zu befreien.	I need your help to free Ann.
	-	-		-	-		Verstehe. Was bietest du, wenn ich dir helfe?<br>Ich kann helfen. Aber das wird teuer.	I understand. What do you offer if I help? I can help. But that will be expensive.
	-	-		-	-		Du und deine Familie bekommen Speis und Trank bis zu eurem Lebensende.	You and your family will be provided with food and drink until the end of your lives."
	-	-		-	-		OK, ich bin bereit deine Ann zu befreien.:yes<br>Das wird nicht einfach, aber ich bin bereit es zu tun.:yes<br>Da muss mehr drin sein.:no	"OK, I'm ready to free your Ann. :yes It won't be easy, but I'm willing to do it. :yes There must be something more to it. :no"
	-	-		-	-	*Condition "no"*	Nun gut...<br>---<br>Deine Kinder erhalten zudem die beste Ausbildung, die wir anbieten können. So werden sie später das Schicksal unseres Volkes im Rat der Weisen zusammen mit Ann mitbestimmen.	In addition, your children will receive the best education we can offer. This way, they will later have a say in shaping the fate of our people alongside Ann in the Council of the Wise."
	-	-		-	-		In Ordnung $coachName. Auf dieses Angebot lasse ich mich ein.	"Alright, $coachName. I'll accept this offer.
**§participantName**	Es freut mich dich kennenzulernen.<br>Wie darf ich dich nennen?	I am glad to get to know you.<br>How should I call you?	**§participantName**	Cool, dass wir uns hier kennenlernen. Wie soll ich dich denn nennen?	Cool that we meet each other here. How should I call you?	**§participantName**	Dann haben wir eine Abmachung. Wie darf ich dich eigentlich nennen?	Then we have a deal. How should I address you?
	____	____		____	____		Nenn mich einfach____.	Just call me ____.
	Ist $participantName korrekt?	Is $participantName correct?		Ist $participantName korrekt?	Is $participantName correct?		OK, $participantName. Ist der Name so korrekt?	OK, $participantName. Is the name correct?
	Ja: yes<br>Nein, ich würde den Namen gerne anpassen: no	Yes: yes<br>No, I would like to change the name: no		Ja 👍 :no<br>Nein, ich würde den Namen gerne anpassen:yes	Yes 👍 :no<br>No, I would like to change the name:yes		Ja, das passt.:yes<br>Nein, gib mir nochmals einen Versuch.:yes	Yes, that's correct: yes<br>No, give me one more try.: no
Condition "no"	Gerne kannst du den Namen nochmals anpassen.	Of course you can still change the name.	*Condition "no"*	Gerne kannst du den Namen nochmals anpassen.	Of course you can still change the name.	*Condition "no"*	OK, noch ein Versuch. Wie ist dein Name?	OK, one more try. What is your name?
	__	____		__	____		Nenn mich _ .	Call me _.
Condition "yes"	Danke, $participantName.<br>---<br>Ich bin ein Chatbot und wir können miteinander chatten	Thank you, $participantName.<br>---<br>I am a chatbot and we can chat with each other	*Condition "yes"*	Danke, $participantName. Let's start!<br>---<br>Ich bin ein Chatbot und wir können miteinander chatten	Thank you, $participantName. Let's start!<br>---<br>I am a chatbot and we can chat with each other	*Condition "yes"*	Danke, $participantName. Nun hör mir gut zu.	Thank you, $participantName. Now listen to me carefully.
	Aha - Chatbots kenne ich.<br>Ah, spannend! Kannst du mir mehr über dich erzählen?	Aha - I know Chatbots.<br>Ah, interesting! Can you tell me more about yourself?		Aha - Chatbots kenne ich.<br>Ah, spannend! Kannst du mir mehr über dich erzählen?	Aha - I know Chatbots.<br>Ah, interesting! Can you tell me more about yourself?		Ich höre.<br>Gerne.	I am listening.<br>Gladly.
	Ich bestehe aus ein wenig künstlicher Intelligenz und viel harter Arbeit.	I am made of a little artificial intelligence and a lot of hard work.		Ich bestehe aus ein wenig künstlicher Intelligenz und viel harter Arbeit.	I am made of a little artificial intelligence and a lot of hard work.		Meine Späher haben berichtet, dass Ann auf die Insel Tokko-Takka-Tikki gebracht wurde.<br>---<br>Hast du schon einmal davon gehört?	My scouts reported that Ann was taken to the island of Tokko-Takka-Tikki.<br>---<br>Have you heard about it before?
	-	-		Ist schon heftig wie krass die Wissenschaft heute ist, oder? 😍	It is amazing how cool the science is today, isn't it? 😍		-	-
	Wer bist du wirklich?	Who are you exactly?		really cool!	really cool!		Ja, klar. Bei starkem Rückenwind ist die Insel nur einen halben Seetag entfernt.:yes<br>Nein, wo ist das?:no	Yes, clear. The island is only half a sea day away with strong tailwind.: yes<br>No, where is it?:no
	Das ist eine berechtigte Frage.<br>---<br>Ich bin wie ein Gesundheitsexperte, der dir Tipps gibt, wie du mit Stress umgehen kannst.	This is a good question.<br>---<br>I am like some health expert, offering you valuable tips on managing stress.		Also: wenn du schon immer mal einen Coach gegen Stress ausprobieren wolltest, bist du hier am richtigen Ort. 😊	So, if you've always wanted to try a coach for stress, you're in the right place.	*Condition "no"*	Bei starkem Rückenwind ist die Insel einen halben Seetag entfernt. Hier ist die Karte.<br>---<br>$systemLinkedMediaObject	When there is a strong tailwind, the island is half a day's sail away. Here is the map.<br>---<br>$systemLinkedMediaObject
	-	-		-	-	*Condition "yes"*	Richtig. Ich habe unser Ziel auf der Karte markiert.<br>---<br>$systemLinkedMediaObject	Correct, I have marked our destination on the map.<br>---<br>$systemLinkedMediaObject
	Ich bin gespannt.<br>Das klingt aufregend.	I am curious.<br>That sounds exciting.		-	-		Danke.	Thanks.

Appendix A - Conversational Scripts

	Beginnen wir einmal mit den Grundlagen.<br>---<br>Wenn du zum Beispiel eine Situation als nicht bewältigbar wahrnimmst, wird eine körperliche und psychische Reaktion bei dir ausgelöst.<br>---<br>Diese Reaktion nennt sich Stress.	Let's begin with the fundamentals.<br>---<br>For instance, when you perceive a situation as overwhelming, it elicits a physiological and psychological response within you.<br>---<br>This response is commonly known as stress.		Beginnen wir mal mit den Grundlagen.<br>---<br>Wenn du zum Beispiel eine Situation als nicht bewältigbar wahrnimmst, wird eine körperliche und psychische Reaktion bei dir ausgelöst.<br>---<br>Diese Reaktion nennt sich Stress	Let's begin with the fundamentals.<br>---<br>For instance, when you perceive a situation as overwhelming, it elicits a physiological and psychological response within you.<br>---<br>This response is commonly known as stress.		Lass uns beide zunächst Ann aus den Klauen von Ash befreien. Wir werden uns später an ihm rächen!!! ⚔️	Let's first rescue Ann from the clutches of Ash together. We will seek revenge on him later!!! ⚔️
	Ja, das macht Sinn.<br>Das wusste ich schon.	Yes, that makes sense.<br>I already knew that.		Ja, das macht Sinn.<br>Das wusste ich schon.	Yes, that makes sense.<br>I already knew that.		Um Ann zu befreien, benötigen wir noch ein Segelboot und jede Menge Rückenwind?	To set Ann free, we still need a sailing boat and some tailwind?
	Und natürlich gibt's dafür viele Gründe.<br>---<br>Zu den häufig genannten "Stressoren" zählen Zeitdruck, Schlafmangel, soziale Konflikte und persönliche Probleme.	And naturally, there are various factors that contribute to stress.<br>--<br>Some commonly mentioned stressors include time constraints, lack of sleep, interpersonal conflicts, and personal issues.		Und natürlich gibt's dafür viele Gründe.<br>---<br>Dazu zählen zum Beispiel Zeitdruck, Schlafmangel, soziale Konflikte und persönliche Probleme.	And naturally, there are various factors that contribute to stress.<br>--<br>Some commonly mentioned stressors include time constraints, lack of sleep, interpersonal conflicts, and personal issues.		Das Boot ist gleich unten am Hafen, aber Wind...<br>---<br>... wir haben seit Tagen Windstille.	"The boat is down at the harbor, but the wind...<br><br>...we've had calm weather for days."
	Was kann man dagegen tun?<br>Ich verstehe. Und nun?	What can be done about it?<br>I understand. And now?		Was kann man dagegen tun?<br>Das kenne ich und nun?	What can be done about it?<br>I understand. And now?		Verdammt! Die verdammte Windstille.	Damn! The damned calmness of the wind.
**Health literacy information on stress**	Zunächst möchte ich von dir wissen, wie du auf alltägliche Stress-Situationen reagierst.<br>---<br>Neigst du dazu im Stress besonders viel zu Essen, z.B. zwei Schokoladentafeln auf einmal?	Next I would like to know, how you react to stressful situations in your daily life.<br>---<br>Do you tend to eat a lot, too, when you're stressed, like, for example, eating two chocolate bars at once?	**Health literacy information on stress**	Also ich esse einfach mal so 2 Tafeln Schokolade. 🍫 🍫<br>---<br>Machst du das auch?	So, I just casually eat about 2 bars of chocolate. 🍫 🍫<br><br>Do you do that too? "		-	-
	Ja, aber ich würde gerne darauf verzichten.:A<br>Ja, ist mir schon passiert.:B<br>Nein, ich mache andere Sachen, wenn ich gestresst bin.:C	Yes, but I would like to stop doing it.: A<br>Yes, it happened to me.:B<br>No, I do other things when I am stressed out.: C		Ja, aber ich find's nicht geil. :A<br>Ja, ist mir schon passiert. Ich liebe Schokolade! :B<br>Nein, ich mache andere Sachen, wenn ich gestresst bin.:C	Yes, but I don't think it is cool. :A<br>Yes, it has already happened to me. I love chocolate! :B<br>No, I do something else when I am stressed.: C		-	-
Condition A	Gut, die Einstellung stimmt schon mal!	Good, the attitute is already right!	*Condition A*	Yes, die Einstellung stimmt schon mal!	Yes, the attitude is already right!		-	-
Condition B	Diese Reaktion ist leider nicht sehr nachhaltig.	Unfortunately, this kind of reaction is not so sustain	*Condition B*	Oh Fuck dann geht es dir ja gleich wie mir!<br>Ich möchte aber echt damit aufhören.	Oh Fuck then you're the same as me!<br>But I really want to stop.		-	-
Condition C	Machst du noch was anderes?	Do you do something else?	*Condition C*	Mhmm, was macht du dann?	Mhmm, what do you do then?		-	-
	Ich game ein paar Stunden. :A<br>Ich atme tief ein und aus und gehe an die frische Luft. :B<br>Ich trinke Bier oder sonst einen Drink oder rauche Zigaretten. :C<br>Ich mache gar nichts. :D<br>Ich mache etwas ganz anderes. :E	I play games for a couple of hours: A<br>I breath in and out deeply and go out into fresh air: B<br>I drink beer or any other drinks, or smoke cigarettes: C<br>I don't do anything: D<br>I do something completely different: E		Ich game ein paar Stunden. :A<br>Ich atme tief ein und aus. Und gehe an die frische Luft. :B<br>Ich trinke Bier oder sonst einen Drink oder rauche Zigaretten. :C<br>Ich mache gar nichts. :D<br>Ich mache etwas ganz anderes. :E	I play games for a couple of hours: A<br>I breath in and out deeply and go out into fresh air: B<br>I drink beer or any other alcoholic drinks, or smoke cigarettes: C<br>I don't do anything: D<br>I do something completely different: E		-	-
Condition A/C	Diese Reaktion ist nicht sehr gut für deine Gesundheit. Wir lernen gleich viel bessere Strategien kennen.	This kind of reaction is not so good for your health. We are about to learn much better strategies	*Condition A/C*	Diese Reaktion ist nicht sehr gut für deine Gesundheit.	This kind of reaction is not so good for your health.		-	-
Condition B	Perfekt, Atmung und Bewegung sind gut für dich - insbesondere in Stress-Situationen.	Perfect, breathing and movement are good for you - especially in stressful situations	*Condition B*	Perfekt, Atmung und Bewegung sind gut für dich - insbesondere in Stresssituationen.	Perfect, breathing and movement are good for you - especially in stressful situations		-	-
Condition D	Okay.	Okay	*Condition D*	Okay	Okay		-	-
Condition E	Gut zu wissen.	Good to know	*Condition E*	Okay gut zu wissen.	Okay good to know.		-	-
**Breathing Exercise Preparation & Start**	So, jetzt möchte ich dir eine Strategie zum Umgang mit Stress vorstellen, ein 5-Minuten Atemtraining.	So, now I'd like to introduce you to a strategy for dealing with stress, a 5-minute breathing training.	**Breathing Exercise Preparation & Start**	So, ich habe eine perfekte Lösung für mich bei Stress entdeckt. Und zwar, ...<br>---<br>---<br>ein 5-Minuten Atemtraining	So I have discovered a perfect solution for me when I am stressed. Namely...<br>---<br>---<br>a 5min breathing training	**Breathing Exercise Preparation & Start**	Wieso? Ich habe gehört, dass du mit deiner Sprache und Atmung selbst bei Windstille ein Segelboot in Bewegung bringen kannst.<br>---<br>Nur so können wir Ann heute bis zum Sonnenuntergang befreien.	Why? I have heard that you can make a sailboat move with your speech and breath even when there is no wind.<br>---<br>That's the only way we can free Ann by sunset today.
	Erzähl mir mehr, $coachName.<br>Ich bin gespannt.	Tell me more, $coachName.<br>I am curious.		Erzähl mir mehr, $coachName.<br>Das ist aber interessant.	Tell me more, $coachName.<br>This is interesting.		Diesen Skill habe ich schon lange nicht mehr eingesetzt. Bin mir nicht sicher, wie das genau geht.	I haven't used that skill in a long time. I'm not sure exactly how it works.
	Du brauchst absolut nichts ausser Motivation.<br>Lass uns das Training gleich starten.	You don't need anything besides your motivation.<br>Let's start the training right away.		Du brauchst absolut nichts ausser Motivation.	You don't need anything besides motivation.		Ich werde dir helfen, auch wenn du schon lange kein Atemtraining mehr durchgeführt hast.	I will help you even if you have not done any breathing training for a long while.
				Okay, ich bin bereit.	Okay, I am ready.		Alles klar.	All clear.
	Um das Maximum aus dem Atemtraining herauszuholen, muss ich wissen, wie lange du für 3 "langsame" Atemzüge brauchst. Bist du dabei?	In order to maximize the benefits of the breathing exercise, I need to know how long it takes you to complete 3 "slow" breaths. Are you in?		Um das Maximum aus dem Atemtraining herauszuholen, muss ich wissen, wie lange du für 3 "langsame" Atemzüge brauchst. Bist du dabei?	In order to maximize the benefits of the breathing exercise, I need to know how long it takes you to complete 3 "slow" breaths. Are you in?		Um das Boot mit deiner Atmung auf Maximum zu beschleunigen, muss ich wissen, wie lange du für 3 langsame Atemzüge benötigst. Bist du dabei?	In order to maximize the boat speed, I need to know how long it takes you to complete 3 "slow" breaths. Are you in?
	Ja, ich bin dabei.<br>Ne du, gib mir das 08/15 Atemtraining.	Yes, I am in.<br>Nah, give me that run-of-the-mill breathing exercise.:no"		💪 Aber sowas von!:yes<br>❌ Ne du, gib mir das 08/15 Atemtraining.:no	" 💪 Absolutely!:yes<br>❌ Nah, give me that run-of-the-mill breathing exercise.:no"		💪 klar!:yes<br>❌ Nein, das brauche ich nicht.:no	💪 sure!:yes<br>❌ No, I don't need that.:no

Appendix A - Conversational Scripts

Condition "yes"	OK, top. Wir starten mit einer Trockenübung.<br>---<br>Atme dazu nun 3 Mal langsam ein und aus. Achte bitte darauf, dass du durch die Nase einatmest und durch den Mund ausatmest. Versuche beim Ausatmen die Lippen etwas zusammen zu pressen.<br>---<br>Starte bitte jetzt mit den 3 langsamen Atemzügen und gebe mir Bescheid, wenn du fertig bist.	Ok, top. We will start with a dry run.<br>---<br>Take 3 slow breaths, making sure to inhale through your nose and exhale through your mouth. Try gently pursing your lips while exhaling.<br>---<br>Now please start with 3 slow breaths and let me know when you are done.
	OK, ich atme nun 3 Mal langsam durch die Nase ein und den Mund aus und zwar ab ... jetzt.	Alright, I will now take three slow breaths, inhaling through my nose and exhaling through my mouth, starting from... now
	Warte 1	*Wait 1*
	... ok, du machst das gut ...	ok, you are doing well
	Warte 2	*Wait 2*
	Ich bin fertig.	I am ready
	Wie fühlst du dich im Moment?	How do you feel at the moment?
	überhaupt nicht gut:1<br>nicht gut:2<br>eher nicht gut:3<br>eher gut:4<br>gut:5<br>voll und ganz gut:6	Not good at all: 1<br>Not good:2<br>Rather not good: 3<br>Rather good: 4<br>Good: 5<br>Fully good: 6
	Danke für deine Antwort.<br>---<br>Lass uns beginnen, $participantName!	Thank you for your response.<br>--<br>Let's get started, $participantName!
**After breathing exercise**	Wie fühlst du dich im Moment?	How do you feel at the moment?
	überhaupt nicht gut:1<br>nicht gut:2<br>eher nicht gut:3<br>eher gut:4<br>gut:5<br>voll und ganz gut:6	Not good at all: 1<br>Not good:2<br>Rather not good: 3<br>Rather good: 4<br>Good: 5<br>Fully good: 6
	Besten Dank für deine Antwort.<br>---<br>Herzlichen Glückwunsch. An dieser Stelle endet die Demo-Version der StressLess App.	Thank you for your response.<br>---<br>Congratulations! At this point, the demo version of the StressLess app comes to an end.
	Aber wie geht es weiter?	But what happens next?
	Mit einer 3-Minuten Umfrage kannst du jetzt die Zukunft der App mitgestalten und - Du kriegst 2 EUR zum Schluss.<br>---<br>Bist du dabei?	With a 3min survey you can now help design the future of the app and - You will receive 2 USD at the end.<br>---<br>Are you in?
	Aber klar doch, Ehrensache.: yes<br>Na gut. Ich bin dabei.: yes<br>Nein, ich möchte das Studienteam nicht unterstützen.: no	But of course, matter of honor.: yes<br>All right. I'm in.: yes<br>No, I do not want to support the study team.: no
Condition "yes"	Vielen Dank, $participantName.<br>Dann klicke bitte nun auf die folgende Schaltfläche um die Umfrage zu starten und die Vergütung zu erhalten.	Thank you so much, $participantName.<br>Then just click on the following button to start the survey and get the compensation.
Condition "no"	Alles klar, $participantName. Vielen Dank für deine Teilnahme.<br>---<br>Falls du doch noch die Zukunft von StressLess mitgestalten und, sofern du magst, eine kleine Vergütung zu erhalten, dann kannst du einfach auf die folgende Schaltfläche klicken.<br>---<br>Nochmals herzlichen Dank und bis dann 👋	Alright, $participantName. Thank you so much for participantion.<br>---<br>In case you still want to shape the future of StressLess, if you like, get a small compensation, then please simply click on the following button.<br>---<br>Thank you again and see you then 👋
	Deeplink to Survey	*Deeplink to Survey*

Condition "yes"	OK, top. Wir starten mit einer Trockenübung.<br>---<br>Atme dazu nun 3 Mal langsam ein und aus. Achte bitte darauf, dass du durch die Nase 👃 einatmest und durch den Mund 👄 ausatmest. Versuche beim Ausatmen die Lippen etwas zusammen zu pressen.<br>---<br>Starte bitte jetzt mit den3 langsamen Atemzügen	Ok, top. We will start with a dry run.<br>---<br>Take 3 slow breaths, making sure to inhale through your nose and exhale through your mouth. Try gently pursing your lips while exhaling.<br>---<br>Now please start with 3 slow breaths and let me know when you are done.
	OK, ich atme nun 3 Mal langsam durch die Nase 👃 ein und den Mund 👄 aus und zwar ab ... jetzt 👊	Alright, I will now take three slow breaths, inhaling through my nose 👃 and exhaling through my mouth 👄 starting from... now 👊
	Warte 1	*Wait 1*
	... ok, du machst das gut ...	ok, you are doing well
	Warte 2	*Wait 2*
	BAM 💥 ich bin fertig.	BAM 💥 I am ready.
	Wie fühlst du dich im Moment?	How do you feel at the moment?
	überhaupt nicht gut:1<br>nicht gut:2<br>eher nicht gut:3<br>eher gut:4<br>gut:5<br>voll und ganz gut:6	Not good at all: 1<br>Not good:2<br>Rather not good: 3<br>Rather good: 4<br>Good: 5<br>Fully good: 6
	Danke für deine Antwort & jetzt:<br>---<br>Machen wir uns bereit $participantName! 🔥 🔥 👏	Thank you for your answer and now:<br>---<br>Let's get ready to rumble $participantName! 🔥 🔥 👏
	Los geht's!	Let's go!
**After breathing exercise**	Wie fühlst du dich im Moment?	How do you feel at the moment?
	überhaupt nicht gut:1<br>nicht gut:2<br>eher nicht gut:3<br>eher gut:4<br>gut:5<br>voll und ganz gut:6	Not good at all: 1<br>Not good:2<br>Rather not good: 3<br>Rather good: 4<br>Good: 5<br>Fully good: 6
	Besten Dank für deine Antwort.<br>---<br>Herzlichen Glückwunsch. 💪 An dieser Stelle endet die Demo-Version der Breeze App.	Thank you for your response.<br>---<br>Congratulations! At this point, the demo version of the Breeze app comes to an end.
	Aber wie geht es weiter?	But what happens next?
	Mit einer 3-Minuten Umfrage kannst du jetzt die Zukunft der App mitgestalten und - Du kriegst 2 EUR zum Schluss.<br>---<br>Bist du dabei?	With a 3min survey you can now help design the future of the app and - You will receive 2 USD at the end.<br>---<br>Are you in?
	Aber klar doch, Ehrensache.: yes<br>Na gut. Ich bin dabei.: yes<br>Nein, ich möchte das Studienteam nicht unterstützen.: no	But of course, matter of honor.: yes<br>All right. I'm in.: yes<br>No, I do not want to support the study team.: no
Condition "yes"	Vielen Dank, $participantName.<br>Dann klicke bitte nun auf die folgende Schaltfläche um die Umfrage zu starten und die Vergütung zu erhalten.	Thank you so much, $participantName.<br>Then just click on the following button to start the survey and get the compensation.
Condition "no"	Alles klar, $participantName. Vielen Dank für deine Teilnahme.<br>---<br>Falls du doch noch die Zukunft von Breeze mitgestalten und, sofern du magst, eine kleine Vergütung zu erhalten, dann kannst du einfach auf die folgende Schaltfläche klicken.<br>---<br>Nochmals herzlichen Dank und bis dann 👋	Alright, $participantName. Thank you so much for participating.<br>--<br>If you would like to continue shape the future of Breeze and receive a small compensation, please click the button below and answer a few questions in the following survey.<br><br>Once again, thank you, and we'll see you soon! 👋
	Deeplink to Survey	*Deeplink to Survey*

Condition "yes"	OK, top. Wir starten mit einer Trockenübung.<br>---<br>Atme dazu nun 3 Mal langsam ein und aus. Achte bitte darauf, dass du durch die Nase einatmest und durch den Mund ausatmest. Versuche beim Ausatmen die Lippen etwas zusammen zu pressen.<br>---<br>Starte bitte jetzt mit den 3 langsamen Atemzügen und gebe mir Bescheid, wenn du fertig bist.	Ok, top. We will start with a dry run.<br>---<br>Take 3 slow breaths, making sure to inhale through your nose and exhale through your mouth. Try gently pursing your lips while exhaling.<br>---<br>Now please start with 3 slow breaths and let me know when you are done.
	OK, ich atme nun 3 Mal langsam durch die Nase ein und den Mund aus und zwar ab ... jetzt.	Alright, I will now take three slow breaths, inhaling through my nose 👃 and exhaling through my mouth 👄 starting from... now 👊
	Warte 1	*Wait 1*
	... ok, du machst das gut ...	ok, you are doing well
	Warte 2	*Wait 2*
	Ich bin fertig.	I am ready
	Wie fühlst du dich im Moment?	How do you feel at the moment?
	überhaupt nicht gut:1<br>nicht gut:2<br>eher nicht gut:3<br>eher gut:4<br>gut:5<br>voll und ganz gut:6	Not good at all: 1<br>Not good:2<br>Rather not good: 3<br>Rather good: 4<br>Good: 5<br>Fully good: 6
	Dann lass uns aufbrechen...	Then let's set off...
	Ich bin bereit ⚔️	I am ready ⚔️
**After breathing exercise**	Wie fühlst du dich im Moment?	How do you feel at the moment?
	überhaupt nicht gut:1<br>nicht gut:2<br>eher nicht gut:3<br>eher gut:4<br>gut:5<br>voll und ganz gut:6	Not good at all: 1<br>Not good:2<br>Rather not good: 3<br>Rather good: 4<br>Good: 5<br>Fully good: 6
	Besten Dank für deine Antwort.<br>---<br>Und herzlichen Glückwunsch. ⚔️ An dieser Stelle endet die Demo-Version der Tragic Kingdom App.	Thank you for your response.<br>---<br>Congratulations! ⚔️ At this point, the demo version of the TragicKingdom app comes to an end.
	Aber wie geht es weiter?	But what happens next?
	Mit einer 3-Minuten Umfrage kannst du jetzt die Zukunft der App mitgestalten und - Du kriegst 2 EUR zum Schluss.<br>---<br>Bist du dabei?	With a 3min survey you can now help design the future of the app and - You will receive 2 USD at the end.<br>---<br>Are you in?
	Aber klar doch, Ehrensache.: yes<br>Na gut. Ich bin dabei.: yes<br>Nein, ich möchte das Studienteam nicht unterstützen.: no	But of course, matter of honor.: yes<br>All right. I'm in.: yes<br>No, I do not want to support the study team.: no
Condition "yes"	Vielen Dank, $participantName.<br>Dann klicke bitte nun auf die folgende Schaltfläche um die Umfrage zu starten und die Vergütung zu erhalten.	Thank you so much, $participantName.<br>Then just click on the following button to start the survey and get the compensation.
Condition "no"	Alles klar, $participantName. Vielen Dank für deine Teilnahme.<br>---<br>Falls du doch noch die Zukunft von Breeze mitgestalten und, sofern du magst, eine kleine Vergütung zu erhalten, dann kannst du einfach auf die folgende Schaltfläche klicken.<br>---<br>Nochmals herzlichen Dank und bis dann 👋	Alright, $participantName. Thank you so much for participating.<br>--<br>If you would like to continue shape the future of TragicKingdom and receive a small compensation, please click the button below and answer a few questions in the following survey.<br><br>Once again, thank you, and we'll see you soon! 👋
	Deeplink to Survey	*Deeplink to Survey*

A2 Detailed information on milestones

Download: During the streams, the individuals watching the stream could click on the advertisement link in the chat or scan a QR-code on a banner, which was linked directly to the designated app version. The participants downloaded, installed, and used the apps on their personal mobile phones.

M0 – Choose Coach: When participants opened the app for the first time, participants could choose to interact with either a male or female version of the chatbot character.

M1 – Admin and Consent: After downloading the mobile app, the intervention started with a short administration and consent dialogue. The participants were informed about the terms and conditions and were asked (1) whether they met the inclusion criteria; (2) whether they agreed with the terms and conditions (Appendix 1A); and (3) whether they allowed access to the microphone (iOS and Android), photographs (only Android), and media (only Android) on their devices. The acceptance of all these criteria was necessary to continue the intervention.

M2 – Welcome and Nickname: Afterwards, the chatbot welcomed the users and asked for their preferred nickname.

M3 – End of Introductory Dialogue: In TragicKingdom, the starting dialogue focused on the description of the situation and the assignment of the story. In StressLess and Breeze, the chatbot shared some information and asked questions about stress and stress handling. The chatbot also reacted to the answers and gave a brief comment on how the answer was perceived by the chatbot. The information exchange, as well as the questions, were not included in TragicKingdom. Overall, this dialogue led up to the introduction of the breathing training.

M4 – End of Breathing Training Preparation (= Start of Actual Breathing Training): Next, the chatbot led the participant through the preparation (including a voluntary pre-exercise) and the introduction of the breathing training. The chatbot asked the participant to choose between a predefined or personalized setting for the training. Generally, the exercise in all three app versions followed the same breathing pattern of 4-1-5 that is considered standard as described by Russell et al. [1]. However, the "Breaths per Minute" could be personalized if the participants chose this option [1].

- *Predefined*: If a participant chose the predefined pace, it was fixed to five Breaths per Minute
- *Personalized:* If the participant chose the personalization option, a short exercise to personalize the training was conducted together with the chatbot. The personal pace for the training was then calculated based on how long it took the participant to complete three deep breaths during the chat.

Afterwards, the slow-breathing training opened within the app. Before the start of the training, participants received a short tutorial which explained that good posture is recommended, how the breathing technique works, and how long the exercise is supposed to last. It also advised participants to conduct the training with headphones, and it explained the goal of the exercise. Participants had the option to review the tutorial at any time. The actual breathing training was then initiated with three voice commands (i.e., StressLess: "I sit relaxed!", "I am ready!", "Start the exercise!"; Breeze and TragicKingdom: "Lift the anchor!", "Set the sail!", "Let's start today's journey!") and a countdown from three to zero.

M5 – End of Breathing Training: The breathing exercise lasted a maximum of five minutes, but study participants could terminate the exercise at any time without specifying reasons or any implications for the remaining parts of the intervention. The breathing training ended either automatically after the five minutes with another three voice commands (i.e., StressLess: "I breathy normally again", "I am ready", "Finish the exercise!; Breeze and TragicKingdom: "Set the anchor!", "Haul in the sail!", "I have finished today's journey!") or, when a participant terminated the exercise earlier, without the three voice commands.

M6 – Intervention Completion: After completing the breathing training, the chatbot informed participants that the app trial was finished, and they were asked to answer some questions on how they feel, and to leave feedback in the final survey. At this point, the lottery mechanism was introduced and the chatbot communicated that all participants who would complete the survey in the next step survey could enter a lottery to win 50 CHF/EUR. This information was deliberately not displayed earlier in the intervention, as it was solely used to motivate participants to complete the final survey.

M7 – Survey Completion & Lottery: After the training was finished, study participants were asked to complete a survey. The survey link was directly integrated into the dialogue with the chatbot, and participants completed the

survey within the app (the survey items are elaborated in the Measurements section). At the end of the survey, the participants could enter their email address to participate in the lottery. After finishing the survey, the participants were advised to delete the app. After the study period concluded, 10 winners were drawn, and the prize was transferred.

A3 Screenshots of the design features in the interventions

In the following, please find screenshots of the translated version. Screenshots of the original version in German used in the study are available upon reasonable request from the corresponding author of the paper.

A3.1 Selection conversational agent (Alexander vs. Alexandra)

StressLess | Breeze | TragicKingdom

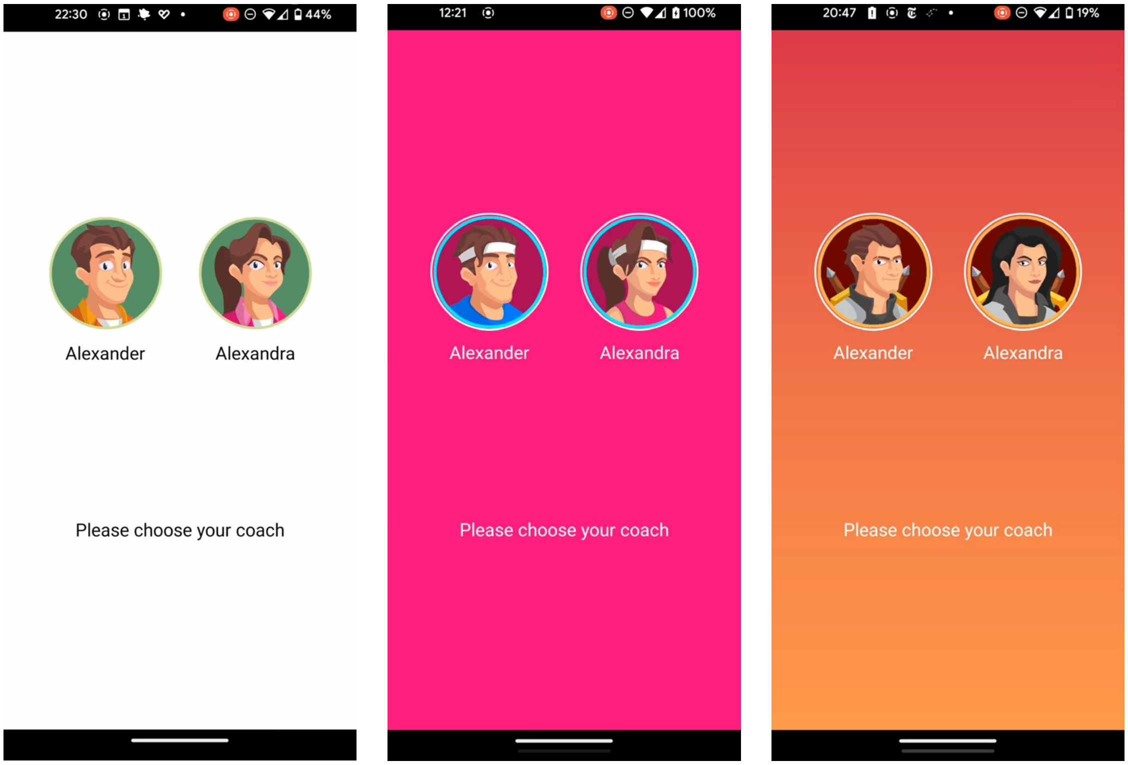

A3.2 *Welcome screen*

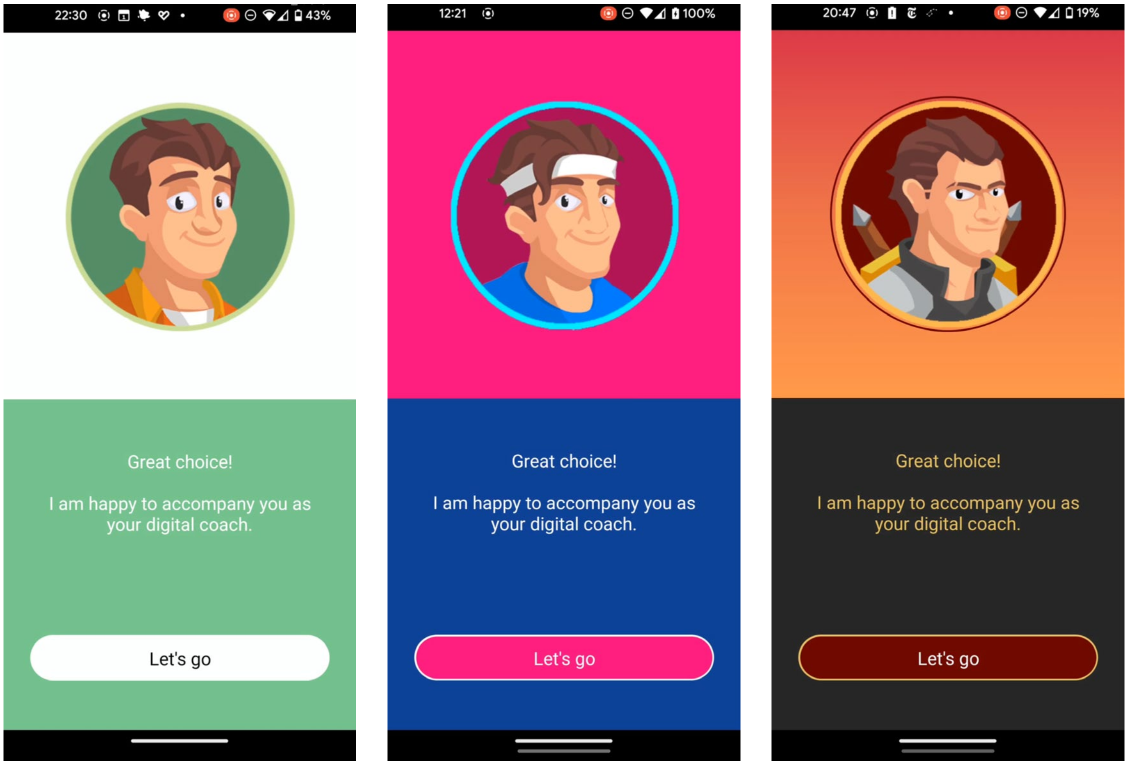

A3.3 Admin and consent dialogue

STRESSLESS BREEZE TRAGICKINGDOM

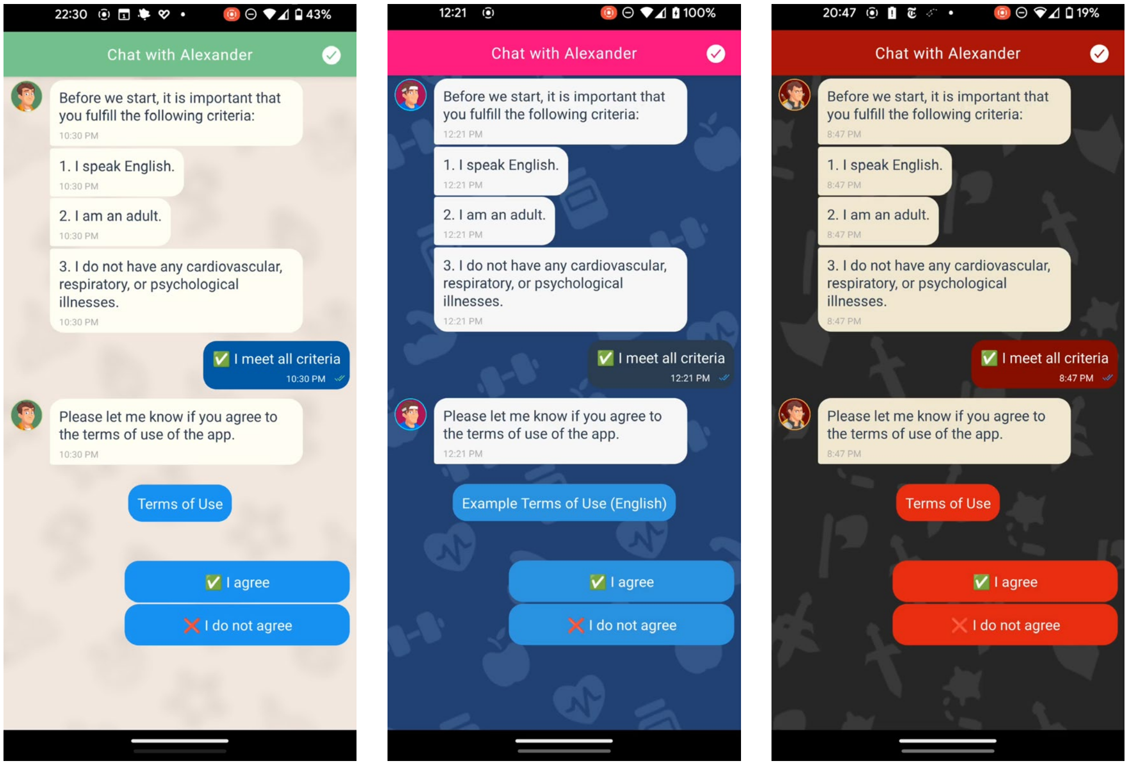

A3.4 Exemplary chatbot interactions

A3.4.1 StressLess

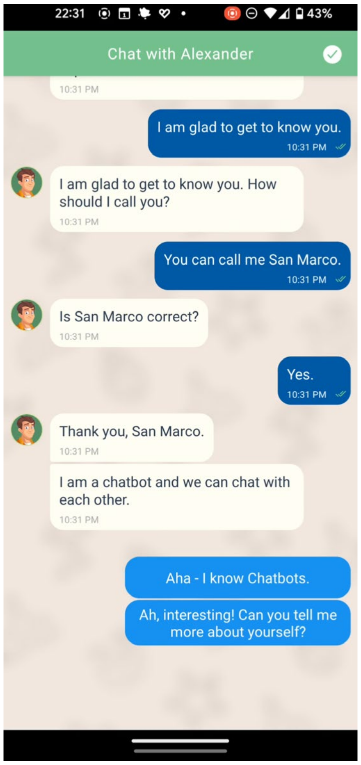

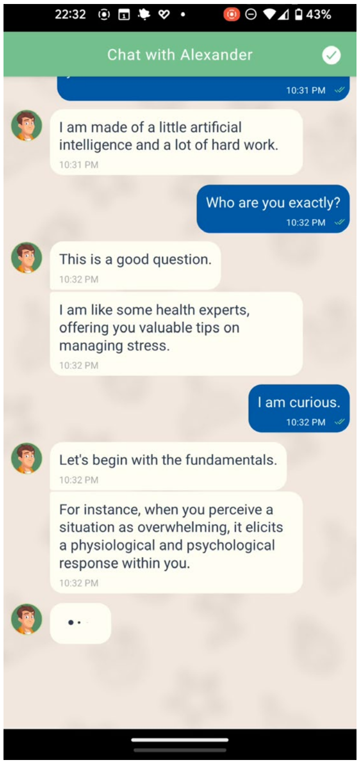

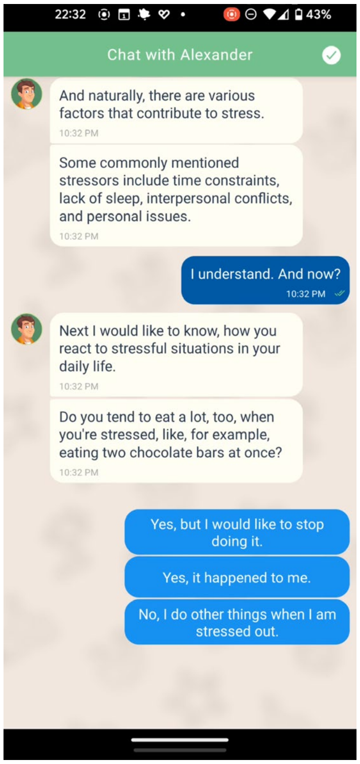

A3.4.2 Breeze

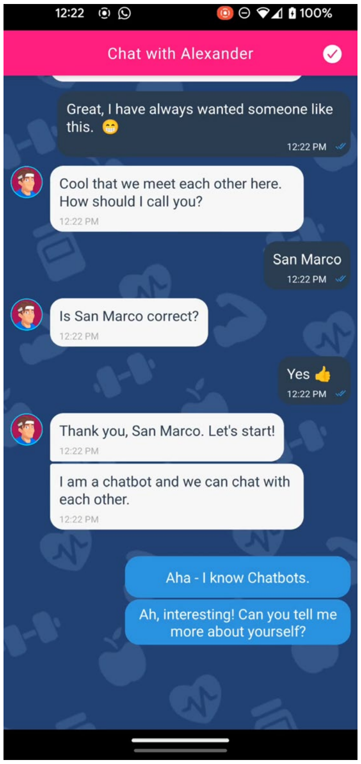

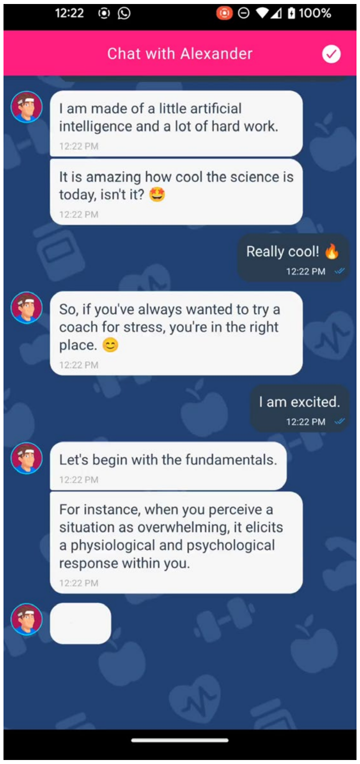

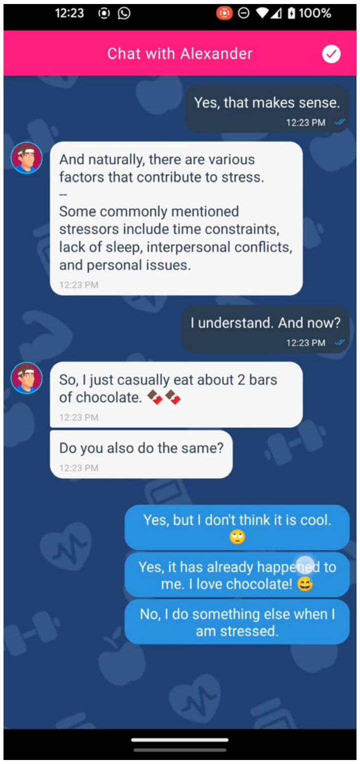

A3.4.3 TRAGICKINGDOM

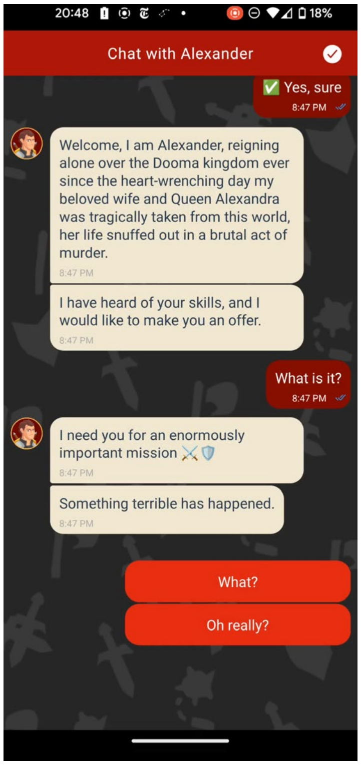

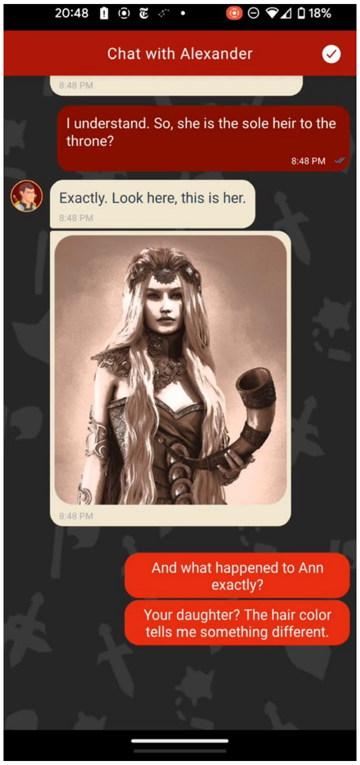

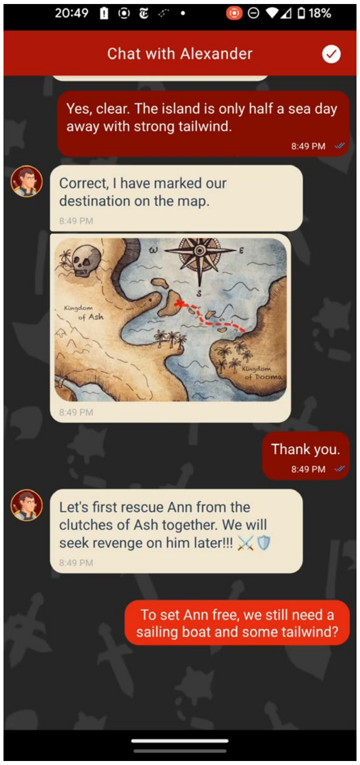

A3.5 Additional features (exemplary for StressLess)

Exemplary screenshots of additional features (i.e., terms of use, microphone access, and personalization of breathing training for StressLess, but they were included in every intervention.

Terms of Use

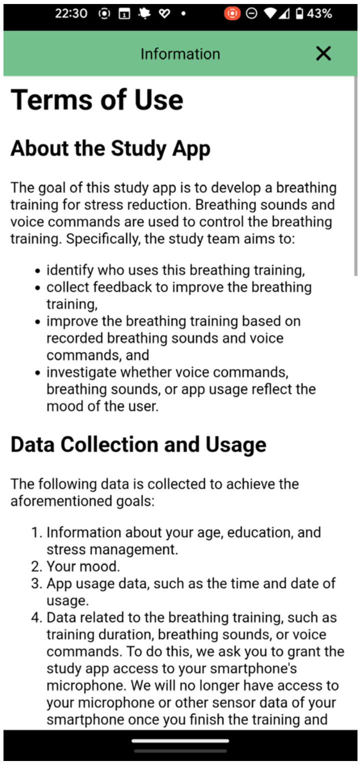

Microphone Access

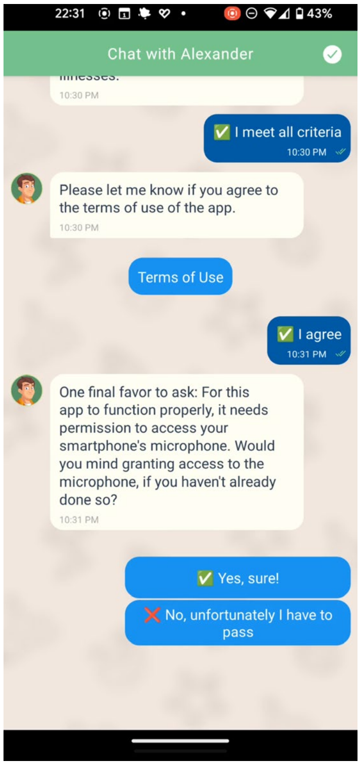

Personalized Breathing Training [1]

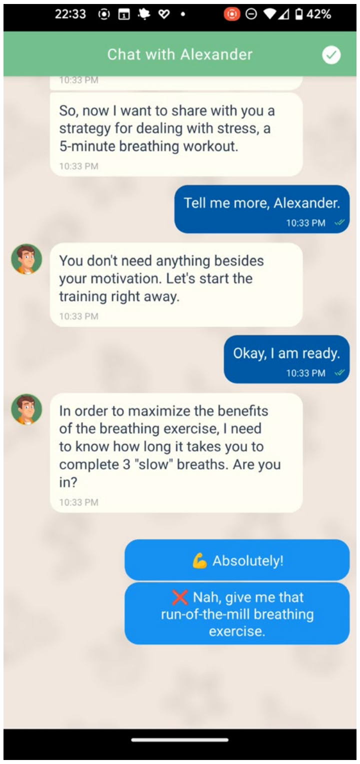

[1] **Personalization Breaths per Minute (Exercise)**

If the participant chose the personalized option for the breathing training, a short exercise within the chat requests the participant to take three deep breaths. Mobile Coach then divides the three breaths resulting in breaths per minutes (BPM). Based on the calculated BPM of the individual, the BPM of the breathing exercise were determined.

#	Measured BPM (Partici-pant)	BPM in the Breathing Training
0	BPM < 5.25	5
1	>= 5.25 BPM < 5.75	5.5
2	>= 5.75 BPM < 6.25	6
3	>= 6.25 BPM < 6.75	6.5
4	>= 6.75 BPM < 7.50	7
5	>= 7.50 BPM < 8.50	8
6	BPM > 8.50	9

Studies of Lehrer et al. (2000, pp. 177-189) and Russell et al. (2017, pp. 362-364) suggested 5.5. to 6 BPM in SPBT. The pre-defined setting was 5 BPM. The breathing pattern was also adapted to the personalization, whereas the pause (1 second) remains constant and the breathing in and out was linearly adapted to the BPM (e.g., 7 BPM → 3.37-1-4.21)

A3.6 Breathing exercise - introductory slides (exemplary for BREEZE)

Posture

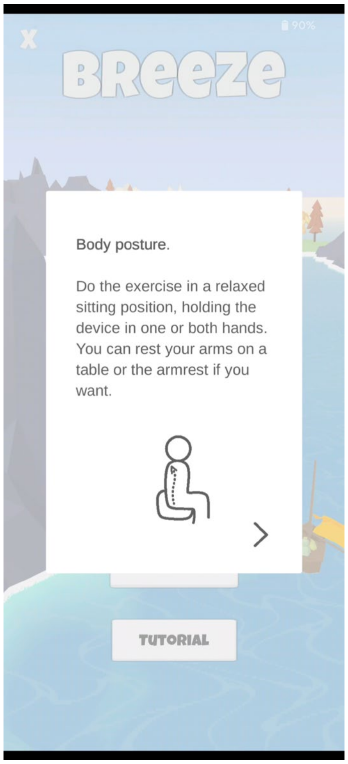

Breathing Technique

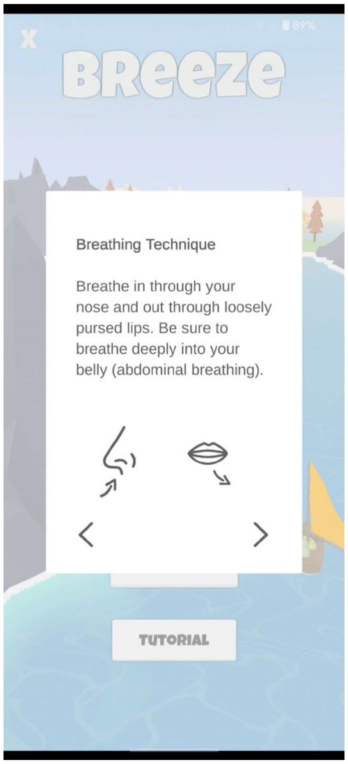

Training duration

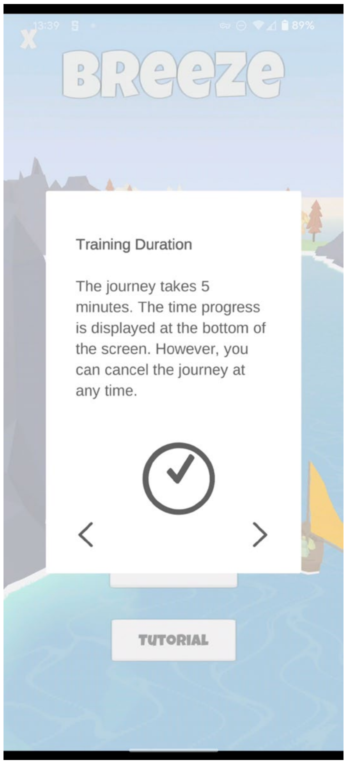

Headphones

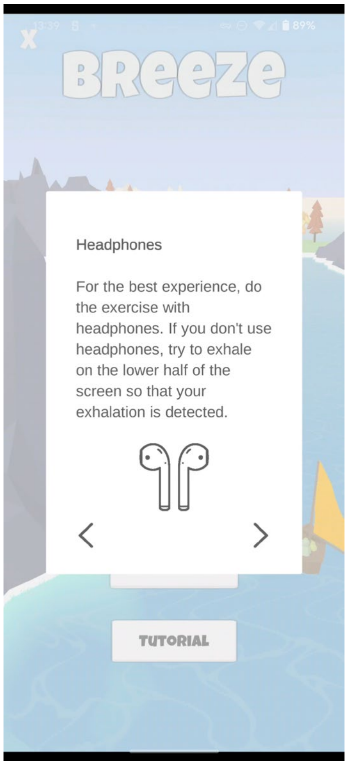

Goal

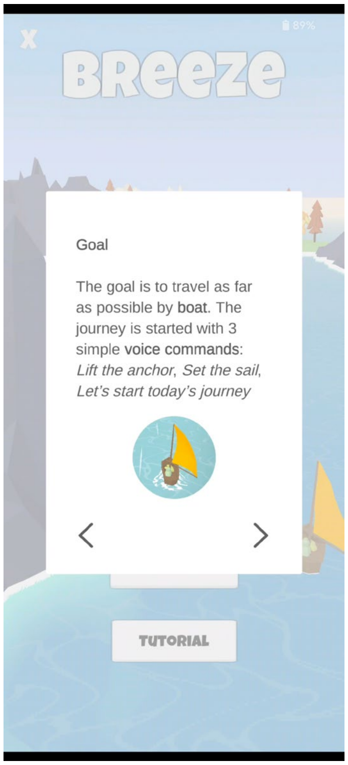

Instructions

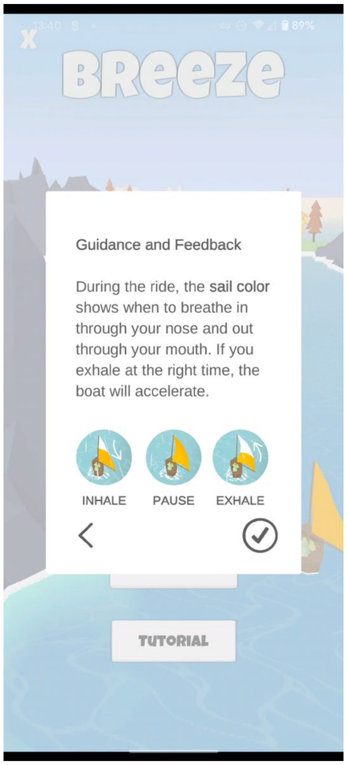

Appendix B RECRUIT influencers

B1 Material for influencers

The material for the streamers consisted of two parts: (1) Leading principles and relevant information for the streamer, (2) a script as inspiration for the spoken advertisement during the stream, (3) banners and QR codes for their stream, and (4) links to promotional videos to be shown during their streams.

B1.1 Leading principles and information for the streamers

German (Original)	English (translated)
«Grundsätzlich ist Folgendes zu beachten:	***"Please note the following basics:***
1. Dies ist eines der ersten Male, dass es so etwas auf Twitch gibt. Es ist vor allem eine Chance für die User, ein neuartiges App-basiertes Atemtraining mitzugestalten	*1. This is one of the first times something like this has existed on Twitch. Above all, it is an opportunity for users to help shape a new type of app-based breathing training*
2. Es fallen keine Kosten für das App-Testing an	*2. There are no costs for the app testing*
3. Auch wenn für eine erfolgreiche Teilnahme Geldpreise zu gewinnen sind, darf dies zu keinem Zeitpunkt bei der Promo der Beta-App erwähnt werden. Dies würde zu einer Verfälschung der Testergebnisse führen.	*3. Even if there are cash prizes to be won for successful participation, this must not be mentioned at any time when promoting the beta app. This would lead to a falsification of the test results.*
4. Regelmässiges auffordern, die Beta-App zu installieren	*4. Regular requests to install the beta app*
5. Falls die Beta-App nicht funktioniert, liegt es immer an der App und nie am Nutzer! Es kann z.B. am Alter des Handys oder dem Internetzugang liegen (es handelt sich um eine frühe Beta-Version, welche noch nicht mit vielen Smartphones getestet wurde)	*5. If the beta app does not work, it is always due to the app and never to the user! For example, it may be due to the age of the cell phone or the internet access (it is an early beta version that has not yet been tested with many smartphones)*
6. Die Entwicklung und dieser Test werden von der ETH Zürich (Uni in der Schweiz) verantwortet»	*6. The development and this test are supported by ETH Zurich (University in Switzerland)"*

B1.2 Scripts for the streamers

The scripts served as a guide and inspiration for the streamers. However, all streamers were allowed to adapt it to their individual style. Minor deltas between the advertising scripts for each app were implemented. The colors illustrate the deltas:

- *Blue = Name of the app,*
- *Green = Explanation of the breathing training (only BREEZE and TRAGICKINGDOM)*
- *Purple = Introduction to storyline (only TRAGICKINGDOM)*

German (original)	English (translated)
**Anfang des Streams**<br>Der/die Streamer:in startet seinen/ihren Stream und wartet, bis die Zuschauer auch live sind. Er/sie begrüßt die Zuschauer und spricht die Beta-App an. Hierbei zeigt er/sie das Promo-Video auf Vimeo. Ebenfalls kann er/sie die App von seinem Handy in die Kamera halten und zeigen, dass er/sie diese ebenfalls heruntergeladen hat. Der Banner erscheint beim Starten des eigentlichen Streams.	**Start of the stream**<br>The streamer starts his/her stream and waits until the viewers are also live. He/she welcomes the viewers and addresses the beta app. He/she shows the promo video on Vimeo. He/she can also hold the app from his/her cell phone up to the camera and show that he/she has also downloaded it. The banner appears when the actual stream starts.
**STRESSLESS**<br>▪ *"Ich hoffe, euch geht es allen gut und ihr hattet ein schönes Wochenende?*<br>▪ *Schaut euch mal die neuen Banner von mir an, es geht um ein neuartiges Atemtraining, welches euch helfen kann, mit Stress besser umzugehen. Das Atemtraining der STRESSLESS App wird aktuell noch entwickelt – und zwar von einem Team der ETH in Zürich, einer Uni in der Schweiz. Heute habt ihr bei mir die Möglichkeit, über den Link eine frühe Version der App zu testen und mit eurem Feedback aktiv mitzugestalten.*<br>▪ *Wichtig ist dabei, dass ich euch nichts verkaufen möchte.*<br>▪ *Ich zeige euch jetzt auch kurz, wie die App STRESSLESS und das Atemtraining aussehen.»*	**STRESSLESS**<br>▪ *"I hope you are all well and had a nice weekend?*<br>▪ *Take a look at the new banners from me, it's about a new kind of breathing training that can help you deal with stress better. The STRESSLESS app breathing training is currently still being developed by a team at ETH Zurich, a university in Switzerland. Today you have the opportunity to test an early version of the app via the link and actively help shape it with your feedback.*<br>▪ *It's important to note that I don't want to sell you anything.*<br>▪ *I'll also briefly show you what the STRESSLESS app and breathing training look like."*
**BREEZE**<br>▪ *"Ich hoffe, euch geht es allen gut und ihr hattet ein schönes Wochenende?*<br>▪ *Schaut euch mal die neuen Banner von mir an, es geht um eine neuartiges Atemtraining, welches euch helfen kann, mit Stress besser umzugehen.*<br>▪ *Das Besondere an der BREEZE App ist, dass ihr mit eurer Ausatmung Wind erzeugt und damit ein Segelboot beschleunigt. Macht ihr das besonders gut, d.h. atmet ihr schön langsam ein und aus, reist ihr besonders weit. Das Atemtraining via App wird aktuell noch entwickelt – und zwar von einem Team der ETH in Zürich, einer Uni in der Schweiz. Heute habt ihr bei mir die Möglichkeit, über den Link eine frühe Version der App zu testen und mit eurem Feedback aktiv mitzugestalten. Wichtig ist dabei, dass ich euch nichts verkaufen möchte.*<br>▪ *Ich zeige euch jetzt auch kurz, wie die BREEZE App und das Atemtraining mit dem Segelboot aussehen."*	**BREEZE**<br>▪ *"I hope you are all well and had a nice weekend?*<br>▪ *Take a look at the new banner I've made, it's about a new kind of breathing training that can help you deal with stress better.*<br>▪ *The special thing about the BREEZE app is that you create wind with your exhalation and thus accelerate a sailing boat. If you do this particularly well, i.e. breathe in and out slowly, you will travel particularly far. The breathing training app is currently being developed by a team at the ETH in Zurich, a university in Switzerland. Today you have the opportunity to test an early version of the app via the link and actively help shape it with your feedback. It's important to note that I don't want to sell you anything.*<br>▪ *I'll also briefly show you what the BREEZE app and breathing training with the sailing boat look like."*
**TRAGICKINGDOM**<br>▪ *"Ich hoffe, euch geht es allen gut und ihr hattet ein schönes Wochenende? Schaut euch mal die neuen Banner von mir an, es geht um eine neuartiges*	**TRAGICKINGDOM**<br>▪ *"I hope you are all well and had a nice weekend? Take a look at the new banners from me, it's about a new*

Atemtraining, welches euch helfen kann, mit Stress besser umzugehen.<br>▪ *Das Besondere an der TragicKingdom App ist, dass ihr mit eurer Ausatmung Wind erzeugt und damit ein Segelboot beschleunigt. Macht ihr das besonders gut, d.h. atmet ihr schön langsam ein und aus, reist ihr besonders weit. Zudem könnt ihr nur so Ann aus den Klauen der Truppen von Ash befreien. Wer Ann und Ash ist, fragt ihr euch? Nun, das seht ihr in der App. Mehr möchte ich dazu nicht sagen. Das Atemtraining via App wird aktuell noch entwickelt – und zwar von einem Team der ETH in Zürich, einer Uni in der Schweiz. Heute habt ihr bei mir die Möglichkeit, über den Link eine frühe Version der App zu testen und mit eurem Feedback aktiv mitzugestalten. Wichtig ist dabei, dass ich euch nichts verkaufen möchte.*<br>▪ *Ich zeige euch jetzt auch kurz, wie die TragicKingdom App und das Atemtraining mit dem Segelboot aussehen.»*	*kind of breathing training that can help you deal with stress better.*<br>▪ *The special thing about the TragicKingdom app is that you create wind with your exhalation and thus accelerate a sailing boat. If you do this particularly well, i.e. breathe in and out slowly, you will travel particularly far. This is also the only way to free Ann from the clutches of Ash's troops. Who are Ann and Ash, you ask? Well, you can see that in the app. That's all I want to say about it. The breathing training app is still being developed by a team at the ETH in Zurich, a university in Switzerland. Today you have the opportunity to test an early version of the app via the link and actively help shape it with your feedback. It's important to note that I don't want to sell you anything.*<br>▪ *I'll also briefly show you what the TragicKingdom app and the breathing training with the sailing boat look like."*
Zeigen der Chat-Interaktion & Atemtraining via Promo-Video. Zusätzlich soll im Live-Stream gezeigt werden, dass die App installiert wurde («Vertrauensbeweis»). Zudem soll erwähnt werden, dass die Beta-App Internet-Zugang benötigt, besonders gut bei neueren Smartphones funktioniert und ggf. abstürzen kann, da es sich um eine frühe Version handelt. Nebenbemerkung für den Streamer (nicht für die Audience): «Erwartungshaltung muss klar kommuniziert werden», d.h. nicht zu «viel verkaufen», es handelt sich um eine «seriöse» Studie der ETH Zürich ;-)	Showing the chat interaction & breathing training via promo video. In addition, the live stream should show that the app has been installed ("proof of trust"). It should also be mentioned that the beta app requires internet access, works particularly well on newer smartphones and may crash as it is an early version. Side note for the streamer (not for the audience): "Expectations must be clearly communicated", i.e. don't "sell too much", this is a "serious" study by ETH Zurich ;-).
**Hauptteil des Streams**<br>Regelmäßig spricht der/die Streamer:in die Zuschauer auf den Banner und den QR Code an. Er/sie erklärt, um was es geht und zeigt, dass er/sie die Beta-App auch auf seinem/ihrem Handy installiert hat. (Wichtig, da so die Angst genommen werden kann, «Vertrauensbeweis» der Audience gegenüber)<br>▪ *"Um nochmals auf meine neuen Banner zurückzukommen. Ich habe mich entschieden, die Beta-App StressLess / Breeze/ TragicKingdom auch zu testen und mein Feedback zu geben. Warum, fragt ihr euch? Nun, da das Atemtraining mit dem Segelboot helfen kann, mit Stress besser umzugehen.*	**Main part of the stream**<br>The streamer regularly addresses viewers about the banner and the QR code. He/she explains what it is about and shows that he/she has also installed the beta app on his/her cell phone. (Important, as this can take away the fear: "proof of trust" towards the audience)<br>▪ *"To come back to my new banners. I have decided to test the StressLess / Breeze / TragicKingdom beta app and give my feedback. Why, you ask? Well, because breathing training with the sailboat can help you deal with stress better.*
**Abschluss Stream**<br>**StressLess:**<br>▪ *"Zum Schluss möchte ich euch nochmals motivieren, die Beta-App StressLess herunterzuladen. Ihr habt die einmalige Möglichkeit diesen ersten Prototyp mit Early Access zu testen und euer Feedback zu geben. Ladet euch StressLess runter, es ist spannend so etwas zu sehen und auszuprobieren. Manchmal ist eine bewusste Atmung einfach sehr hilfreich, um mit Stress besser umzugehen."*<br>***Breeze/TragicKingdom:***<br>▪ *"Zum Schluss möchte ich euch nochmals motivieren, die Beta-App Breeze/TragicKingdom herunterzuladen. Ihr habt die einmalige Möglichkeit diesen ersten*	**End of the Stream**<br>**StressLess:**<br>▪ *"Finally, I would like to motivate you once again to download the StressLess beta app. You have the one-time opportunity to test this first prototype with Early Access and give your feedback. Download StressLess, it's exciting to see and try something like this. Sometimes conscious breathing is simply very helpful in dealing with stress better."*<br>**Breeze/TragicKingdom:**<br>▪ *"Finally, I would like to motivate you once again to download the Breeze/TragicKingdom beta app. You have the one-time opportunity to test this first*

Prototyp mit Early Access zu testen und euer Feedback zu geben. Ladet euch B*REEZE*/T*RAGIC*K*INGDOM* *runter, lasst euch auf die Story ein! Es ist spannend so etwas mit dem Segelboot zu sehen und auszuprobieren. Manchmal ist eine bewusste Atmung sehr hilfreich, um mit Stress besser umzugehen.“*<br><br>Zeigen der Chat-Interaktion & Atemtraining via Promo-Video (oder live auf dem Smartphone), falls die Chat-Interaktion und das Atemtraining noch nicht gezeigt wurden.	*prototype with Early Access and give your feedback. Download* B*REEZE*/ T*RAGIC*K*INGDOM* *and get involved in the story! It's exciting to see and try out something like this with a sailing boat. Sometimes conscious breathing is very helpful to deal with stress better.”*<br>Showing the chat interaction & breathing training via promo video (or live on the smartphone) if the chat interaction and breathing training have not yet been shown.

B1.3 Banner with QR-Code

The agency developed and designed banners for the streamers to display during their advertisement stream. The banners were animated starting with the app icon, a short teaser text and the QR-Code. The QR-Code was generated based on the deep link allocated to each streamer.

B1.3.1 Condition 1 (Stress Less)

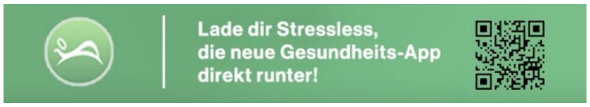

English translation: "Download StressLess, the new health app directly!"

B1.3.2 Condition 2 (Breeze)

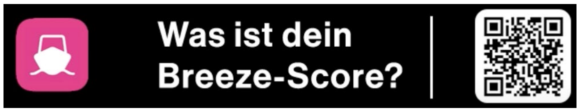

English Translation: "What is your Breeze score?"

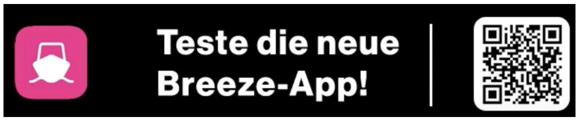

English translation: "Test the new Breeze app!"

B1.3.3 Condition 3 (TragicKingdom)

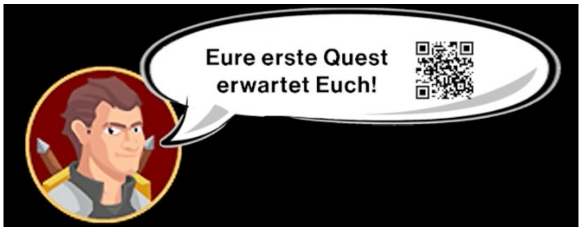

English translation: "Your first quest awaits you!"

B1.4 Promotional videos

	DE (Original)	EN (translated)
**StressLess**	https://vimeo.com/837158930/74565d3863	https://vimeo.com/837139601/ad85957ee0
**Breeze**	https://vimeo.com/837139994/0c826db946	https://vimeo.com/835904989/6b6947ce13
**TragicKingdom**	https://vimeo.com/837159967/23d8667a47	https://vimeo.com/836451745/9f5323376c

Appendix C REACH participants

C1 Impressions of the advertisement streams

Each streamer realized the advertising in a personal and individual style. The provided materials were used also quite differently. The section illustrates some examples of the streams.

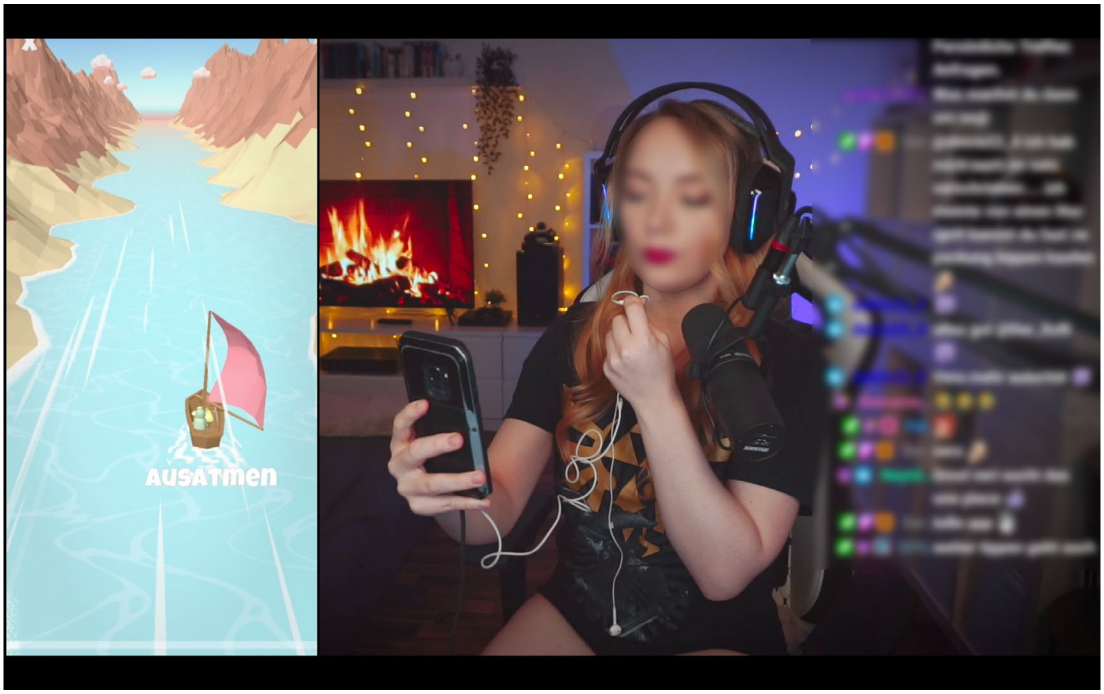

Streamer ID1 directly projected the smartphones screen to the stream, directly illustrating the app to the audience. As the chat displays the audience was reacting to the app.

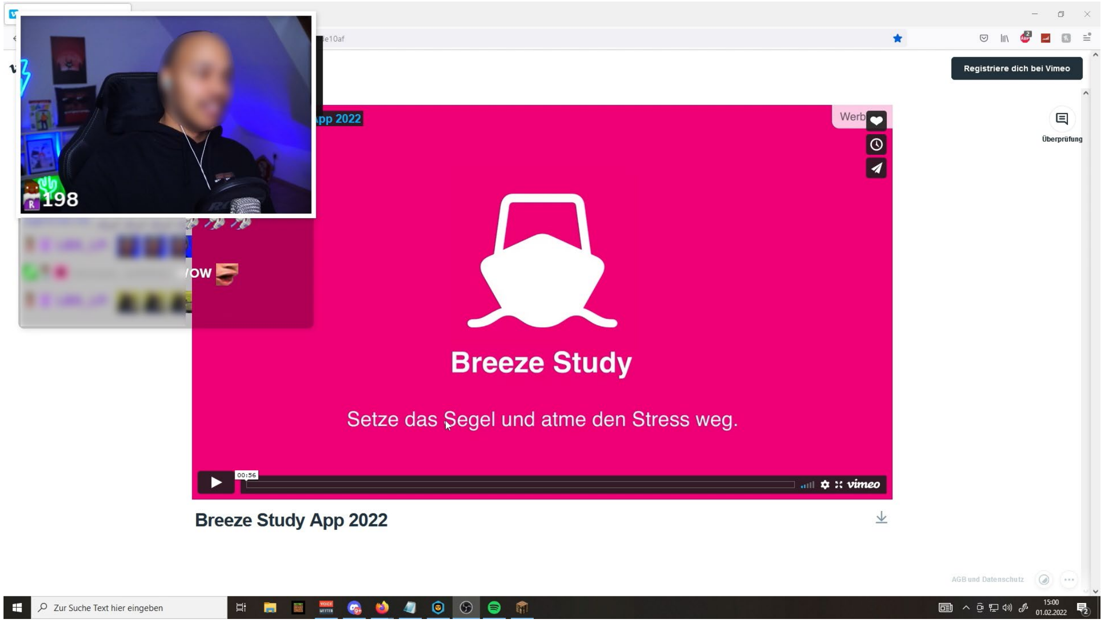

Streamer ID2 shortly before showing the promotion video. The streamer shared the screen with the screen with the audience.

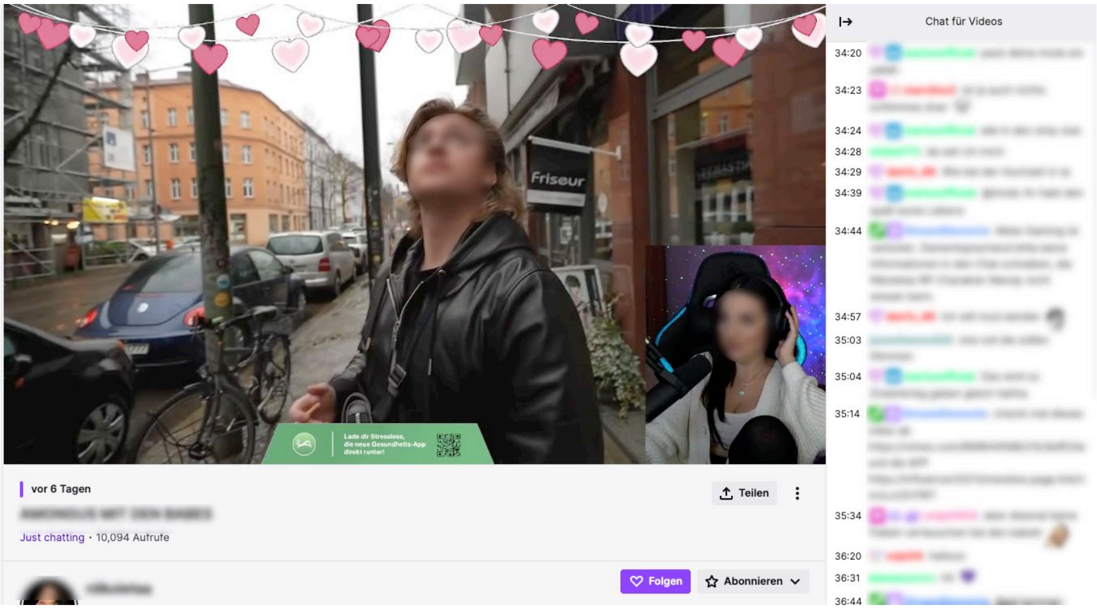

Streamer ID3 reacted to other videos while displaying the banner for the advertisment. In the chat "StreamElements" posted messages with the direct links to the promotion videos and the apps.

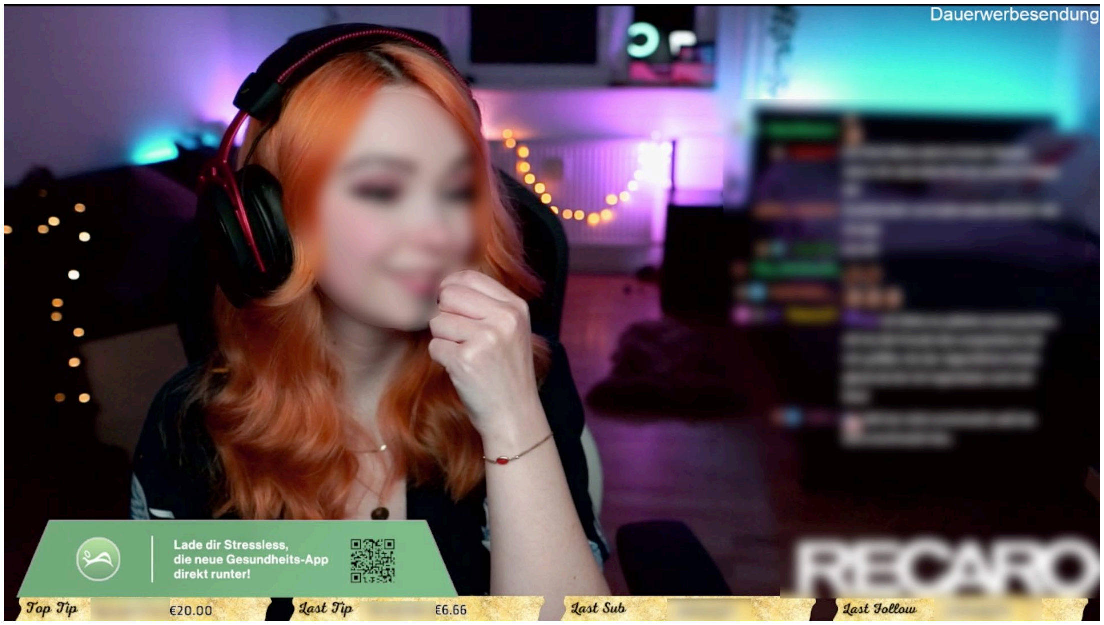

Streamer ID4 talking with the audience about random topics. In the bottom left of the video, the banner of the Stress Less app is displayed. Many streamers used this format to just shortly display the banner while doing something else (gaming, talking etc.) with the audience.

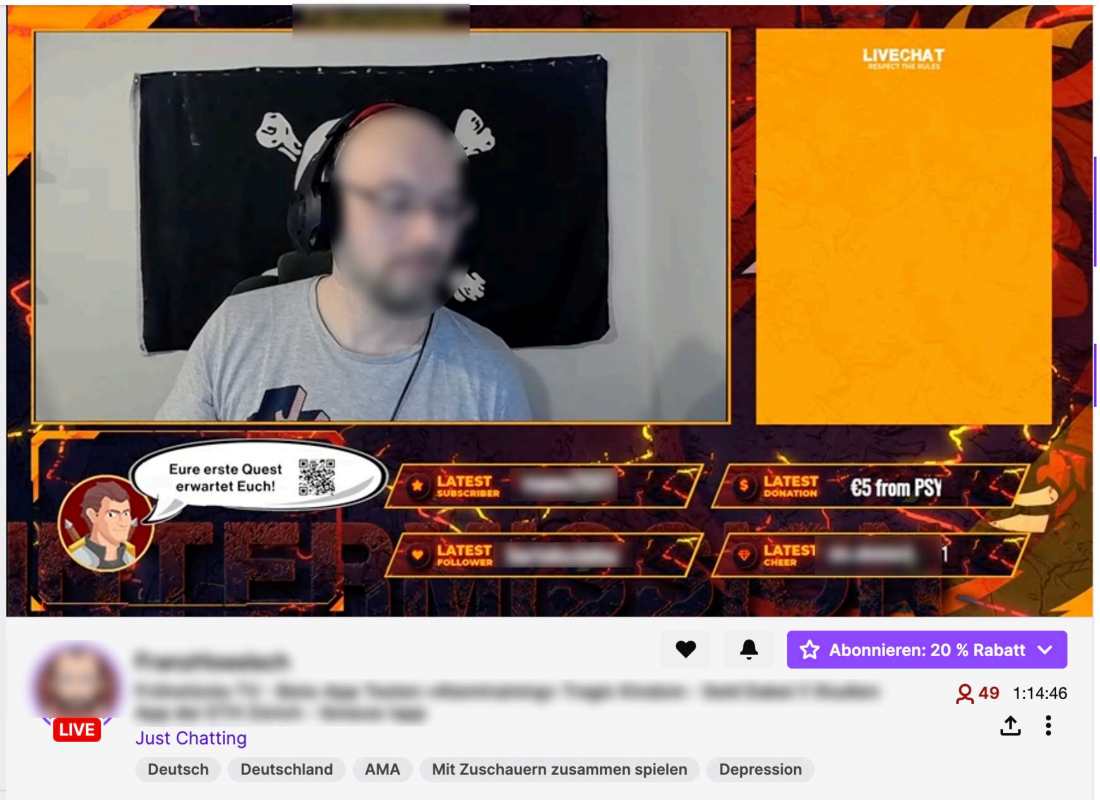

Streamer ID5 talking to the audience in the morning, a pirate flag in the background, and presenting the apps TRAGICKINGDOM with the banner in the bottom left of the video. In this picture also the title of the stream as well as the live chat are illustrated.

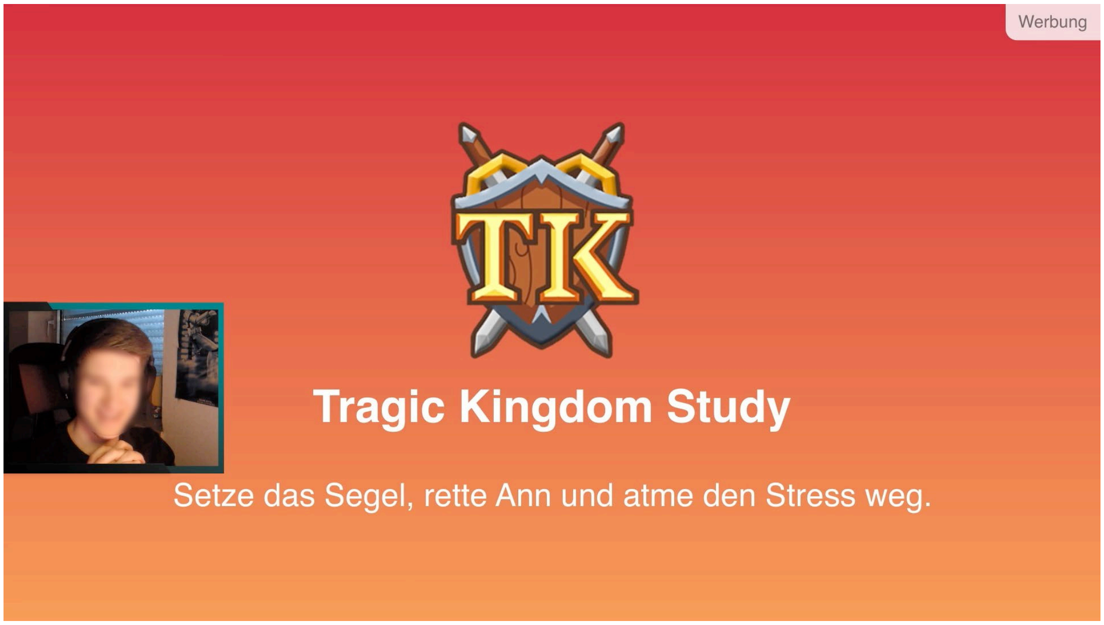

Streamer ID6 showing the promotion video during the stream. The streamer showed the video full screen and added some details.

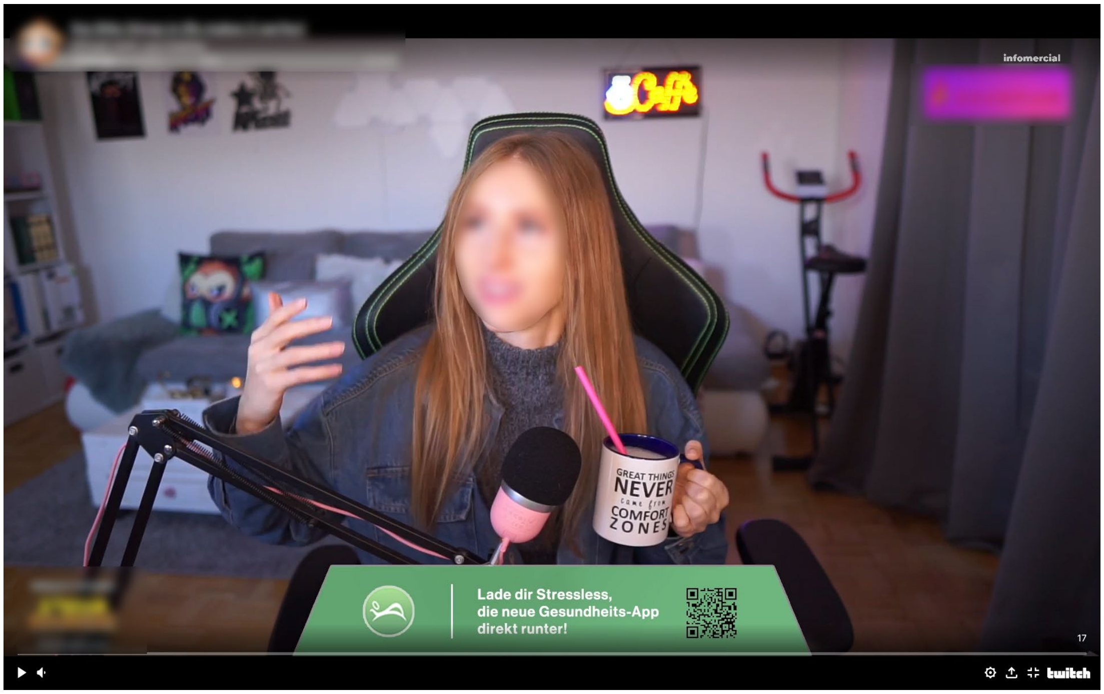

Streamer ID8 displaying the banner during the stream, holding a coffee cup stating: "Great things never come from your comfort zones"

C2 Screenshot of Exemplary Twitchtracker Archival Data

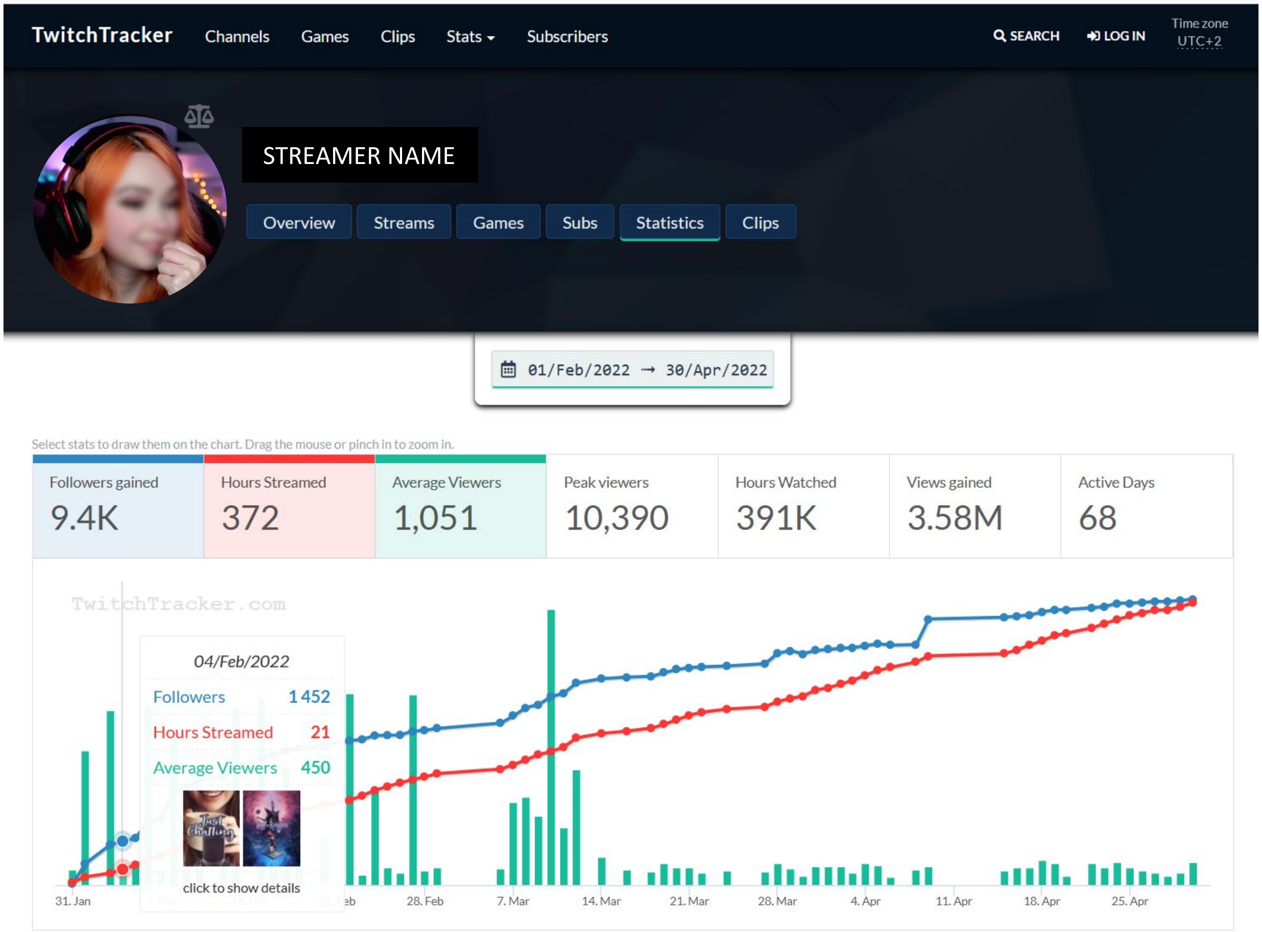

Twitchtracker data for streamer ID4 on February 4th, 2022: Average Viewers 450

C3 Notes on influencer strategy

To give a short overview of the streams, here are some short commentaries to each streamer and their advertisements.

Streamer ID	Comments
ID1	In the first stream, ID1 talks to her audience in general and then redirected the topic towards the advertisement. She then showed the promotion video and commented on it. She then interacted with the chat and talked about the app. Later, she screened mirrored the whole intervention live on the stream. She also integrated the chat during the presentation of the intervention and let the audience choose the gender of the coach or the name. During the entire stream, the banner was displayed. Occasionally the direct link to the app was sent in the chat.<br>The second screen only included the banner and some short references to the banner during the stream.
ID2	ID2 executed the advertisement during a short pause of the game he was playing. He kept the advertisement short and teased the audience to test the app themselves. In the short time he showed the promotion video with personal comments, the banner as well as his phone with the app installed. He also illustrated to the audience how they can download the app within the app store and shared the direct link in the chat. He further emphasized that downloading the app is regarded as direct support towards him as a streamer.
ID3	ID3 did not particularly communicate the advertisement during her stream. In the stream watched other (Youtube) videos and blind reacted to them. In the chat, she sent relatively often messages with the link to the promotion video as well as the deep links to the apps. Besides, the banner was displayed during nearly the whole stream. Between watching to videos, she also briefly mentioned to check out the links sent in the chat. She did not show the promotion video. Later in the stream she played a game with several other streamers, probably increasing her reach.
ID4	In ID4's first stream, she guides the audience towards the topic of stress and presents the intervention as a solution. She also highly engaged with the actions and the questions in the chat. Later, she showed the promotion video and commented it. Afterwards, she displayed the banner with the QR-code and continued the conversation. Unfortunately, she interpreted some information and disclosed that the intervention is only for iOS users, however, she corrected it in the next streams. She had a computer crash during the stream and restarted the stream (that is the reason for two streams on the same day). In the new started stream as well as the stream the following day, she displayed the banner and sometimes made references towards it. She further shared the direct link in the chat.
ID5	In ID5's first stream for the advertisement campaign, he talked about the stress and the intervention. From time to time, he showed the promotion video and added some comment to it. He further showed himself while using the app directly. Over the duration of approximately one hour, he repeated the mentioned steps and motivated the audience to download the app. In the following streams he displayed the banner during the whole steam and occasionally mentioned it. In one stream he took a pause but left the stream on the and the banner displayed. His streams were often very early in the morning, probably being a reason for not to engaging live chats.
ID6	ID6 finished his game and then started the advertisement. He screened mirrored the intervention to the stream and let the audience experience it. While doing so, he interacted with the chat and commented the intervention. Later he showed the promotion video. He displayed the banner not during the stream but included it in his information section on the page. There the banner could be scanned at any time.
ID7	In his first and second stream, ID7 executed the promotion at the beginning of the stream. He declared a strong personal interest in breathing and strongly motivated the audience to try breathing exercises. He even proposed and illustrated breathing exercises beyond the intervention. In the first stream, he showed the promotion video and engaged with the comments in the chat. In the second stream, he also screened mirrored the app to the audience. During the promotion part, he displayed the banner with the QR-code.
ID8	ID8 announced the study already in the description of the stream the advertisements. She started the stream by teasing her two announcements and the later started playing a game. During the stream she then paused the game and advertised the app. She displayed the banner, showed the promotion video, and also motivated the participants directly to try the app so that they can later discuss it. She then tested the app live with the audience, showing the screen from time to time to the camera. She also mentioned additional information about the app e.g., Beta-App, from ETH Zurich etc. Due to a misunderstand the streamer did her first advertisement stream in English.

Appendix D ENGAGE participants

D1 Terms and conditions

The Terms & Conditions were included in the app descriptions within the app stores as well as direct link within the interventions themselves. The participants had to agree to the terms & conditions before starting with the full intervention. The terms and conditions were translated to English for this paper. The original version in German used in the study is available upon reasonable request from the corresponding author of the paper.

About the app
This study and the involved app focus on a slow-paced breathing exercise. This exercise can be used for learning how to manage stress by regulating one's one breath. There are three main goals of this study: (1) to find out who would be more likely to use the developed app, (2) to gather feedback for the improvement of the app, and (3) to improve the slow-paced breathing exercise.

Data Collection and Usage
The following anonymized data, i.e., data that cannot be traced back to the individual user, will be collected and used to better understand who reacts to the advertisements of the app and to improve the involved algorithm for the slow-paced breathing training: (a) some basic information on your background such as level of education and age and some information on how you handle stress, (b) app usage data (e.g. time and date of interactions), (c) data on your interaction with the breathing exercise such as how long you do the exercise or how well you conduct the exercise (for this, we will ask you to give us access to your smartphone's microphone for the duration of exercise, so that we can gather your voice commands and breathing sounds when needed. We will have no further access to your microphone or any other sensor data of your smartphone once you stop the exercise and finalize the study), (d) subjective ratings of questionnaire items. Finally, we will ask you about your email address in case you want to participate in a lottery. We will delete your email address immediately after the prices have been drawn and send out. Any data transfer between the app and the database will be encrypted to ensure privacy and security. We will store all app related data that we collect on General Data Protection Regulation (GDPR)-compliant, i.e., in full accordance with legal requirements, servers in a secure database located in Europe. The access to all data is protected and only accessible for members of the research team of this study. We will delete the nickname that you can choose for interacting with the chatbot. Any audio data which we might gather when you authorize access to your microphone during the breathing exercise will be transmitted to our secure serves over an encrypted connection using state-of-the-art encryption technologies. Upon receiving the data, they will be screened by a member of the research team and any potentially private audio recordings will be immediately and irreversibly deleted. The remaining data are stored on devices of ETH Zurich with protected access so that only members of the research team can access them. The anonymized data may be published in academic journals for research purposes and, according to the Open Science Data initiative by the Swiss National Science Foundation, made available in a non-commercial open database to the general public provided there are no legal, ethical, or other restrictions.

Who is offering the app?
This app is created and offered by a group of independent researchers under the lead of the Center for Digital Health Interventions (https://www.c4dhi.org) at ETH Zurich and the University of St. Gallen, Switzerland. That is, we are not a professional service provider and as a non-profit initiative providing the app free of charge, we must limit any warranty and liability accordingly. The study that embeds the app is funded by the university ETH Zurich, the Centre for Digital Health Interventions (CDHI), and the CSS Health Insurance Company. However, CSS Health Insurance played no role in designing the study, will not have access to the data, and will not be included when analyzing the data.

No Warranty
We do not warrant or guarantee that the intended goals and results of the use of the app will be reached. We assume no warranties, neither express nor implied, regarding the app, its features, content, information, and interactions, including but not limited to warranties of functionality, availability, accuracy, completeness, timeliness, non-infringement of third-party rights or fitness for a particular purpose.

No Liability
The User uses the app on his/her own risk. To the fullest extent permitted by applicable law we do not assume any liability for any direct, indirect, or consequential damages suffered in connection with the provision and use of the app.

Law and Jurisdiction
By using the app, the user accepts that the provision and use of the app is subject to the laws of Switzerland and any disputes arising in connection with the provision and use of the app are subject to the jurisdiction of the courts of the city of Zurich, Switzerland.

Contact
If you have any further questions, please contact the project team.

D2 Measurements

All measurements were translated to English for this paper. The original version in German as used in the study is available upon reasonable request from the corresponding author of the paper.

D2.1 Chatbot-based: mood state questionnaire

Construct/Items	Measurement	Adapted from
**Mood State Questionnaire (MDMQ)**		
How do you feel right now?	*6-point Likert-scale*<br>*1 = not good at all; 6 = completely good*	[2]

D2.2 Survey-based: sex, age, country of residence, general health status

Construct/Items	Measurement	Adapted from
**Sex**		
Your sex?	*Female, Male, No answer, Other: _____*	[3]
**Age**		
How old are you?	*Free numeric input*	*Own item*
**Country of Residence**		
In which country do you live?	*1 Switzerland; 2 Germany; 3 Austria; 4 Principality of Liechtenstein; 5 Other: free text input*	*Own item*
**General Health Status**		
How is your general state of health? It is ...	*5-point Likert-scale:1 very bad; 2 poor*<br>*3 mediocre; 4 good; 5 very good*	[4]

D2.3 Survey-based: socioeconomic status questions (education, occupation, income)

Construct/Items	Measurement	Adapted from
**Education 1 - General**		
What is your highest general educational qualification?	1. *No degree yet (student)*<br>2. *Degree after a maximum of 7 years of schooling (primary school)*<br>3. *General school / secondary modern school / intermediate school*<br>4. *Intermediate school diploma / lower level of gram mar school / secondary school / lower level of AHS*<br>5. *Abitur / subject-specific university entrance qualification / Matura / upper level of AHS*<br>6. *Other school diploma, specifically: ...*	[5]
**Education 2 - Vocational**		
What is your highest vocational qualification?	1. *No degree, still in vocational training (e.g. student, trainee)*<br>2. *No vocational qualification and not in training*	[5]

	3. *Apprenticeship (vocational/in-company training)*<br>4. *Training at vocational school / commercial school (vocational-school training)*<br>5. *Higher vocational training / vocational schools (e.g. master craftsman or technician school)*<br>6. *University of applied sciences / higher technical colleges*<br>7. *University or college (Bachelor's, Master's or doctorate)*<br>8. *Other professional qualification, namely: ...*	
**Occupation 1**		
Are you currently...	1. *full-time employed;*<br>2. *part-time employed;*<br>3. *marginally/not employed*	[6]
**Occupation 2**		
What is your professional position?	1. *Employee*<br>2. *Manual worker*<br>3. *Civil servant*<br>4. *Farmer as main occupation*<br>5. *Self-employed (with employee)*<br>6. *Self-employed (without employees)*<br>7. *Family worker (unpaid)*<br>8. *Trainee (also interns etc.)*<br>9. *Voluntary military service / soldier*<br>10. *Voluntary gap year*<br>11. *Never been gainfully employed*	[6]
**Occupation 3**		
Do you perform management tasks in your position? (i.e. do you have authority over employees who are not trainees?)	1. *No*<br>2. *Yes, as a supervisor (instructing, supervising, controlling)*<br>3. *Yes, as a manager (authority to make decisions about personnel, budget and strategy)*	[6]
**Income 1**		
What is the approximate monthly income of your household? Please add up all income sources of all persons in your household, i.e. wages, social security benefits, scholarships, pension fund.	*Numeric input*<br>________ *CHF / EURO*	[3,7]
**Income 2**		
How many people live in your household permanently?	*Numeric input*<br>________	[3,7]
**Income 3**		
	Numeric input	[3,7]

How many of them are under the age of 14?	_______

D2.4 Survey-based: user experience

Construct/Items	Measurement	Adapted from
**Ease of Use**		
The app was easy to use	*5-point Likert scale*<br>*1 = strongly disagree, 5 = strongly agree*	[8]
**Enjoyment**		
I enjoyed using the app	*5-point Likert scale*<br>*1 = strongly disagree, 5 = strongly agree*	[8]
**Usage Intentions**		
I would use such an app for breathing exercises on a regular basis.	*5-point Likert scale*<br>*1 = strongly disagree, 5 = strongly agree*	[8]

D2.5 Survey-based: qualitative feedback

Construct/Items	Measurement	Adapted from
**Positive Feedback**		*Own item*
What did you particularly like about the app?	*Free text input*	
**Negative Feedback**		*Own item*
What did you not like at all about the app?	Free text input	

Appendix E EVALUATE results

E1 Notes on socioeconomic status (SES) index calculation

In this study, socioeconomic status (SES) is determined based on three key dimensions: education, income, and occupational status. Participants provided information on these dimensions through a LimeSurvey, as detailed in the Measurement Section and Appendix 3A. The classification of each dimension follows established frameworks:

- Education was assessed using the CASMIN classification [9]
- Income was categorized based on net household income following the OECD equivalence scale [10]
- Occupational status was measured using a simplified indicator from [11]

E1.1.1 Computation of SES Index Score

The SES index was calculated as an additive index**,** following the approach used by the German Robert Koch Institute [6,7,12]. Each of the three SES dimensions—education, income, and occupation—was assigned a point value ranging from 1 to 7 (see Appendix E1.2.2). These points were then summed to obtain an overall SES index score:

$$Points\ Education + Points\ Occupational\ Status + Points\ Income = SES\ Index\ Score$$

Each dimension was equally weighted, meaning the final SES index score could range from 3 to 21 points**.**

The points were manually assigned by two co-authors (MN and SH) according to the table depicted in Appendix E1.2.2.

The income was re-calculated in accordance with the OECD modified equivalence scale [2] and adjusted to differences in price levels between countries. To account for country-specific differences, modifications were made, particularly concerning cost of living adjustments, based on Price Level Indices for European countries. These indices compare consumer price levels across different nations relative to the EU average, ensuring comparability of income levels [3].

[2]*The equivalence values in the OECD scale are: 1.0 first adult, 0.5 second and each subsequent person aged > 14 and 0.3 to each child aged < 14. The total equivalence score is the sum of the equivalence values of all members in the household. (OECD, n.d., pp. 1-2)*

[3]The price level indices were taken from the Federal Statistical Office of Switzerland, "Price Level Indices in Europe, 2020", (https://bit.ly/3lcH6jp)

E1.1.2 Distribution of Point Values according to Lampert et al. 2018

**Points range**	**Education**	**Occupational Status** *(exemplary)*	**Income EUR**	**Income CHF** [4]
1.0-1.5	1a No school diploma and no vocational qualification ***(1.0)***	Farmer as main occupation ***(1.0)***	78-609 ***(1.0)***	111-863 ***(1.0)***
1.5-2.0	1b: Secondary school/lower secondary school diploma but no vocational qualification ***(1.7)***	-	610-821 ***(1.5)***	864-1’164 ***(1.5)***
2.0-2.5	-	Worker without supervisory/management duties ***(1.9);*** Worker (no further specification) ***(2.0)***	822-960 ***(2.0)***	1’165-1’361 ***(2.0)***
2.5-3.0	2b: Lower secondary school diploma / POS diploma but no vocational qualification ***(2.5)***	Worker with supervisory/management duties ***(2.7)***	961-1’091 ***(2.5)***	1361-1’546 ***(2.5)***
3.0-3.5	1c: No diploma from secondary school/middle school/polytechnic school but completed an apprenticeship ***(3.0)***	-	1’092-1’221 ***(3.0)***	1’547-1’731 ***(3.0)***
3.5-4.0	2a: Secondary school diploma and completed vocational training ***(3.6);*** 2c-gen: Higher education entrance qualification but no vocational qualification ***(3.7)***	Other occupations ***(3.8)***	1’222-1’344 ***(3.5)***	1’732-1’905 ***(3.5)***
4.0-4.5	-	-	1’345-1’454 ***(4.0)***	1’906-2’061 ***(4.0)***
4.5-5.0	2c-voc: Higher education entrance qualification plus vocational qualification ***(4.8)***	Employee without management/supervisory duties ***(4.4-4.8)***	1’455-1’600 ***(4.5)***	2’062-2’268 ***(4.5)***
5.0-5.5	-	Self-employed without employees ***(5.1)***	1’601-1’762 ***(5.0)***	2’268-2’497 ***(5.0)***
5.5-6.0	-	Self-employed with employees ***(5.5)***	1’763-1’971 ***(5.5)***	2’498-2’497 ***(5.5)***
6.0-6.5	Higher education degree (e.g., engineering school, applied sciences) ***(6.1)***	Employee with management duties ***(6.1)***	1’972-2’260 ***(6.0)***	2’498-3’203 ***(6.0)***
6.5-7.0	-	-	2’261-2’833 ***(6.5)***	3’204-4’016 ***(6.5)***
7.0	University or college degree ***(7.0)***	Civil servant (all categories) ***(7.0)***	> 2’834 ***(7.0)***	> 4’017 ***(7.0)***

[4] Calculation based on the Price Level Indices (PLI) by country of 2020. Price Level Index Switzerland; 159.3, Germany; 110.5, Austria; 114.3. The European Union Average equals to 100. Lichtenstein was not included in the data [13]

Calculation = (PLI CH) / (PLI GER + PLI AUT / 2) = 141.73 → the PLI of Switzerland is 41.73 % higher than in Germany and Austria. Therefore, the Income in EUR is multiplied by 1.4173 to calculate the corresponding income in CHF. (Example: 78 EUR * 1.4173 = 110.5494 ≈ 111)

E1.1.3 Classification of SES Groups

Based on the total SES index score, the population was divided into five quintiles and three status groups [6]. This approach has been successfully applied in several other studies in the German-speaking area [6,12].

Description Status	Quintile of SES	Lowest Score	Highest Score	Share in %
Low	1. Quintile	3.2	8.7	20.1%
Middle	2. Quintile	8.8	11.3	20.1%
	3. Quintile	11.4	13.7	20.5%
	4. Quintile	13.8	16.9	19.4%
High	5. Quintile	17.0	21.0	20.0%

E2 Qualitative feedback from participants

Original answers by the participants were all written in German. Please find here the translated versions:

Influencer ID	App Version	Positive Feedback	Negative Feedback
1	Breeze	-	-
2	Breeze	In short, can decide for yourself	A bit boring
2	Breeze	Chat function with short explanations<br>Quickly getting to the point	-
2	Breeze	Clear explanations	Nothing
4	StressLess	Simple design, understandable, conversation with the coach so you don't feel lonely and are calmed down	Music would be great, more info on what is stress would also be interesting
4	StressLess	Good design, easy to use.	Music missing
5	TragicKingdom	-	-
5	TragicKingdom	-	-
5	TragicKingdom	-	-
6	TragicKingdom	-	-
6	TragicKingdom	The animation and graphics were extremely well done.	-
8	StressLess	-	-
8	StressLess	Not too long, good chat	-

References

1. Russell ME, Scott AB, Boggero IA, Carlson CR. Inclusion of a rest period in diaphragmatic breathing increases high frequency heart rate variability: Implications for behavioral therapy. Psychophysiology 2017;54(3):358-365. doi:10.1111/psyp.12791
2. Steyer R, Schwenkmezger O, Notz P, Eid M. Entwicklung des Mehrdimensionalen Befindlichkeitsfragebogens (MDBF). Primärdatensatz. (Version 1.0.0) [Daten und Dokumentatio]. Göttingen: Hogrefe; 2004.
3. Thorshaug K, Schwegler C, Müller F. Wie erheben wir Geschlecht, Migrationshintergrund und sozioökonomischen Status? Methodische Ansätze zur Evaluation der Zielgruppenerreichung. Studie zuhanden der Stiftung Gesundheitsförderung Schweiz: Interface Politikstudien Forschung Beratung 2021 URL: https://gesundheitsfoerderung.ch/sites/default/files/2022-12/Arbeitspapier_059_GFCH_2021_11_-_Evaluation_Zielgruppenerreichung.pdf [accessed 2025-01-29].
4. Cullati S, Bochatay N, Rossier C, Guessous I, Burton-Jeangros C, Courvoisier DS. Does the single-item self-rated health measure the same thing across different wordings? Construct validity study. Qual Life Res 2020;29(9):2593-2604. doi:10.1007/s11136-020-02533-2
5. Thurnherr G, Egli-Keller D. So nah und doch so anders! Vergleich der Schulsysteme von Deutschland, Liechtenstein, Österreich und der Schweiz. Frankfurt; 2013.
6. Lampert T, Hoebel J, Kuntz B, Müters S, Kroll LE. Messung des sozioökonomischen Status und des subjektiven sozialen Status in KiGGS Welle 2 2018;3(1):114-133. doi:10.17886/rki-gbe-2018-016
7. Lampert T, Kroll LE. Die Messung des sozioökonomischen Status in sozialepidemiologischen Studien. In: Richter M, Hurrelmann K, editors. Gesundheitliche Ungleichheit: Grundlagen, Probleme, Perspektiven. Wiesbaden: VS Verlag für Sozialwissenschaften; 2009. ISBN:978-3-531-91643-9. p. 309–334.
8. van der Heijden H. User Acceptance of Hedonic Information Systems. MIS Quarterly 2004;28(4):695-704. doi:10.2307/25148660
9. Brauns H, Scherer S, Steinmann S. The CASMIN Educational Classification in International Comparative Research. In: Hoffmeyer-Zlotnik JHP, Wolf C, editors. Advances in Cross-National Comparison: A European Working Book for Demographic and Socio-Economic Variables. Boston, MA: Springer US; 2003. p. 221–244 doi:10.1007/978-1-4419-9186-7_11
10. OECD. What are Equivalence Scores: OECD Project on Income Distribution and Poverty n.d URL: https://www.oecd.org/els/soc/OECD-Note-EquivalenceScales.pdf [accessed 2024-12-31].
11. Hoffmeyer-Zlotnik J, Geis A. Berufsklassifikation und Messung des beruflichen Status/ Prestige. ZUMA Nachrichten 2003;52.
12. Lampert T, Kroll LE, Müters S, Stolzenberg H. Messung des sozioökonomischen Status in der Studie „Gesundheit in Deutschland aktuell“ (GEDA). Bundesgesundheitsblatt - Gesundheitsforschung - Gesundheitsschutz 2013;56(1):131-143. doi:10.1007/s00103-012-1583-3
13. Federal Statistical Office. Price level indices n.d. URL: https://www.bfs.admin.ch/bfs/en/home/statistics/prices/international-price-comparisons/price-level-indices.html.
